# Bombesin-Targeted Delivery of β-Carboline-Based Ir(III) and Ru(II) Photosensitizers for a Selective Photodynamic Therapy of Prostate Cancer

**DOI:** 10.1021/acs.inorgchem.4c02583

**Published:** 2024-10-03

**Authors:** Juan Sanz-Villafruela, Cristina Bermejo-Casadesús, Gerard Riesco-Llach, Mònica Iglesias, Marta Martínez-Alonso, Marta Planas, Lidia Feliu, Gustavo Espino, Anna Massaguer

**Affiliations:** † Universidad de Burgos, Departamento de Química, Facultad de Ciencias, Plaza Misael Bañuelos s/n, Burgos 09001, Spain; ‡ Universitat de Girona, Departament de Biologia, Facultat de Ciències, Maria Aurelia Capmany 40, Girona 17003, Spain; § LIPPSO, Departament de Química, Facultat de Ciències, Universitat de Girona, Maria Aurelia Capmany 69, Girona 17003, Spain; ∥ Universitat de Girona, Departament de Química, Facultat de Ciències, Maria Aurelia Capmany 69, Girona 17003, Spain

## Abstract

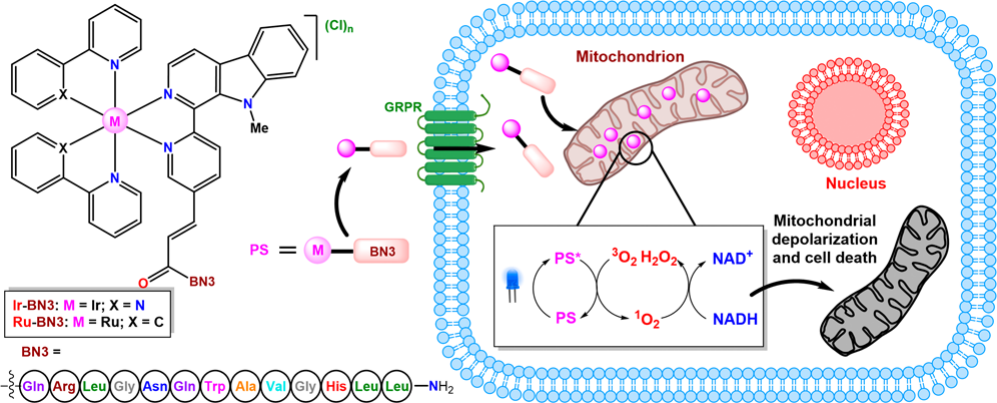

Despite advances in Ir(III) and Ru(II) photosensitizers (PSs), their lack of selectivity for cancer cells has hindered their use in photodynamic therapy (PDT). We disclose the synthesis and characterization of two pairs of Ir(III) and Ru(II) polypyridyl complexes bearing two β-carboline ligands (N^N’) functionalized with −COOMe (**L1**) or −COOH (**L2**), resulting in PSs of formulas [Ir(C^N)_2_(N^N’)]Cl (**Ir-Me:** C^N = ppy, N^N’ = **L1**; **Ir-H:** C^N = ppy, N^N’ = **L2**) and [Ru(N^N)_2_(N^N’)](Cl)_2_ (**Ru-Me:** N^N = bpy, N^N’ = **L1; Ru-H:** N^N = bpy, N^N’ = **L2**). To enhance their selectivity toward cancer cells, **Ir-H** and **Ru-H** were coupled to a bombesin derivative (**BN3**), resulting in the metallopeptides **Ir-BN** and **Ru-BN**. Ir(III) complexes showed higher anticancer activity than their Ru(II) counterparts, particularly upon blue light irradiation, but lacked cancer cell selectivity. In contrast, **Ir-BN** and **Ru-BN** exhibited selective photocytoxicity against prostate cancer cells, with a lower effect against nonmalignant fibroblasts. All compounds generated ROS and induced severe mitochondrial toxicity upon photoactivation, leading to apoptosis. Additionally, the ability of **Ir-Me** to oxidize NADH was demonstrated, suggesting a mechanism for mitochondrial damage. Our findings indicated that the conjugation of metal PSs with **BN3** creates efficient PDT agents, achieving selectivity through targeting bombesin receptors and local photoactivation.

## Abstract 2

We have synthesized and characterized Ir(III) and Ru(II) polypyridyl complexes with β-carboline ligands functionalized with −COOMe or −COOH as photosensitizers. These complexes were coupled to a bombesin derivative (**BN3**) producing **Ir-BN** and **Ru-BN** metallopeptides. The Ir(III) complexes exhibited higher anticancer activity than the Ru(II) counterparts, particularly under blue light, but lacked selectivity. **Ir-BN** and **Ru-BN** metallopeptides, however, selectively targeted prostate cancer cells, causing mitochondrial toxicity and apoptosis upon photoactivation, demonstrating their potential for targeted PDT.

## Introduction

In the last few decades, cancer has become a leading cause of death worldwide, with a significant impact on our society. Specifically, prostate cancer is the second most common cancer and the fifth leading cause of cancer death in men, with an estimated 1.4 million new cases and 375000 deaths worldwide by 2020. The main treatment options for localized prostate cancer are surgery and radiotherapy. For cases of recurrent or metastatic disease, the most used therapies are androgen deprivation and chemotherapy.^1−3^ However, one of the main challenges in the management of these patients is the development of drug resistance.^4,5^ New strategies are being developed to overcome these limitations resulting in an increased attention toward photodynamic therapy (PDT). This minimal invasive and clinically approved therapy is based on the use of a photosensitizer (PS) which is electronically excited under light irradiation to form a short-lived excited state (PS*) that can react with molecular oxygen to generate reactive oxygen species (ROS). Ultimately, these ROS damage essential biomolecules and kill cancer cells, leading to the disappearance of the target tumor.^6^ Moreover, because of the spatiotemporal control on the excitation of the PS, this therapy is highly precise compared to chemotherapy.^7^ Ideally, the PS must display excellent photostability, low cytotoxicity in dark conditions, absorption bands in the therapeutic window (600–850 nm),^8^ selective accumulation in the tumor tissue, efficient ROS generation, and fast clearance from the body.^9,10^ Nonetheless, clinically approved PSs, such as Photofrin, exhibit limitations and are far from ideal.^11^ As a result, there is a need to develop new PSs that pave the way toward a wider clinical use of PDT.

During the last few years, there have been significant advances in developing novel Ir(III) and Ru(II) polypyridyl PSs.^12,13^ These complexes display characteristics that make them suitable for being used in PDT: (1) They display absorption bands in the visible region; (2) the presence of a heavy metal center allows high spin–orbit coupling constants leading to a fast and efficient population of the triplet excited states, which results in longer lifetimes and higher singlet oxygen quantum yields;^14^ (3) they usually display high photostability;^15^ (4) their photophysical and biological properties can be easily modulated through the functionalization or modification of the ligands;^12,13^ (5) they can generate different ROS, and some of them are active in hypoxic conditions;^16,17^ (6) in the excited state, they can act as strong oxidants or reductants reacting with a wide variety of substrates.^18,19^ More specifically, β-carboline-based Ir(III) and Ru(II) complexes are being studied as potential PDT agents with promising results, although they display high or moderate cytotoxicity toward nonmalignant cells in dark conditions.^20−22^ Hence, it is important to develop strategies to specifically deliver these complexes to tumor cells. Targeted delivery systems not only minimize the impact of the anticancer agents on normal tissues but also optimize their accumulation and activity at the tumor site, lowering the PS doses and the number of light exposure cycles, which in turn limits the patient photosensitivity. One successful strategy is the conjugation of complexes to carrier peptides, whose receptors are overexpressed in cancer cells.^23,24^ Peptides offer several advantages over other tumor-targeting agents, such as proteins and monoclonal antibodies, highlighting their small size, high ability to penetrate tumors, and good biocompatibility. Furthermore, they can be synthesized and modified in a relatively simple manner. The use of carrier peptides for targeted drug delivery has been shown to be effective in prostate cancer, where a high percentage of primary tumors and bone metastases have been found to overexpress the gastrin-releasing peptide receptor (GRPR).^25−28^ GRPR is also overexpressed in other human cancers, including breast, colorectal, lung, or pancreatic, while it is poorly expressed in healthy tissues, making it an attractive target for selective cancer treatment.^29,30,31^

One of the natural ligands of GRPR in humans is the gastrin-releasing peptide (GRP), a neuropeptide that regulates multiple physiological functions. Additionally, GRP acts as a mitogen and proangiogenic factor in different cancers.^30^ Bombesin (BN) is a 14-amino acid peptide (Pyr-Gln-Arg-Leu-Gly-Asn-Gln-Trp-Ala-Val-Gly-His-Leu-Met-NH_2_) that shares a homologous seven-amino acid C-terminal region with GRP. Due to its high affinity for GRPR, BN has been demonstrated to be an effective carrier peptide for targeted delivery of drugs and diagnostic agents to GRPR-overexpressing tumors.^28,32,33^ This strategy was initially employed in nuclear medicine to deliver ^99m^Tc complexes to cancer cells.^34−36^ More recently, different PSs have been conjugated to BN analogues in an effort to direct their photodynamic activity against cancer cells, while minimizing off-target effects on healthy tissues.^37−42^ In a previous work, we synthesized a series of BN derivatives with the aim of identifying the most effective sequence for delivering Pt(II) and Ru(II) complexes to prostate cancer cells. Among the peptides developed, Gln-Arg-Leu-Gly-Asn-Gln-Trp-Ala-Val-Gly-His-Leu-Leu-NH_2_ (**BN3**) exhibited the most effective tumor-targeting properties, and the resulting **BN3**-derived metallopeptides displayed high anticancer activity and reduced toxicity toward nonmalignant fibroblasts.^38^

Inspired by our previous works, and considering the need to develop new metal-based PSs, we selected **BN3** as a tumor-homing peptide with the objective of improving the selectivity and biocompatibility of our new β-carboline-based Ir(III) and Ru(II) PSs. Thus, we aimed to endow the resulting metallopeptides with two levels of selectivity in their anticancer action, the first derived from the targeting properties of **BN3** and the second associated with the photoactivation ability of the metal fragments.

In this study, we disclose the conjugation of different β-carboline-based Ir(III) and Ru(II) PSs to **BN3** and the evaluation of the effect of this peptide on the selectivity and photocytotoxicity of the conjugated complexes. Particular attention is paid to the influence of the metal fragment on the anticancer activity of the metallopeptides.

## Results and Discussion

### Synthesis of Ligands and Complexes

**Scheme 1:**
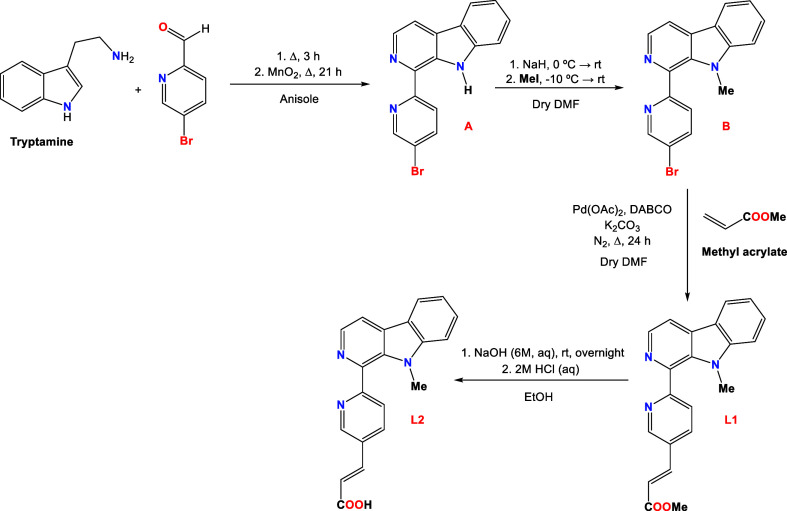
Synthetic Route for the Synthesis of **L1** and **L2**

**Scheme 2:**
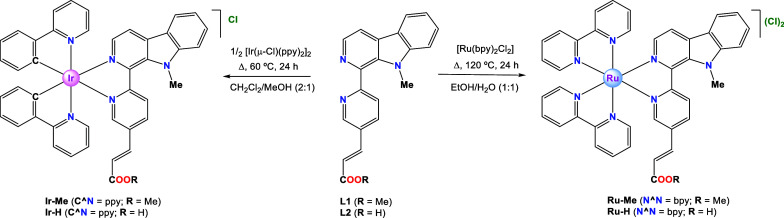
Synthesis and Molecular Structure of Complexes **Ir-Me**, **Ir-H**, **Ru-Me**, and **Ru-H**

Two pairs of Ir and Ru complexes with two different N^N’ ligands (**L1** and **L2**) and general formulas [Ir(C^N)_2_(N^N’)]Cl (**Ir-Me:** C^N = ppy, N^N’ = **L1**; **Ir-H:** C^N = ppy, N^N’ = **L2**) and [Ru(N^N)_2_(N^N’)](Cl)_2_ (**Ru-Me:** N^N = bpy, N^N’ = **L1; Ru-H:** N^N = bpy, N^N’ = **L2**) have been prepared aiming to evaluate the effect of the metal fragment on their photophysical and biological properties (Schemes 1 and 2). Moreover, complexes **Ir-H** and **Ru-H** have been prepared with a −COOH group to allow their conjugation to the bombesin derivative **BN3**. The ancillary ligands (**L1**-**L2**) were synthesized in several steps according to Scheme 1. First, we reacted tryptamine and 5-bromo-2-pyridinecarboxaldehyde to obtain pyridyl-β-carboline **A**, following a method previously described for similar compounds.^43^ Then, the pyrrolic N–H group was substituted with a methyl group to obtain derivative **B**, employing NaH as the base and MeI as the methylating agent.^22^ Subsequently, a Heck vinylation protocol was performed on the bromopyridyl ring of **B**, using methyl acrylate to obtain **L1**. Furthermore, the ester group was hydrolyzed in alkaline media and neutralized with HCl to obtain **L2**, bearing a −COOH group.

The Ir(III) complexes of general formula *rac*-[Ir(ppy)_2_(N^N’)]Cl (ppy = 2-phenylpyridinate and N^N’ = **L1** or **L2**) were prepared through a bridge splitting reaction between the dimeric Ir(III) precursor *rac*-[Ir(μ-Cl)(ppy)]_2_ and the corresponding β-carboline-based ligand (**L1** or **L2**, using a molar ratio 1:2, dimer:ligand) to obtain **Ir-Me** and **Ir-H**, respectively (Scheme 2). Ru(II) polypyridyl complexes of general formula *rac*-[Ru(bpy)_2_(N^N’)](Cl)_2_ (bpy = 2,2′-bipyridine and N^N’ = **L1** or **L2**) were prepared by heating the Ru(II) precursor, *rac-*[Ru(bpy)_2_Cl_2_], with **L1** or **L2** (1:1 molar ratio), to obtain **Ru-Me** and **Ru-H**, respectively (Scheme 2). The metal complexes were obtained as racemic mixtures (Λ,Δ) of the respective chloride salts. Ligands and complexes were fully characterized by nuclear magnetic resonance (NMR), mass spectrometry, and elemental analysis (see Figures S1–S32). In particular, ^1^H NMR spectra of all the complexes were recorded in DMSO-*d*_6_ at 25 °C. The spectra displayed two sets of resonances for the two nonequivalent ppy/bpy ligands owing to the lack of symmetry shown by these derivatives. All of them showed a singlet around 3.93 ppm attributed to the N-Me group, which is shifted to lower field compared to the corresponding singlet of the free ligand (3.60 ppm). The high-resolution electrospray ionization mass spectrometry (HR ESI(+) MS) spectra exhibited peaks with mass/charge ratios and isotopic distributions fully compatible with those calculated for either the monocationic Ir complexes or the dicationic Ru complexes. The analytical data obtained from elemental analysis are in good agreement with expected values.

### Synthesis of the Metallopeptides

**Scheme 3:**
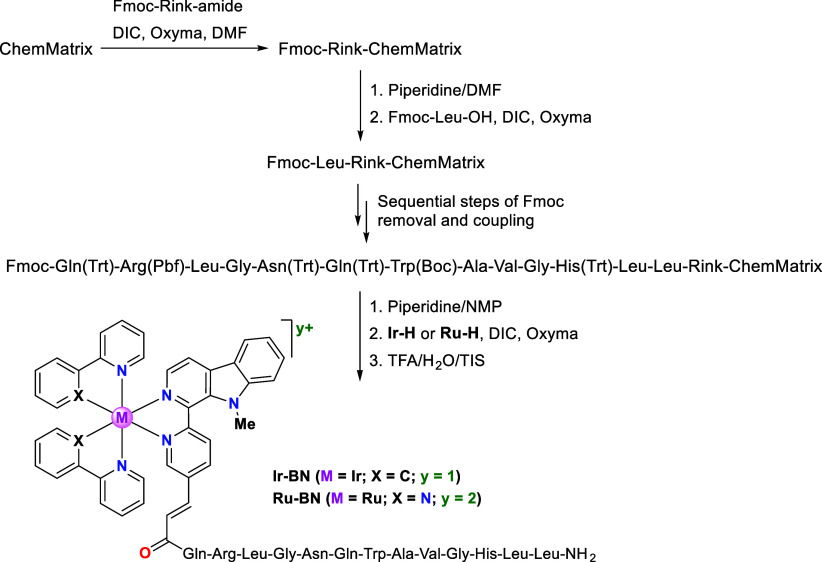
Solid-Phase Synthesis of Metallopeptides **Ir-BN** and **Ru-BN**

Metallopeptides **Ir-BN** and **Ru-BN** incorporating the Ir(III) or the Ru(II) complex at the N-terminus of the peptide **BN3** were synthesized on solid phase following a standard 9-fluorenylmethoxycarbonyl (Fmoc)/*tert*-butyl (*t*Bu) strategy (Scheme 3).^38^ An aminomethyl ChemMatrix resin was used as solid support to which the Fmoc-Rink-amide linker was incorporated leading to C-terminal amidated peptides. The attachment of this linker to the ChemMatrix resin was accomplished using *N,N*-diisopropylcarbodiimide (DIC) as coupling reagent, ethyl (hydroxyimino)cyanoacetate (Oxyma) as additive, and dimethylformamide (DMF) as solvent. The elongation of the peptide sequence was performed through sequential steps of Fmoc group removal with piperidine/DMF (3:7) and coupling of the corresponding amino acids using DIC and Oxyma in DMF. A Kaiser test was performed to confirm the completion of the coupling reactions.^44^ Once the peptide sequence was completed, the N-terminal Fmoc group was removed, and the corresponding metal complex **Ir-H** or **Ru-H** was incorporated in the presence of DIC and Oxyma in DMSO. Next, the resulting metallopeptides **Ir-BN** and **Ru-BN** were cleaved from the support by acidolytic treatment with trifluoroacetic acid (TFA)/H_2_O/triisopropylsilane (TIS), purified by reversed-phase column chromatography, analyzed by HPLC, and characterized by mass spectrometry. **Ir-BN** and **Ru-BN** were obtained in >99% HPLC purity (see Figures S33–44).

### X-ray Diffraction

**Figure 1:**
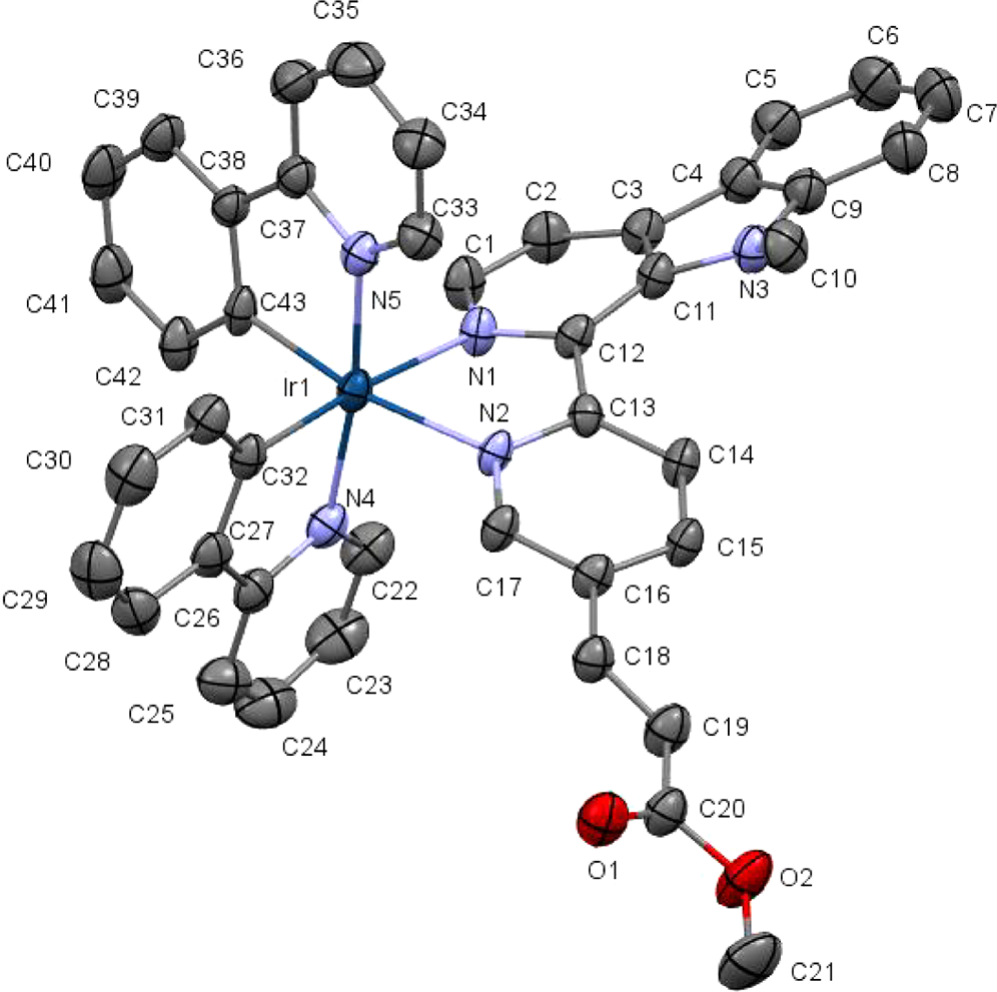
ORTEP diagram for the molecular structure of **Λ-[Ir-Me]**^**+**^ obtained by single-crystal X-ray diffraction. Thermal ellipsoids are shown at the 30% probability level. The respective Δ enantiomer, H atoms, and the PF_6_^–^ counterion have been omitted for the sake of clarity.

The crystal structure of [**Ir-Me**]PF_6_ was resolved by single-crystal X-ray diffraction. A suitable single crystal was obtained by slow evaporation of an acetonitrile solution of [**Ir-Me**]Cl in the presence of NH_4_PF_6_. The complex crystallizes in the monoclinic *C*2/*c* space group. Due to helical chirality, the unit cell contains two pairs of enantiomers (Δ,Λ) (Figure S45). The ORTEP diagram for the molecular structure of **Λ-[Ir-Me]**^**+**^ is shown in Figure 1. Selected bond distances and angles are compiled in Table 1, and important crystallographic parameters are gathered in Table S3.

**Table 1:** Selected Bond Distances (Å) and Coordination Angles for *rac***-[Ir-Me]**^**+**^

*rac*-[Ir-Me]^+^	Distances	*rac*-[Ir-Me]^+^	Angles (deg)
Ir(1)–N(1)	2.153 (4)	N(1)Ir(1)N(2)	76.79 (14)
Ir(1)–N(2)	2.159 (3)	C(32)Ir(1)N(4)	80.62 (19)
Ir(1)–N(4)	2.045 (4)	C(43)Ir(1)N(5)	80.3 (2)
Ir(1)–C(32)	2.021 (5)		
Ir(1)–N(5)	2.046 (4)		
Ir(1)–C(43)	2.021 (4)		

The molecular structure of **[Ir-Me]**^**+**^ exhibited the expected pseudo-octahedral geometry with the predictable *trans*-N,N and *cis*-C,C disposition for the two cyclometalated ligands (ppy). The Ir–N bond distances for the N^N’ ligand (2.153(4), 2.159(3) Å) were longer than those determined for the ppy ligands (2.046(4), 2.045(45) Å), due to the strong *trans* influence attributed to the metal-bonded phenyl rings. Both Ir–C bond lengths were nearly identical (2.021(5) and 2.021(5)) and within the expected range for this kind of complexes. The bite angles of the chelate rings were also standard, that is, 76.79° for **L1** and 80.62°, 80.30° for the ppy ligands. The N^N’ ligand displayed a high torsion angle (−18.55°) in comparison with the ppy ligands (2.07° and 3.04°) revealing a lower degree of coplanarity, due to the high steric hindrance between the N-Me group and the pyridyl ring. Furthermore, the crystal structure exhibited different weak hydrogen-bonding interactions between fluorine atoms from the counterion and different hydrogen donor groups of the iridium complex. Also, a weak hydrogen-bonding interaction between an oxygen atom (O1) from **L1** and one hydrogen (H23) from a ppy ligand of a neighboring molecule was observed (Figure S46 and Table S4).

### UV–Vis Spectroscopy

**Figure 2:**
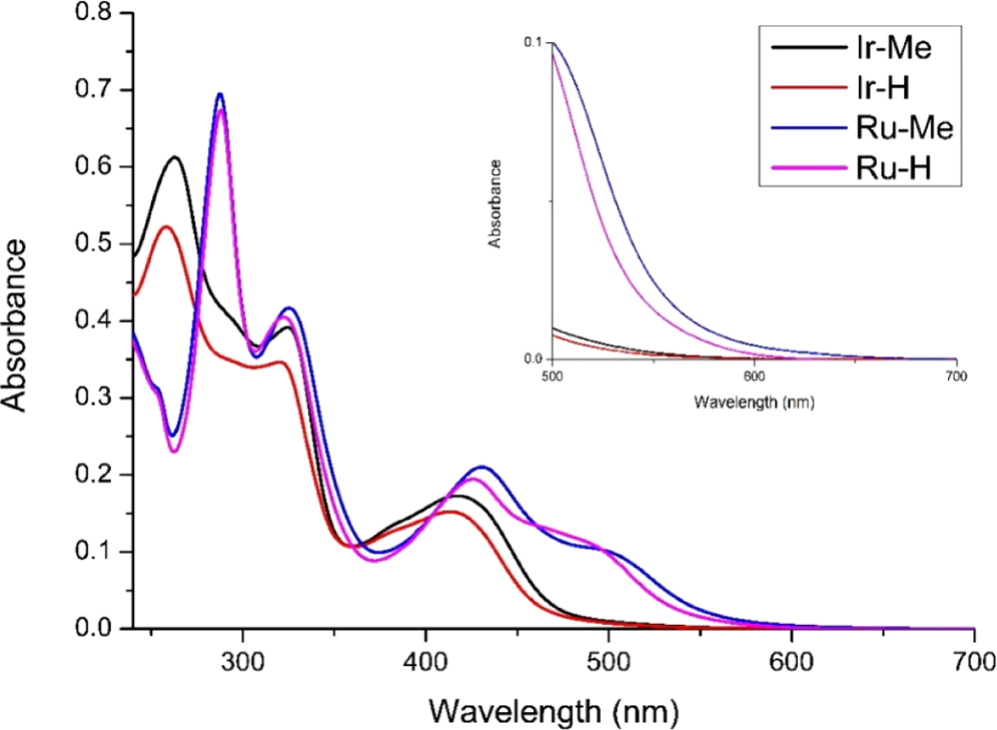
Overlaid absorbance spectra of Ir(III)/Ru(II) complexes in H_2_O:DMSO (99:1, v:v) (10^–5^ M) at room temperature. Inset: zoom of the region between 500 and 700 nm.

The UV–vis absorption spectra of **Ir-H**, **Ir-Me**, **Ru-H**, and **Ru-Me** (Figure 2) were recorded at room temperature for H_2_O:DMSO (99:1) solutions (10^–5^ M). All the complexes displayed strong absorption bands with maxima between 250 and 350 nm that were attributed to spin-permitted ligand centered transitions (^1^LC, π → π*). In the visible region, Ir(III) complexes showed a main absorption band with maxima around 420 nm that extends up to 550 nm and corresponds to mixed spin-allowed and spin-forbidden metal to ligand charge transfer (^1^MLCT and ^3^MLCT) and ligand to ligand charge transfer (LLCT) transitions. By contrast, the Ru(II) complexes showed two absorption bands in the visible region, with maxima at 430 and 500 nm and a tail extended up to 600 nm. These bands were also ascribed to ^1^MLCT and ^3^MLCT and LLCT transitions. Therefore, the Ru(II) derivatives exhibited red-shifted bands compared to their Ir(III) analogues. Moreover, complexes with the methyl ester group **Ir-Me**/**Ru-Me** showed absorption bands slightly red-shifted relative to the complexes with −COOH groups, **Ir-H**/**Ru-H**.

### Emission and Photophysical Properties

**Figure 3:**
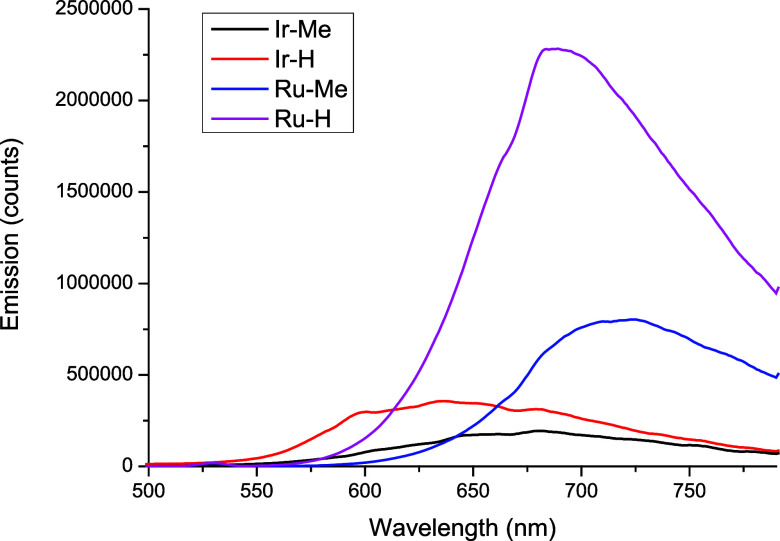
Overlaid emission spectra of **Ir(III)/Ru(II)** complexes in deoxygenated H_2_O:DMSO (99:1, v:v, 10^–5^ M) at 25 °C.

The emission spectra of the metal complexes in deoxygenated solutions of H_2_O:DMSO (99:1, v:v, 10^–5^ M) were recorded at 25 °C, using λ_ex_ of 405 nm for **Ir-Me**/**Ir-H** and λ_ex_ of 450 nm for **Ru-Me**/**Ru-H** (Figure 3). All the complexes displayed a single broad emission band with maxima between 651 and 720 nm and large Stokes shifts (λ_em_ – λ_ex_ > 230 nm), which corroborated the phosphorescent nature of the emission. The Ru(II) complexes exhibited higher emission intensities, and their respective maxima are red-shifted compared to those of the Ir(III) analogues. Moreover, **Ir-Me**/**Ru-Me** featured lower emission intensities compared to **Ir-H**/**Ru-H**, respectively.

The excited state lifetimes (τ) and the photoluminescence quantum yields were also determined for the new metal complexes (Table 2). The Ir PSs displayed two-component τ values of 2 ns [20%]/93 ns [80%] and 23 ns [51%]/145 ns [49%] for **Ir-Me** and **Ir-H**, respectively. By contrast, the Ru(II) complexes exhibited one-component τ values of 74 and 186 ns for **Ru-Me** and **Ru-H**, respectively. These values compare well with those of similar complexes and are compatible with a triplet nature for the emissive states. The photoluminescence quantum yields (Φ_PL_) of Ir(III)/Ru(II) were very low with values ranging from 1.1 to 4.13%.

**Table 2:** Photophysical Properties of the Metal Complexes in H_2_O:DMSO (99:1, v:v) at 25 °C under a Nitrogen Atmosphere

Compound	λ_ex_ (nm)	λ_em_ (nm)	τ (ns) [contribution (%)]	Φ_PL_ (%)
Ir-Me	405	676	2 [20] 93 [80]	3.50
Ir-H	405	651	23 [51] 145 [49]	4.13
Ru-Me	450	720	74	1.10
Ru-H	450	688	186	3.13

### Determination of p*K*_a_

**Figure 4:**
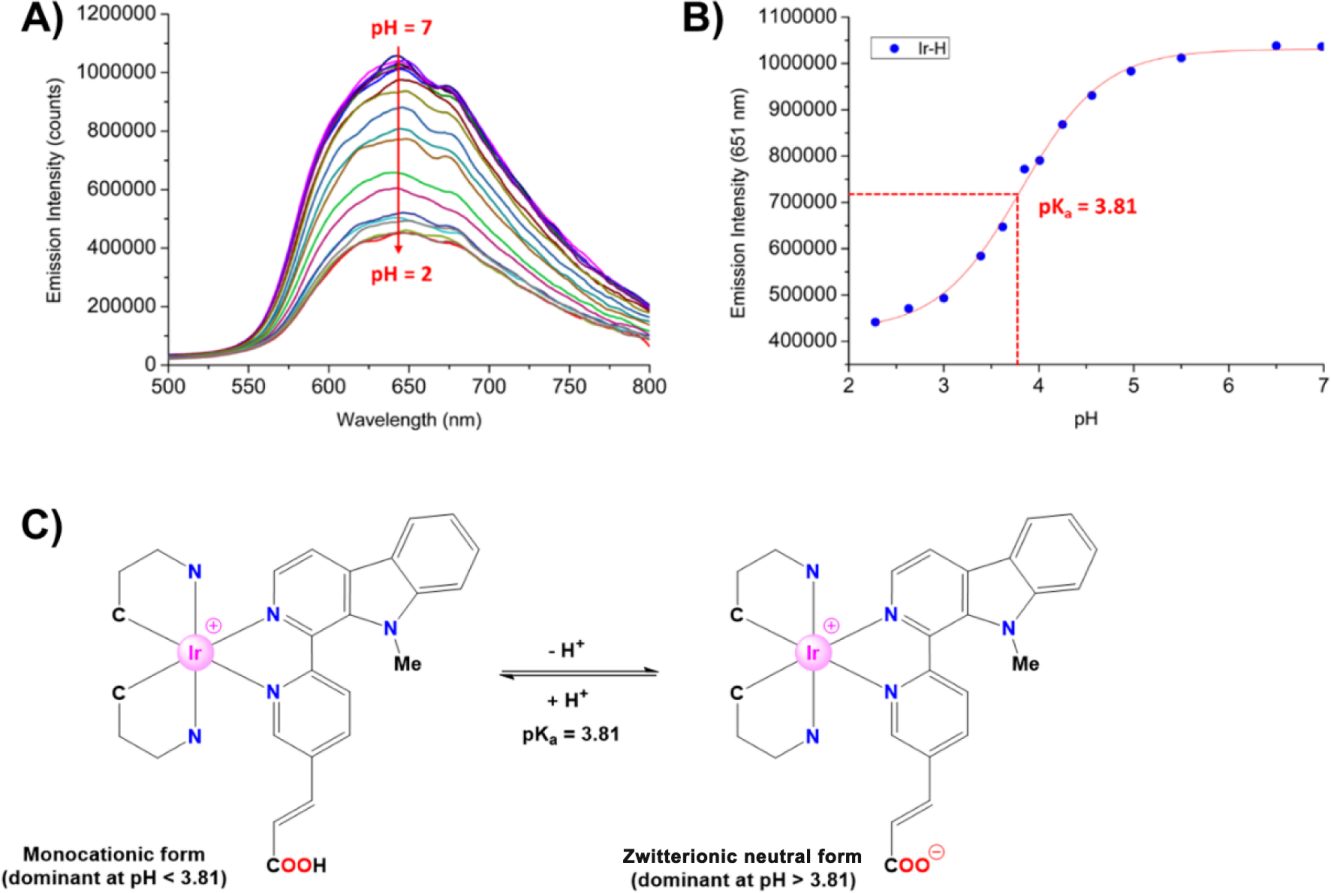
p*K*_a_ determination. (A) Overlaid emission spectra of **Ir-H** in H_2_O:DMSO (99:1, v:v) (10^–5^ M) at different pH values ranging from pH 7 to 2 (HCl titration). (B) Plot of the emission intensity of **Ir-H** at λ 651 nm in H_2_O:DMSO (99:1, v:v) (10^–5^ M) as a function of pH (2–7) at 25 °C. (C) Scheme showing the acid–base equilibrium for **Ir-H**.

**Ir-H** and **Ru-H** are susceptible to deprotonation in aqueous media due to the presence of the acidic −COOH group. In fact, the actual protonation state of these complexes at the different physiological pH values defines their global charge and correspondingly influences their solubility, their photophysical properties, and their cellular uptake.^45−47^ Thus, the p*K*_a_ values of **Ir-H** and **Ru-H** in their excited states were experimentally determined by monitoring the variations of their emission intensity at specific wavelengths versus different pH values and fitting the data (*I*_em,λ_/pH) to a sigmoidal equation. In good agreement with similar complexes reported in the literature,^45,46^ there was a gradual decrease in the emission intensity of **Ir-H** and **Ru-H** with decreasing pH (Figures 4 and S50). The p*K*_a_ values for **Ir-H** and **Ru-H** in their excited states were 3.81 and 4.51, respectively. These values can be considered as an acceptable approximation to the values of the respective ground states. Therefore, we concluded that **Ir-H** and **Ru-H** adopt their deprotonated zwitterionic forms in the whole range of physiological pHs. This means that **Ir-H** assumes a zwitterionic neutral form, while **Ru-H** adopts a zwitterionic monocationic structure. In both cases, there is charge separation, and as a result, we predict that the cellular uptake ability of these complexes could be reduced, in comparison to that of their relatives **Ir-Me** and **Ru-Me**.

### Photostability of Ir(III)/Ru(II) Complexes

**Figure 5:**
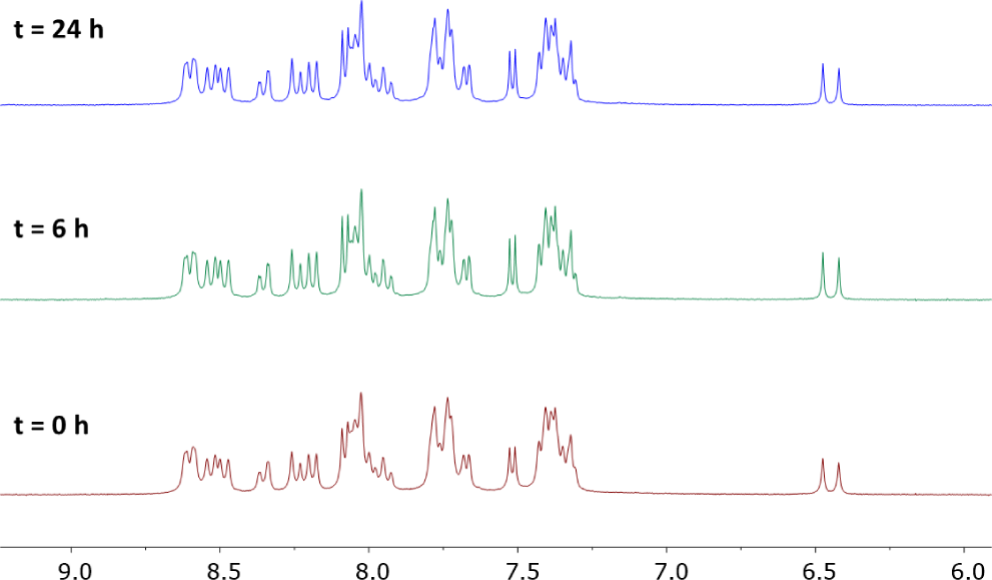
Evolution of the aromatic region of the ^1^H NMR spectra of **Ru-H** (1.5 × 10^–2^ M) in DMSO-*d*_6_:D_2_O (3:2, v:v) under blue light irradiation (LED, λ = 460 nm, 24 W) at different times.

Photostability is a desirable feature for PDT agents, since otherwise, their efficiency as PSs can be decreased owing to so-called photobleaching. Thus, the photostability of aerated solutions (1.5 × 10^–2^ M, DMSO-*d*_6_:D_2_O, 3:2, v:v) of all our complexes was evaluated by ^1^H NMR. The solutions were exposed to blue light irradiation (λ_irr_ = 460 nm, 24 W), and the evolution of the respective samples was monitored by ^1^H NMR at different times (0, 6, and 24 h) at room temperature. To our delight, no symptoms of photodegradation were observed, confirming that all the complexes exhibited outstanding photostability under the aforementioned conditions (Figures 5 and S47–S49).

### Photocatalytic Generation of Singlet Oxygen

**Figure 6:**
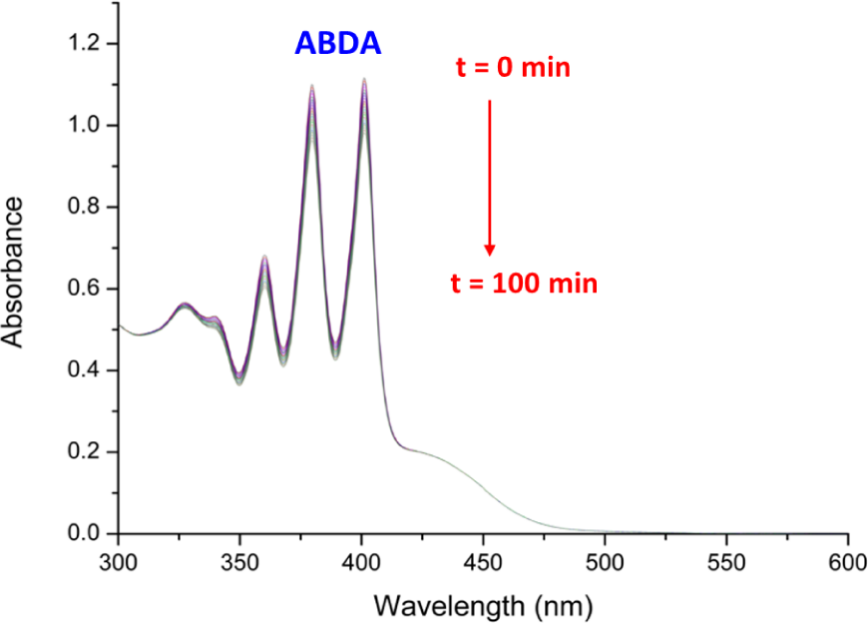
Photobleaching of ABDA (8 × 10^–5^ M) in the presence of **Ir-Me** (10^–5^ M) using H_2_O:DMSO (1:1) under blue light irradiation (460 nm, 24 W) during 100 min at room temperature.

Singlet oxygen is thought to be the main cytotoxic species in type II PDT processes. Therefore, we determined the ability of **Ir-Me** and **Ru-Me** to generate ^1^O_2_ under photocatalytic conditions, as illustrative examples of our PSs. As shown in Figures 6 and S51, we monitored by means of UV–vis spectroscopy the oxidation of 9,10-anthracenediyl bis(methylene)dimalonic acid (ABDA, 8x 10^–5^ M) in the presence of atmospheric oxygen, using **Ir-Me** (10^–5^ M) as the PS and H_2_O:DMSO (1:1) as the solvent system under blue light exposure (λ_ir_ = 460 nm). ABDA is a very specific probe for ^1^O_2_, since it reacts selectively with the in situ produced ^1^O_2_ to generate the respective endoperoxide, which is characterized by a loss of π-extended aromaticity and the disappearance of several absorption bands in the UV–vis region (i.e., 379 nm). Moreover, the singlet oxygen quantum yield values (**Φ**_Δ_) of **Ir-Me** and **Ru-Me** were determined using rose bengal (**Φ**_Δ_ = 0.75) as the reference. As expected, both **Ir-Me** and **Ru-Me** exhibited photocatalytic activity in the generation of ^1^O_2_, but their respective **Φ**_Δ_ were low, 4 and 9%, in agreement with its moderate excited state lifetimes (93 and 74 ns). A control experiment in the absence of PS was also performed resulting in a negligible conversion.

### Photocatalytic Oxidation of NADH

NADH is an enzymatic cofactor playing a main role as an electron donor in the mitochondrial electron transport chain. Hence, it is considered a potential molecular target for those anticancer drugs that cause oxidative stress in mitochondria. Indeed, several research groups have recently established a relationship between the photocatalytic oxidation of NADH to NAD^+^, the depolarization of the mitochondrial membrane and apoptotic cell death.^48,49^

**Figure 7:**
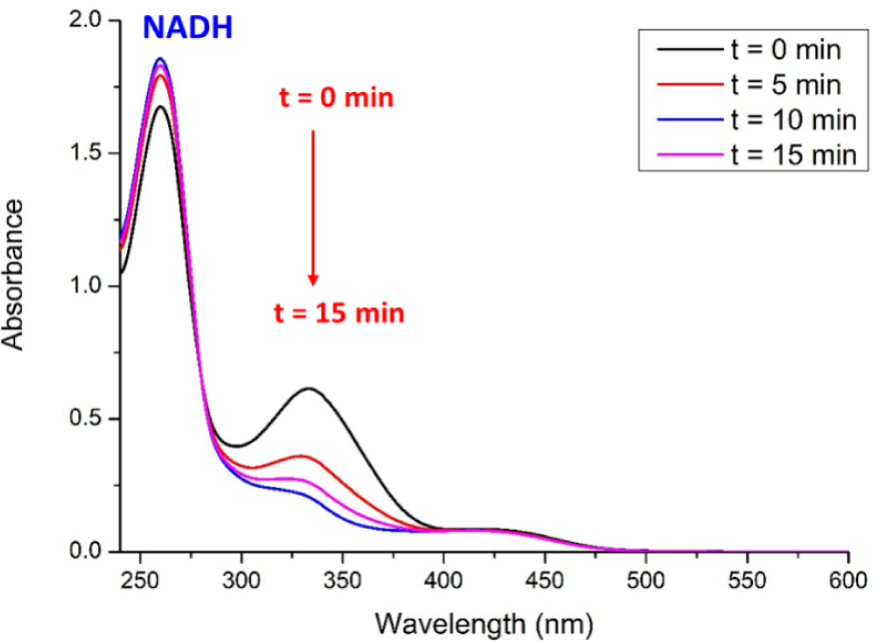
Evolution of the UV–vis spectra during the photocatalytic oxidation of NADH (100 μM) in the presence of **Ir-Me** (5 μM) in aerated H_2_O:DMSO (99:1) under blue light irradiation (460 nm, 24 W) at room temperature (15 min).

Aiming to prove this hypothesis for **Ir-Me**, as a model PS, we monitored by UV–vis spectroscopy the photocatalytic oxidation of an aerated solution of NADH (0.1 mM) in the presence of **Ir-Me** (5 μM) using H_2_O:DMSO (99:1) under blue light irradiation (λ_ir_ = 460 nm) for 15 min. As a result, we observed the decrease of the band due to NADH at 338 nm (Figure 7). This evolution is compatible with the photocatalyzed formation of NAD^+^. Two control experiments in the absence of either **Ir-Me** (Figure S52) or light (Figure S53) corroborated the photocatalytic character of this transformation.

### Effect on Cell Viability

The effect of **Ir-H**, **Ir-Me**, **Ru-H**, and **Ru-Me** on cell viability was evaluated in the A549 lung cancer and PC-3 prostate cancer cell lines by MTT assays. PC-3 cells were specifically chosen due to their high expression levels of GRPR on their surface, allowing for further evaluation of the activity of the **BN3**-derived metallopeptides **Ir-BN** and **Ru-BN**.^38,51^ In contrast, A549 cells express low levels of GRPR.^52^ MRC-5 fibroblasts were used as a nonmalignant cell model.^53^Table 3 reports the half maximal inhibitory concentration (IC_50_) values obtained after treating the cells either under dark conditions or upon exposure to blue light at a dose of 24.1 J cm^–2^. The photocytotoxicity index (PI), defined as the IC_50,dark_/IC_50,light_, is provided for each compound.

**Table 3:** Photocytotoxic Effects of Complexes **Ir-H**, **Ir-Me**, **Ru-H**, and **Ru-Me** and of Metallopeptides **Ir-BN** and **Ru-BN** on Different Cell Lines in the Dark and after Blue Light Irradiation

IC_50_^a^ (μM)									
	A549			PC-3			MRC-5		
	Dark	Light	PI^b^	Dark	Light	PI^b^	Dark	Light	PI^b^
**Ir-H**	35.0 ± 5.4	3.32 ± 1.1	10.5	32.2 ± 1.9	9.23 ± 0.63	3.5	39.1 ± 2.4	6.46 ± 0.801	6.1
**Ir-Me**	0.471 ± 0.031	0.0334 ± 0.0068	14.2	1.17 ± 0.29	0.0723 ± 0.031	16.2	1.12 ± 0.31	0.0313 ± 0.019	35.8
**Ru-H**	67.8 ± 6.7	56.7 ± 1.6	1.2	70.4 ± 2.7	34.8 ± 4.3	2.0	53.9 ± 5.4	43.5 ± 4.5	1.2
**Ru-Me**	74.7 ± 2.03	33.8 ± 5.2	2.2	76.8 ± 2.1	48.6 ± 0.5	1.6	62.5 ± 2.6	43.7 ± 7.2	1.4
**Ir-BN**	68.3 ± 15.8	12.6 ± 1.3	5.4	35.3 ± 3.8	8.51 ± 0.8	4.1	80.9 ± 1.3	28.7 ± 5.9	2.8
**Ru-BN**	49.5 ± 11.9	23.6 ± 11.2	2.1	41.3 ± 1.7	4.11 ± 1.2	10.1	>100	>100	n.d.
**Cisplatin**	5.99 ± 1.2	n.d.	n.d.	5.55 ± 0.97	n.d.	n.d.	5.34 ± 0.38	n.d.	n.d.

a Cells were incubated with the compounds for 4 h at 37°C and then kept in the dark or exposed to blue light irradiation for 1 h (460 nm, 24.1 J cm^–2^). Cell viability was assessed after 48 h of treatment by MTT assays. Data represent the mean ± SD of at least three independent experiments, each performed in triplicate.

b Photocytotoxic index (PI) = IC_50, dark_/IC_50, light_. n.d.: not determined.

The Ir(III) complexes exhibited a greater effect on cell viability than their Ru(II) counterparts in all cell lines. Among them, **Ir-Me** displayed the highest anticancer activity, with IC_50_ values of 0.471 μM in A549 cells and 1.17 μM in PC-3 cells in dark conditions, which were lower than those of cisplatin under the same experimental conditions. Conversely, **Ir-H** displayed IC_50,dark_ values above 30 μM, revealing that the esterification of the β-carboline ligand with a methyl group significantly enhanced the biological activity of the resulting **Ir-Me** complex. Upon blue light irradiation, the cytotoxicity of **Ir-Me** increased by 14.2-fold in A549 cells and 16.2-fold in PC-3 cells, resulting in IC_50,light_ values of 33.4 nM and 72.3 nM, respectively. The anticancer activity of **Ir-H** also increased after light exposure by 10.5-fold in A549 cells and 3.49-fold in PC-3 cells, with IC_50,light_ values decreasing to the low micromolar range. It should be noted that **Ir-H** and **Ir-Me** displayed similar IC_50_ values in MRC-5 fibroblasts as in cancer cells, both in the dark and upon irradiation, evidencing their nondiscriminatory effects between nonmalignant and cancer cells.

Regarding the Ru(II) complexes, **Ru-H** and **Ru-Me** showed very moderate anticancer activity in dark conditions, which was not significantly enhanced upon exposure to blue light despite having higher light absorption at 460 nm than the Ir (III) complexes (Figure 2). Moreover, they exhibited similar toxicity levels against MRC-5 fibroblasts, as observed with the Ir(III) complexes.

The cytotoxic characterization of **Ir-H**, **Ir-Me**, **Ru**-**H**, and **Ru-Me** was further complemented by hemolysis assays, which determined whether they could induce the rupture of red blood cell membranes, leading to the release of hemoglobin.^54^ Both Ir(III) and Ru(II) complexes exhibited hemolysis levels of less than 1% both in the absence and in the presence of blue light irradiation. This finding indicates that the complexes are nontoxic to red blood cells and have good blood compatibility for future clinical applications.

Finally, the photocytotoxic activity of the **BN3**-derived metallopeptides, **Ir-BN** and **Ru-BN**, was evaluated. In PC-3 cells, **Ir-BN** exhibited similar activity relative to the precursor complex, **Ir-H**, both in the dark and upon activation with blue light. However, in MRC-5 fibroblasts, the IC_50,dark_ and IC_50,light_ values for **Ir-BN** were 2.1- and 4.4-fold higher, respectively, than those of **Ir-H** (Table 3). It should be noted that **Ir-BN** exhibited 2.3-fold and 1.9-fold higher cytotoxicity in PC-3 cells compared to MRC-5 fibroblasts and A549 cancer cells with low GRPR expression, respectively. After exposure to blue light, the selectivity for PC-3 cells increased to 3.4-fold with respect to MRC-5 fibroblasts. Regarding **Ru-BN**, the IC_50,dark_ and IC_50,light_ values in PC-3 cells were 1.7 and 8.5 times lower, respectively, than those of the precursor **Ru-H** complex. In terms of selectivity, the activity of **Ru-BN** in PC-3 cells was 1.2- and 5.7-fold higher than in A549 cells under dark and irradiated conditions, respectively. Importantly, **Ru-BN** exhibited no cytotoxicity in MRC-5 fibroblasts even at concentrations as high as 100 μM, in both dark and irradiated conditions.

The photocytotoxic effects of **Ru-H**, **Ru-Me**, and **Ru-BN** were also evaluated following photoactivation with red light (λ_ir_ = 655 nm), prompted by the red-shifted band observed in the absorption spectra of the Ru (II) complexes (Figure 2). Notably, red light offers superior tissue penetration compared to blue light, potentially enabling treatment of deeper tumors.^55^ As shown in Table 4, red light had a low impact on the activity of both Ru(II) complexes, with PIs below 1.3 in both PC-3 and MRC-5 cells. In contrast, **Ru-BN** displayed markedly higher photocytotoxic activity against PC-3 cells, with a PI of 7.3, while it displayed no cytotoxicity toward MRC-5 fibroblasts.

**Table 4:** Photocytotoxic Effects of **Ru-H**, **Ru-Me**, and **Ru-BN** after Red Light Irradiation^a^

	PC-3		MRC-5	
	IC_50,red__light_ (μM)	PI	IC_50,red__light_ (μM)	PI
**Ru-H**	54.3 ± 4.7	1.3	62.3 ± 7.1	0.9
**Ru-Me**	63.1 ± 5.8	1.2	66.6 ± 4.8	0.9
**Ru-BN**	5.66 ± 1.2	7.3	>100	--

a Cells were incubated with the compounds for 4 h at 37 °C and then kept in the dark or exposed to red light irradiation for 1 h (655 nm, 24.1 J cm^–2^). Cell viability was assessed after 48 h of treatment by MTT assays. Data represent the mean ± SD of at least three independent experiments, each performed in triplicate. PI: photocytotoxic index = IC_50, dark_/IC_50,red light_.

To examine the role of **BN3** as a carrier peptide for specifically targeting PC-3 cells, the influence of blocking the bombesin receptors on the anticancer efficacy of the metallopeptides was assessed. PC-3 cells were treated with **Ir-BN** and **Ru-BN** at concentrations near their IC_50,light_ (10 and 5 μM, respectively) in the presence of increasing bombesin concentrations (0, 10, 50, and 100 μM) to competitively inhibit GRPR binding. Following irradiation with blue light, the viability of the cells was evaluated. The photocytotoxic activity of both **Ir-BN** and **Ru-BN** was significantly attenuated in a bombesin concentration-dependent manner, with approximately a 25% reduction in the presence of bombesin at 10 μM and a more pronounced inhibition of approximately 35% at 50 and 100 μM (Figure S54). These results strongly suggest that the cytotoxic effect of **Ir-BN** and **Ru-BN** is mediated, at least in part, by their interaction with GRPR.

Overall, these findings support the efficacy of **BN3** conjugation in enhancing the selectivity of PSs toward cancer cells overexpressing GRPR. This is also evidenced by photoselectivity indexes (IC_50,dark_ in nonmalignant cells/IC_50,light_ in GRPR overexpressing cancer cells) of 9.5 for **Ir-BN** after activation with blue light and exceeding 24.3 and 17.6 for **Ru-BN** after activation with blue and red light, respectively.

### Inhibition of Colony Formation

**Figure 8:**
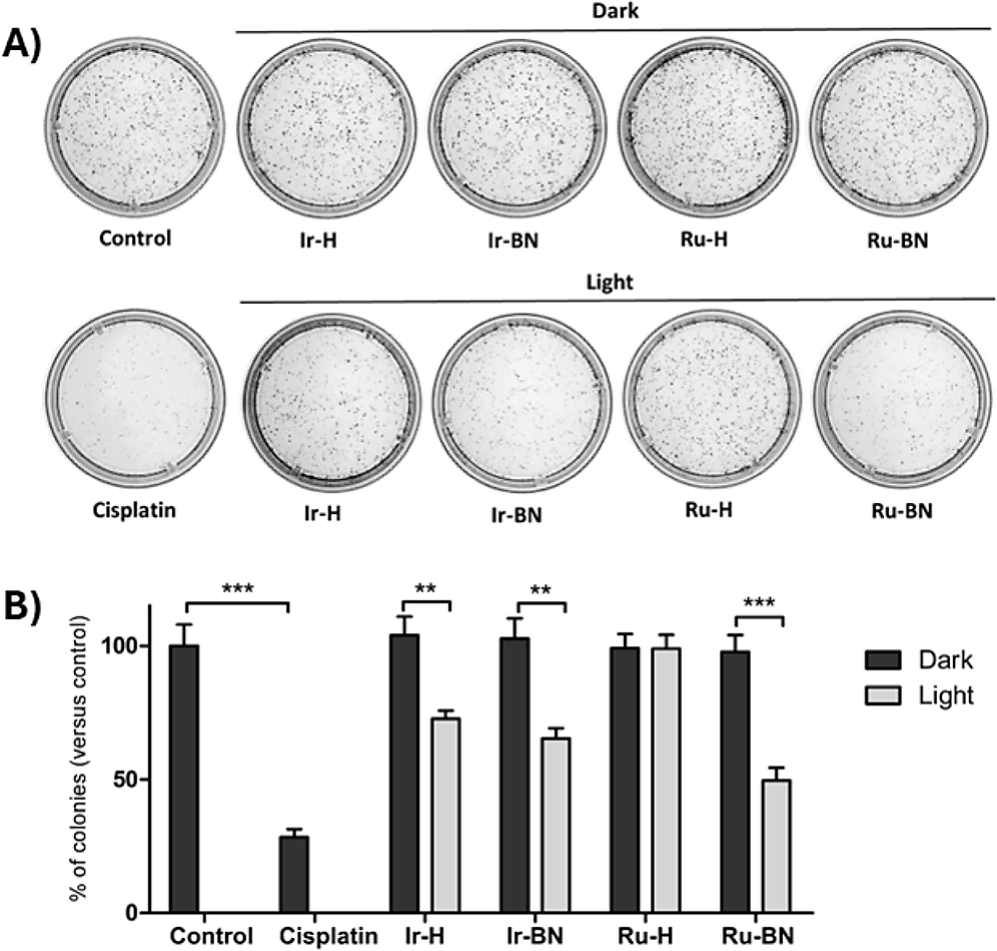
Clonogenic assay. PC-3 cells were treated with the indicated compounds at 5 μM in the dark and with blue light irradiation. Control cells were treated with the medium alone. Cisplatin was used as a positive control. Cells were incubated for 10 days to allow colony formation. (A) Images of the colonies. (B) Bar charts representing the percentage of colonies versus control cells after each treatment (mean ± SD of 3 experiments). ** *p* < 0.01; *** *p* < 0.001 versus control cells.

The effect of the metallopeptides **Ir-BN** and **Ru-BN** as well as of the complexes **Ir-H** and **Ru-H** on the viability of PC-3 cells was next investigated through clonogenic assays, which determine the fraction of cells that survive treatment and retain the ability to generate new colonies.^56^ This is a crucial feature of metastatic cancer cells, which need to proliferate in distant tissues to create secondary tumors. For this purpose, PC-3 cells were treated with the compounds at an equimolar concentration of 5 μM with or without photoactivation with blue light. Ten days later, the number of cells that survived and were able to grow and form colonies was assessed in comparison to control cells. Cells treated with cisplatin were used as a positive control. None of the compounds inhibited the clonogenic activity of the cells in the absence of light irradiation (Figure 8). However, upon blue light irradiation, the number of colonies decreased by 27.3%, 34.7%, and 50.4% in cells exposed to **Ir-H**, **Ir-BN**, and **Ru-BN**, respectively. No effect of **Ru-H** on the clonogenic capability of the cells was observed at 5 μM, which is consistent with its high IC_50,light_ value (Table 3).

### Intracellular ROS Generation

**Figure 9:**
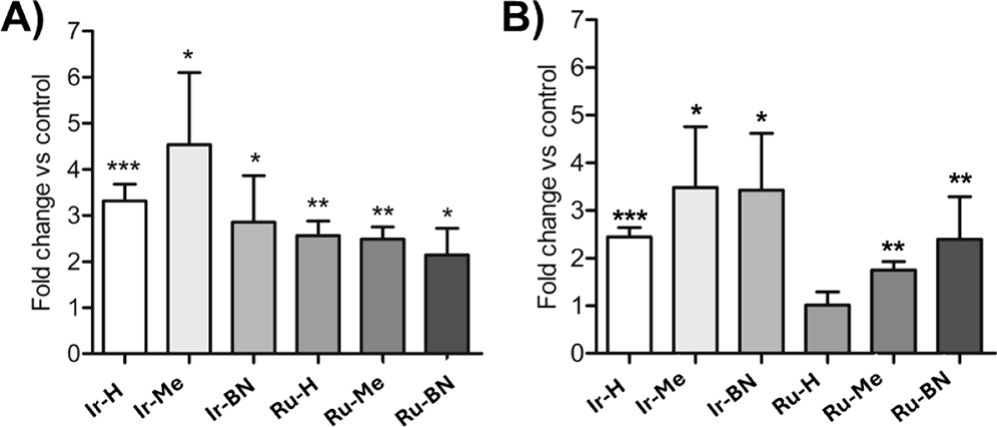
Intracellular ROS generation. PC-3 cells were treated with the indicated complexes and metallopeptides at their respective IC_50,light_ for 4 h, followed by blue light irradiation for 1 h (460 nm, 24.1 J cm^–2^). The elevation of general ROS (A) and superoxide anion (B) levels was determined with specific probes by flow cytometry. Bars represent the mean fold increase (±standard deviation) relative to control untreated cells from three independent experiments. * *p* < 0.05; ** *p* < 0.01, *** *p* < 0.001 compared to control cells.

**Figure 10:**
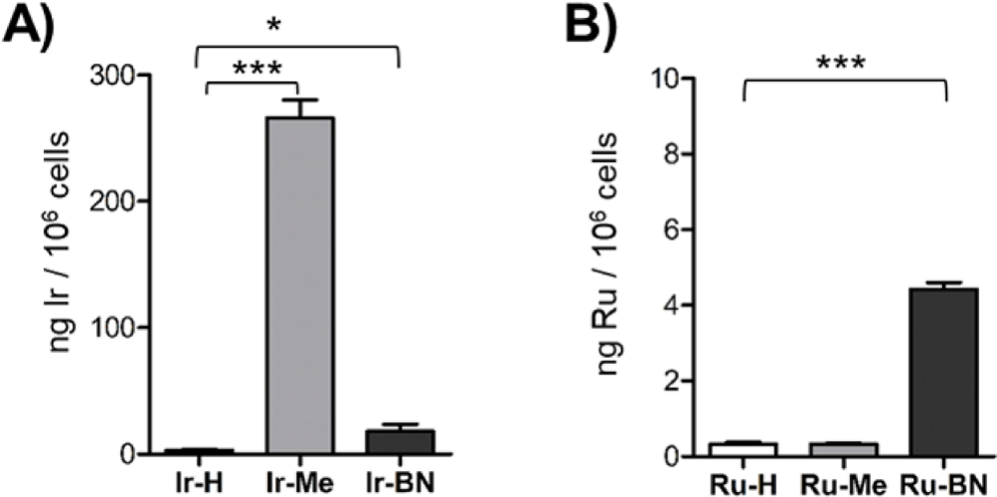
Cellular internalization. PC-3 cells were incubated for 4 h with the indicated compounds at 5 μM, and the Ir (A) or Ru (B) content per 1 × 10^6^ cells was quantified by ICP-MS. Bars represent the mean value of three replicates ± SD. * *p* < 0.05; *** *p* < 0.001 versus **Ir-H** or **Ru-H**.

The photocytotoxic effects of the complexes and metallopeptides were further elucidated by evaluating their ability to photogenerate ROS at the cellular level. PC-3 cells were treated with the compounds at the respective IC_50,light_ and irradiated with blue light. Subsequently, ROS generation was measured using the 2′,7′-dichlorodihydrofluorescein diacetate (H_2_DCFDA) probe, which diffuses into cells and is oxidized by ROS to produce a green fluorescent signal. As illustrated in Figure 9A, in all cases, ROS levels were significantly increased by more than 2-fold in comparison to the control cells. **Ir-Me** exhibited the most potent ROS-generating activity, with a 4.5-fold increase. It is worth noting that, despite **Ir-Me** exhibiting relatively low efficiency as a photocatalyst for the generation of ^1^O_2_, its effective cellular uptake could counterbalance this limitation (see Figure 10), enabling effective ROS generation inside the cells. Nevertheless, to ascertain the potential contributions of other ROS to the photocytotoxic activity, the capacity of the compounds to undergo type I PDT processes, resulting in the production of superoxide anions (O_2_^•–^), was examined using a specific fluorescent probe that emits an orange signal upon interaction with this radical. **Ir-H**, **Ir-Me**, and **Ir-BN** induced the most significant increase in O_2_^•–^ levels, with fold changes of 2.4, 3.5, and 3.4, respectively, compared to control cells. In contrast, the Ru complexes exhibited lower increments (Figure 9B). These findings highlight the diverse mechanisms of ROS generation employed by these compounds, in particular the Ir complexes, which may contribute to their overall photocytotoxic efficacy.

### Internalization Studies

The intracellular accumulation of the PSs is a crucial requirement to achieve a photocytotoxic effect. This is because the ROS generated by the PSs have a very limited radius of action and only operate in the cellular compartment or organelle in which they are generated.^57^ In particular, the absence of hemolytic activity observed in both Ir(III) and Ru(II) complexes suggests a mechanism of action that does not involve cell membrane disruption. This further emphasizes the importance of cellular uptake for their cytotoxic activity. Additionally, when using tumor-targeting ligands, such as **BN3**, the intracellular uptake of the metallopeptides depends on the level of expression of the target receptor on the cell surface and the internalization mechanism, which may differ from that of the free complex. Therefore, the internalization of complexes **Ir-H**, **Ir-Me**, **Ru**-**H**, and **Ru-Me** and of metallopeptides **Ir-BN** and **Ru-BN** in PC-3 cells was determined by quantifying the intracellular metal content by inductively coupled plasma mass spectrometry (ICP-MS) (Figure 10). After 4 h treatment with the compounds at 5 μM, the amount of Ir in cells exposed to **Ir-H** was 3.1 ± 0.7 ng per million cells, whereas in the case of its conjugated form **Ir-BN**, it increased 5.7-fold to 17.9 ± 5.6 ng per million cells. This result showed that conjugation to **BN3** enhances the cellular uptake of the Ir(III) complex. Notably, **Ir-Me** exhibited the highest internalization, resulting in 266.4 ± 13.9 ng Ir per million cells, consistent with its superior anticancer activity. We speculate that the higher ability of **Ir-Me** to accumulate within the cells is consistent with its lipophilic character (vide infra). By contrast, **Ir-H** exhibited a poor intracellular uptake likely due to the presence of the negatively charged −COO^–^ group in its deprotonated zwitterionic form. It should be noted that **Ir-Me** has a significantly higher internalization capacity than the **Ir-BN** conjugate. This discrepancy can be attributed to the lipophilic nature of **Ir-Me**, which enables its passive diffusion across the cell membrane. Consequently, **Ir-Me** can be more rapidly and directly internalized by cells. Conversely, **Ir-BN** entry involves receptor-mediated endocytosis, a slower, energy-dependent process that often results in lower intracellular concentrations and can be influenced by variations in the endocytic pathway activity.

In the case of cells treated with the Ru(II) complexes, the intracellular metal contents were markedly lower, with 0.329 ng per million cells and 0.326 ng per million for **Ru-H** and **Ru-Me**, respectively. We believe that the scarce accumulation of the Ru derivatives is due to their lower lipophilicity (vide infra), since in the physiological pH range, **Ru-H** shows a monocationic zwitterionic form and **Ru-Me** a dicationic nature. These charged states may hinder passive diffusion across the cell membrane. However, the amount of intracellular Ru significantly increased 14-fold to 4.46 ng/million cells for **Ru-BN**.

Overall, these results demonstrate that the conjugation of the complexes to **BN3** not only enhances their selectivity for tumor cells but also increases the intracellular accumulation of the conjugated complexes **Ir-H** and **Ru-H**, leading to increased cytotoxic activity, particularly for the Ru(II) derivative.

### Lipophilicity and Self-Aggregation Studies

In order to obtain a better understanding of the cellular uptake abilities of these complexes, we carried out experiments to determine their lipophilicity and self-aggregation properties.

First, the *n*-octanol/PBS partition coefficient values, log P_oct/PBS_, of the metal PSs were experimentally determined to quantify their lipophilicity (Figure S56). Indeed, the shake flask method was employed, and the metal content was measured in both phases by UV/vis spectroscopy. Thus, we established that both Ru derivatives exhibited low lipophilicity in agreement with either the dicationic nature of **Ru-Me** (log P_oct/PBS_ = −0.43) or the monocationic zwitterionic character of **Ru-H** (log P_oct/PBS_ = −1.10) at physiological pH. These results are consistent with the low cellular uptake shown by these derivatives.

By contrast, both Ir derivatives show higher lipophilicity relative to their Ru congeners, which is rationally explained as a result of the monocationic nature of **Ir-Me** and the neutral zwitterionic character of **Ir-H** at physicological pH. Indeed, no significant differences can be observed in the lipophilicity between **Ir-Me** (log P_oct/PBS_ = 2.03) and **Ir-H** (log P_oct/PBS_ = 1.80). Therefore, we speculate that the pronounced divergent cellular uptake behavior experimentally observed for **Ir-H** and **Ir-Me** could be explained as a result of the electronic repulsion between the negatively charged −COO^–^ group in **Ir-H** and the negative charge of the phosphate groups present in the cellular membrane. In other words, we assume that the presence of the −COO^–^ group in the zwitterionic form of **Ir-H** at physiological pH values would hinder its uptake through the cell membrane by passive diffusion.^58^

The possible aggregation properties (self-assembly behavior) were investigated for the lipophilic derivative **Ir-Me** through the respective analysis of the Lambert–Beer law using UV–vis spectroscopy in H_2_O:DMSO (99:1). More specifically, the absorbance of **Ir-Me** at λ = 417 nm was plotted versus concentration in the range between 1 and 40 μM. An excellent linear fitting was obtained confirming that there is no significant deviation from the Lambert–Beer law (Figures S57 and S58). Therefore, we conclude that **Ir-Me** does not form aggregates under these conditions.

### Intracellular Distribution

The intracellular distribution of the compounds was then investigated by confocal microscopy. Preliminary flow cytometry experiments revealed that significant intracellular fluorescence was detectable only in cells exposed to **Ir-Me**, consistent with the higher intracellular accumulation of this compound (Figure S55).

**Figure 11:**
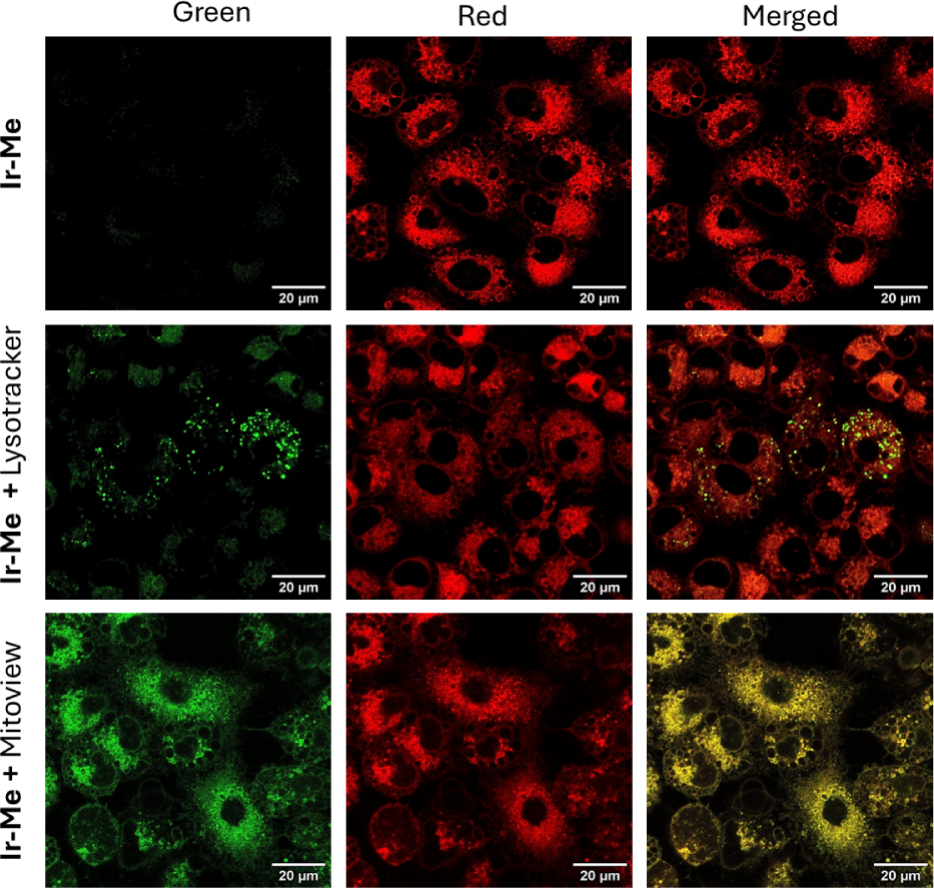
Confocal images of A549 cells after 1 h of incubation with **Ir-Me** at 1 μM (red). MitoView Green or LysoTracker Green DND-26 (green) were used for colocalization studies with mitochondria or lysosomes, respectively. Colocalization is shown in yellow in the merged image. Scale bars represent 20 μm.

Accordingly, confocal microscopy images showed a strong red fluorescence signal from **Ir-Me** inside the cells, as shown in Figure 11. The complex displayed a high degree of colocalization with the mitochondrial dye MitoView Green, as evidenced by the yellow signal in the merged image (Pearson correlation coefficient (PCC): 0.832). In contrast, a lower degree of overlap was observed with the LysoTracker Green DND-26 lysosomal dye (PCC: 0.599), indicating that the complex predominantly accumulates within the mitochondria and exhibits a lower distribution within the endolysosomal system. This is compatible with the monocationic and lipophilic nature of **Ir-Me** and has been previously reported for other mitochondria-targeting monocationic bis-cyclometalated Ir(III) complexes.^59−63^

### Mitochondrial Targeted Activity

**Figure 12:**
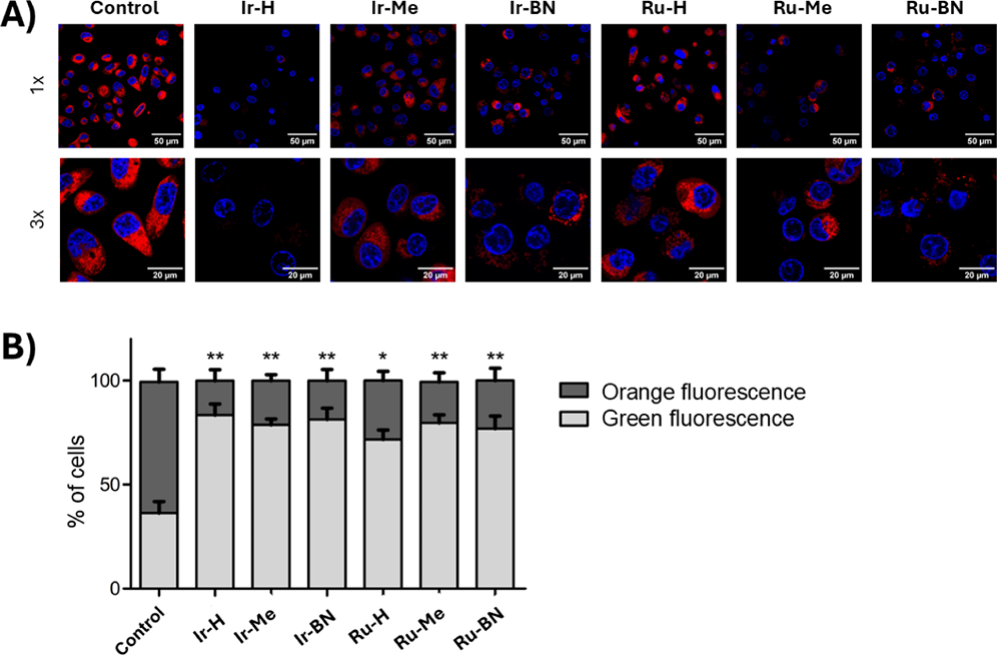
Effect on mitochondria function. PC-3 cells were incubated with the indicated compounds at the corresponding IC_50,light_ for 4 h at 37 °C and then exposed to blue light for 1 h. Cells incubated with the medium alone served as the negative control. (A) Confocal microscopy images of the cells. Nuclei were stained blue with Hoechst (λ_ex_: 350 nm; λ_em_: 461 nm), and mitochondria were labeled with MitoTracker Red CMXRos (λ_ex_: 579 nm; λ_em_: 599 nm). Insets show a 3× magnification of the cells. Scale bars represent 50 μm in 1× images and 20 μm in 3× images. (B) Flow cytometry analysis of the effect of the complexes on mitochondrial membrane potential. The percentage of cells exhibiting JC-10 green (λ_em_: 529 nm) and orange fluorescence (λ_em_: 590 nm) is represented (mean ± SD of three independent experiments). A decrease in the percentage of cells emitting orange fluorescence indicates a loss of MMP. *p* < 0.05 and ** *p* < 0.01 compared to control cells.

Given the high degree of accumulation of **Ir-Me** in mitochondria, the potential of complexes **Ir-H**, **Ir-Me**, **Ru**-**H**, and **Ru-Me** and of metallopeptides **Ir-Bn** and **Ru-BN** to induce mitochondrial dysfunction was next investigated. Mitochondria are the primary source of ATP and anabolites for cellular metabolism and are involved in the regulation of key cellular functions, including redox status and cell signaling. Consequently, they play a crucial role in both cell growth and cell death regulation.^64^ PC-3 cells were incubated with the compounds at the corresponding IC_50,light_ for 4 h and subsequently exposed to blue light irradiation for 1 h. Subsequently, mitochondria were labeled with MitoTracker Red CMXRos, a lipophilic cationic red fluorescent dye that accumulates within healthy mitochondria due to their negative mitochondrial membrane potential (MMP).^65^ Cell nuclei were counterstained in blue using Hoechst 33342 to localize the cells. Confocal microscopy images revealed a strong red fluorescence emission from mitochondria in untreated control cells (Figure 12A). Conversely, exposure of cells to the photoactivated complexes and metallopeptides led to a notable attenuation of the mitochondrial red fluorescence, which was particularly evident in the case of the complex **Ir-H** and of the metallopeptides **Ir-BN** and **Ru-BN**. These results strongly suggest that these complexes exert their photodynamic activity by inducing mitochondrial membrane depolarization, a hallmark of mitochondrial dysfunction.^65^

Flow cytometry analysis using the mitochondrial specific dye JC-10 confirmed this effect. The fluorescence of JC-10 undergoes a reversible change from green to orange as MMP increases, due to the formation of JC-10 aggregates within polarized mitochondria. This property allows for the simultaneous detection of healthy and depolarized mitochondria. In control cells, orange fluorescence emission corresponding to healthy mitochondria was detected in 63.0 ± 10.6% of cells (Figure 12B). However, treatment with the photoactivated compounds significantly reduced the population of cells emitting orange fluorescence to 16.6 ± 9.2%, 21.2 ± 4.9%, and 16.0 ± 9.5% for **Ir-H**, **Ir-Me**, and **Ir-BN**, respectively, and to 28.2 ± 7.8%, 19.7 ± 7.6%, and 23.0 ± 10.4% for **Ru-H**, **Ru-Me**, and **Ru-BN**, respectively (Figure 12B), confirming mitochondrial membrane depolarization. Consistent with the microscopy experiments, the most notable effect on MMP was observed with complex **Ir-H**. **Ir-BN** and **Ru-BN** metallopeptides also induced significant mitochondrial membrane depolarization, indicating that conjugation with **BN3** does not hinder this effect. Furthermore, the complexes’ ability to photocatalytically oxidize NADH, a primary electron donor for the mitochondrial electron transport chain, may contribute to the mitochondrial damage.

### Cell Death Mechanism

**Figure 13:**
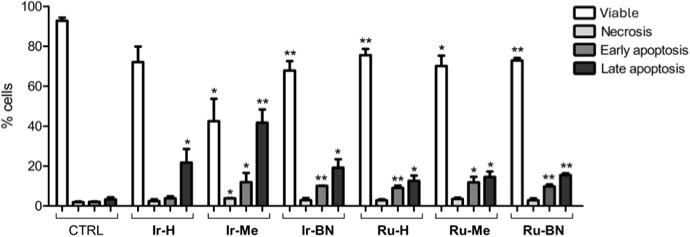
Cell death mechanism. PC-3 cells were treated with the photoactivated compounds at five times the corresponding IC_50,light_. After 24 h, the percentage of viable, necrotic, early apoptotic, and late apoptotic cells was analyzed by flow cytometry using propidium iodide and annexin V staining. Untreated cells were used as controls. Bars represent the mean ± standard deviation from three independent experiments. *p < 0.05 and **p < 0.01 versus control cells.

Mitochondria play an important role in the regulation of apoptosis. Alterations in the MMP can cause the release of apoptotic factors, such as cytochrome c, which activates the caspase cascade, leading to programmed cell death.^66,67^ In order to elucidate whether the toxic effect of the compounds at the mitochondrial level triggers cell death by apoptosis, a dual annexin V/propidium iodide labeling experiment was performed. The initial stages of apoptosis are characterized by alterations in the symmetry of phospholipids in the cytoplasmic membrane, which can be detected using annexin V (An). The late stages also include disruption of the cell membrane, permitting the penetration of propidium iodide (PrI), which emits red fluorescence when bound to DNA. In contrast, the cell membrane of necrotic cells becomes readily permeable to PrI but does not exhibit phospholipid translocations. This allows the discrimination among viable cells (An–/PrI−), necrotic cells (An–/PrI+), and early-(An+/PrI−) and late-stage (An+/PrI+) apoptotic cells. PC-3 cells were treated with the photoactivated complexes and metallopeptides at five times the corresponding IC_50,light_, and 24 h later, samples were analyzed using flow cytometry. In all cases, the treatment resulted in a significant increase in the number of apoptotic cells, particularly in the late stage of apoptosis, compared to control cells (Figure 13). A higher percentage of apoptotic cells was detected upon treatment with Ir(III) complexes than Ru(II) complexes, reaching a 11.9% of cells in early apoptosis and 41.7% of cells in late apoptosis in the case of **Ir-Me**. In contrast, the population of necrotic cells was not increased by any treatment. Overall, these results indicated that the photodynamic activity of both the complexes and metallopeptides promotes a regulated cell death by apoptosis.

## Conclusions

In conclusion, we have proved that the conjugation of Ru(II)- or Ir(III)-based PSs to the bombesin derivative **BN3** can be employed successfully to develop efficient PDT agents with two possible levels of selectivity in their anticancer action: (1) the first level would be provided by the targeting ability of **BN3** toward cancer cells that overexpress BN receptors; (2) the second level is based on the local activation of the metal fragments of our PSs upon photoirradiation of the tumors.

Our Ru(II) and Ir(III) complexes were designed to combine remarkable photocatalytic properties in the generation of ROS and suitable functional groups on the N^N’ ligand for either enable peptide conjugation (−COOH) or facilitate the cellular internalization (−COOMe). Thus, following straightforward synthetic approaches, we have demonstrated that it is possible to obtain the complexes **Ru-H**, **Ru-Me**, **Ir**-**H**, and **Ir-Me** and their respective metallopeptides **Ru-BN** and **Ir-BN**. Moreover, we have shown that the free complexes absorb visible light efficiently and that they are phosphorescent, although with low emission quantum yields. We also established that all the metal complexes are photostable and that **Ru-H** and **Ir-H** adopt their deprotonated zwitterionic forms in the whole range of physiological pHs.

Regarding their biological properties, our findings indicate that Ir(III) PSs exhibit greater cytotoxicity than their Ru(II) congeners in the dark against A549 and PC-3 cancer cells but also against nonmalignant fibroblasts. In particular, **Ir-Me** was found to exhibit the highest anticancer potency. Upon blue light irradiation, the cytotoxicity of both Ir(III) PSs was significantly enhanced, with **Ir-Me** reaching PI values above 14 in cancer cells. Consistently, internalization studies demonstrated that **Ir-Me** accumulated more efficiently than **Ir-H** in the cells. Overall, these results revealed that esterification with a methyl group avoids the formation of the carboxylate group (−COO^–^), increasing the cellular internalization of the monocationic **Ir-Me** complex, and therefore its cytotoxicity, relative to its zwitterionic congener, **Ir-H**. The Ru(II) PSs, **Ru-H** and **Ru-Me**, exhibited low cytotoxicity both in the dark and upon photoactivation, which is consistent with their low lipophilicity and low cellular internalization, with minor differences between them.

We also disclosed that binding of **Ir-H** to **BN3** notably enhances its selectivity toward cancer cells overexpressing GRPR, as reflected by the lower IC_50_ values of the resulting metallopeptide **Ir-BN** in PC-3 cells compared to A549 cells, which exhibit low GRPR expression, and importantly, to noncancerous MRC-5 fibroblasts. Furthermore, conjugation of **Ru-H** to **BN3** has been demonstrated to markedly enhance the accumulation and photocytotoxic efficacy of the resulting metallopeptide **Ru-BN i**n PC-3 cancer cells, while exerting no toxicity on noncancerous fibroblasts. The attenuation of the photocytotoxic activity of **Ir-BN** and **Ru-RN** in the presence of bombesin supports that the activity of the metallopeptides is at least partially mediated by binding to GRPR. Hence, these results prove that conjugation of the metal PSs to **BN3** as tumor-targeting peptide is a promising strategy in terms of selectivity and photocytotoxic activity.

Clonogenic assays confirmed the light-dependent cytotoxic activity of **Ir-H**, **Ir-BN**, **Ru-H**, and **Ru-BN**. Following blue light irradiation, the colony-forming ability of treated cells was significantly reduced, indicating effective photocytotoxicity. Hemolysis assays demonstrated that the complexes are nontoxic to red blood cells, suggesting good blood biocompatibility. These experiments also indicated that they do not cause damage to cell membranes. Regarding the mechanism of action, experimental evidence indicates that the complexes accumulate within mitochondria, where they can generate ^1^O_2_ and O_2_^•–^ and oxidize NADH in a photocatalytic manner. This subsequently causes depolarization of the mitochondrial membrane and the initiation of apoptosis.

Overall, this study validates the potential of exploiting specific alterations in human cancer cells, such as overexpression of the bombesin receptor, to design novel PDT agents with superior selectivity and potency. Our findings indicate that **BN3** could effectively deliver metal-based PSs to tumor tissues, enhancing their accumulation and minimizing off-target effects. This targeted approach holds significant promise for the development of more effective and selective PDT strategies.

## Experimental Procedures

### General Information

All synthetic manipulations for Ir(III) and Ru(II) complexes were carried out under an atmosphere of dry, oxygen-free nitrogen using standard Schlenk techniques. The solvents were dried and distilled under a nitrogen atmosphere before use. Elemental analyses were performed with a Thermo Fisher Scientific EA Flash 2000 Elemental Microanalyzer. UV–vis absorption was measured in a Jasco V-750 UV–visible spectrophotometer. Fluorescence steady-state and lifetime measurements were performed in an FLS980 (Edinburgh Instruments) fluorimeter with Xenon Arc Lamp 450 W and TCSPC laser, respectively. Photoluminescence quantum yields was determined by using FLS980 (Edinburgh Instruments) with Xenon Arc Lamp 450 W and Red PMT Sphere as a detector. HR-ESI(+) mass spectra were recorded with an Agilent LC-MS system (1260 Infinity LC/6545 Q-TOF MS spectrometer) using dichloromethane (DCM) as a sample solvent and H_2_O (0.1% formic acid)/MeOH (0.1% formic acid) and 30:70 as the mobile phase. The experimental *m*/*z* values are expressed in Da and were compared with the *m*/*z* values for monoisotopic fragments. NMR spectra were recorded at 298 K on Bruker Avance III (300.130 MHz for ^1^H; 75.468 MHz for ^13^C). ^1^H NMR spectra were acquired with 32 scans into 32 k data points over a spectral width of 16 ppm. ^1^H and ^13^C{^1^H} chemical shifts were internally referenced to TMS via the residual ^1^H and ^13^C signals of DMSO-*d*_6_ (δ = 2.50 ppm and δ = 39.52 ppm), according to the values reported by Fulmer et al.^68^ Chemical shift values (δ) are reported in ppm and coupling constants (J) in hertz. The splitting of proton resonances in the reported ^1^H NMR data is defined as s = singlet, d = doublet, t = triplet, q = quartet, m = multiplet, and bs = broad singlet. 2D NMR spectra were recorded using standard pulse sequences. All NMR data processing was carried out using MestReNova version 10.0.2.

### Cell Culture

The PC-3 human prostate cancer, A549 basal lung adenocarcinoma, and MRC-5 lung fibroblast cell lines were obtained from the American Type Culture Collection (ATCC). Cells were cultured in Dulbecco’s modified Eagle’s medium (DMEM) (Corning) supplemented with 10% fetal bovine serum (FBS) (Gibco-BRL) and 1% l-glutamine-penicillin-streptomycin (Cultek). Cells were maintained in a Heracell 150 incubator (Thermofisher Scientific) at 37 °C in a 5% CO_2_ atmosphere. Mycoplasma contamination was regularly monitored with the Mycoplasma Gel Detection Kit (Biotools).

### Photocytotoxic Activity

Stock solutions of the complexes and metallopeptides were initially prepared at a concentration of 5 mM in DMSO and then diluted in sterile distilled water to achieve a final concentration of 1 mM, with a resulting DMSO concentration of 20% v/v. Cells were seeded into 96-well plates at a density of 2500 cells/well for A549, 3500 cells/well for PC-3, and 5500 cells/well for MRC-5. Following 24 h of incubation for attachment, cells were treated in triplicate with freshly prepared working solutions of the compounds in the culture medium at concentrations ranging from 0.001 to 100 μM. The maximum DMSO concentration in the working solutions was 2% (v/v). The working concentrations for each compound were adjusted based on their cytotoxicity. Following a 4 h incubation period to allow internalization of the compounds into the cells, the plates were kept in dark conditions or exposed to blue (460 nm) or red (655 nm) light for 1 h using an LED system (LuxLight), providing a total dose of 24.1 J cm^–2^. All plates were incubated in the dark for an additional 43 h. Cells were then washed with phosphate-buffered saline (PBS), and 100 μL of fresh culture medium containing 10% of 3-[4,5-dimethylthiazol-2-yl]-2,5 diphenyl tetrazolium bromide (MTT) solution (0.5 mg/mL) (Sigma- Aldrich) was added to each well. After a 2 h incubation period, the formazan crystals were dissolved in DMSO, and the absorbance was measured at 570 nm using a Multiscan Plate Reader (Synergy 4, Biotek, Winooski, USA). The concentration causing a 50% reduction in cell viability (IC_50_) was determined for each compound with the Gen5 software (BioTek). At least three independent experiments were conducted for each compound.

For bombesin receptor blocking experiments, PC-3 cells seeded in 96-well plates were treated with bombesin (Thermo Scientific) at increasing concentrations (0, 10, 50, and 100 μM) for 30 min, followed by the addition of **Ir-BN** or **Ru-BN** at final concentrations of 10 or 5 μM, respectively. For each bombesin concentration, control cells without metallopeptide treatment were included to account for a possible effect of bombesin on cell proliferation. After 4 h of incubation, the treatments were removed, and the cells were exposed to blue light for 1 h. 43 h later, MTT assays were carried out. The effects of the treatments were calculated by comparing the absorbance of treated cells with that of cells exposed to the same concentration of bombesin without metallopeptides. Each treatment was performed in duplicate, and two independent experiments were performed.

### Clonogenic Assay

PC-3 cells were seeded at a density of 100000 cells per well in 12-well plates and allowed to adhere for 24 h. The cells were treated with the compounds at their respective IC_50,light_ for 4 h and subsequently irradiated with blue light or kept in the dark for one hour. The treatments were then removed, and the cells were washed with PBS, harvested by trypsinization, and counted using a Novocyte flow cytometer (Agilent Technologies). Subsequently, 3000 cells were seeded in 5 cm culture dishes and incubated for 10 days to allow colonies to form. A colony was defined to consist of at least 50 cells. Cells treated with cisplatin (5 μM) were used as a positive control, and cells treated with the culture medium alone were used as a negative control. Dishes were washed with PBS, and cells were fixed and stained with 1% methylene blue in 70% ethanol. Images of the dishes were obtained with the Alpha Innotech Imaging System (Alpha Innotech), and colony counting was performed using Fiji ImageJ software. Each compound was evaluated in triplicate.

### Hemolysis Assay

Hemolysis assays were performed using commercially available porcine blood preserved in sodium polyphosphate as an anticoagulant (Norfrisa, Spain). Blood was diluted with PBS to a concentration of 5%, and red blood cells (RBCs) were obtained by centrifugation. Subsequently, 150 μL of the RBC suspension was incubated with 150 μL of each compound at its respective IC_50,light_ for 4 h, followed by 1 h of incubation under blue light irradiation or in the dark with agitation on an orbital shaker. Samples treated with PBS were used as negative controls, and a solution of PBS with 0.2% Tween was used as a positive control to induce 100% RBC lysis. All treatments were performed in duplicates. The samples were then centrifuged, and 80 μL of the supernatant was diluted with an equal volume of water and added to a 96-well plate. Hemoglobin release in the supernatant was measured at 540 nm using a Synergy 4 plate reader (Biotek). The optical density (OD) values obtained from samples treated with the compounds (ODtest) were normalized relative to the positive (ODpos) and negative (ODneg) control samples to obtain the hemolysis ratio (HR) using the following equation:
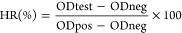


### ROS Generation

PC-3 cells were seeded at a density of 100000 cells per well in 12-well plates and incubated overnight. The compounds were added to the cells at their respective IC_50,light_ and incubated for 4 h to allow internalization. The treatments were removed, and after washing with PBS, the 2′,7′-dichlorodihydrofluorescein diacetate (H_2_DCFDA) probe (Sigma-Aldrich) at 10 μM was added to each well. Cells were then irradiated with blue light (24.1 J cm^–2^) or kept in the dark for 1 h. Cells were subsequently harvested by trypsinization, and the median fluorescence emission of 10000 cells was measured using a Novocyte flow cytometer equipped with NovoExpress software. The fluorescence fold increase relative to untreated control cells was calculated. Each experiment was performed in triplicate for each complex and cell line.

A similar protocol was followed to evaluate superoxide production using the ROS-ID Superoxide Detection Kit (Enzo Life Sciences) according to the manufacturer’s instructions.

### Cellular Internalization

PC-3 cells were seeded in 6-well plates at a density of 2 million cells/well. After 24 h, cells were incubated with the compounds at a concentration of 5 μM for 4 h. Untreated cells were used as a negative control. Cells were washed with PBS and harvested by trypsinization. The number of cells in each sample was determined using a Novocyte flow cytometer (Agilent Technologies). The samples were centrifuged to obtain the cell pellet, and the complex content was subsequently assessed using inductively coupled plasma mass spectrometry (ICP-MS). To this end, the cell pellets were dissolved in 400 μL of 69% v/v concentrated nitric acid and heated at 60 °C overnight. The digested samples were allowed to cool and diluted until 5 mL with Milli-Q water. The Ir or Ru content was analyzed in Agilent 7500c ICP-MS located at the Technical Research Services of the University of Girona. Standards were freshly prepared in Milli-Q water containing the same proportion of HNO_3_ (8%) before each experiment. The concentrations used for the calibration curve were approximately 0, 3, 9, 17, 35, 66, and 100 μg.kg^–1^. Rhodium was added to all samples and standards as the internal standard at a concentration around 10 μg.kg^–1^. The isotopes detected were ^193^Ir, ^101^Ru, and ^103^Rh, respectively. Measures were carried out in triplicate. ^193^Ir/^103^Rh and ^101^Ru /^103^Rh signal ratios were corrected with the real exact Rh concentration in each sample and standard. The amount of metal in each sample was normalized to the cell number. Three independent samples were analyzed for each complex.

### Confocal Microscopy

The subcellular distribution of the complexes was assessed by confocal fluorescence microscopy. A549 cells were selected for these experiments as they have an extended cytoplasm, which facilitates the visualization of different organelles. Cells were plated on glass-bottom 8-well chamber slides (Ibidi) at a density of 50000 cells per well. After 24 h, the cells were treated with **Ir-Me** at 1 μM in DMEM without phenol red. Untreated cells were used as the negative control. To assess the colocalization with specific organelles, MitoView Green (Biotium) (excitation/emission: 490/523 nm) and LysoTracker Green DND-26 (Thermo Fisher Scientific) (excitation/emission: 504/511 nm) dyes were used at a concentration of 100 nM to label mitochondria and lysosomes, respectively. After 1 h of incubation at 37 °C, cells were washed with cold PBS and immediately imaged using a Nikon A1R confocal microscope. Images were analyzed using NIS-Elements AR (Nikon, Japan) and ImageJ software. Colocalization was assessed by the Pearson correlation coefficient, using the JACoP plugin.^69^

### Mitochondrial Damage

Confocal microscopy: PC-3 cells were plated on glass-bottom 8-well chamber slides at a density of 75000 cells per well. 24 h later, the cells were treated with the compounds at their respective IC_50,light_ for 4 h, followed by exposure to blue light for 1 h or incubation in the dark. Untreated cells were used as the negative control. The cells were then rinsed three times with PBS and incubated with the MitoTracker Red CMXRos dye (Molecular Probes) (excitation/emission: 579/599 nm) at a concentration of 200 nM in phenol red-free DMEM for 30 min at 37 °C. Cell nuclei were stained blue using Hoechst 33342 dye (Invitrogen) (excitation/emission: 350/461 nm) diluted 1:4000. Images were captured using a Nikon A1R confocal microscope and analyzed using ImageJ software.

Flow cytometry assessment of the mitochondrial membrane depolarization: PC-3 cells were seeded on 12-well plates at a density of 100000 cells per well. 24 h later, cells were treated with the compounds as described above. Cells were subsequently harvested using trypsinization and incubated with JC-10 dye (Deltaclon) according to the manufacturer’s protocol. A Novocyte flow cytometer was employed to analyze the fluorescence emission of JC-10 in 10000 cells. Fluorescence was detected at 590 nm (FL2) to identify the percentage of cells with healthy mitochondria and at 529 nm (FL1) to determine the percentage of cells with depolarized mitochondria. Each compound was evaluated in three independent experiments, and the mean and standard deviation of the results were represented.

### Apoptosis Assays

PC-3 cells were seeded into 12-well plates at a density of 100000 cells/well. After 24 h, cells were treated with compounds at a concentration five times the corresponding IC_50_ under blue light irradiation. Cisplatin at 25 μM was used as the positive control. 24 h later, cells were harvested by trypsinization and stained with the Vybrant Apoptosis Assay Kit (Molecular Probes) following the manufacturer’s protocol. The samples were immediately analyzed using a Novocyte flow cytometer. Annexin-FITC staining was detected at a wavelength of 520 nm, and propidium iodide was detected at 617 nm. The fluorescence emission of 10000 cells per sample was measured, and the percentages of live, early apoptotic, late apoptotic, and necrotic cell populations were determined.

## Supporting Information Available

General information and methods, synthesis and characterization of ligands and complexes, NMR spectroscopy, high-resolution mass spectrometry, synthesis of metallopeptides **Ir-BN** and **Ru-BN**, characterization of metallopeptides **Ir-BN** and **Ru-BN**, X-ray diffraction, photostability, determination of p*K*_a_, singlet oxygen generation, photocatalytic oxidation of NADH, bombesin receptor blocking experiments, intracellular fluorescence of the compounds and lipophilicity and self-aggregation studies (PDF)

## Abbreviations

^1^O_2_: Singlet oxygen
ABDA: 9,10-anthracenediyl bis(methylene)dimalonic acid
ATCC: American Type Culture Collection
BN: Bombesin
bpy: 2,2′-bipyridine
DCM: dichloromethane
DIC: *N*,*N*-diisopropylcarbodiimide
DMEM: Dulbecco’s modified Eagle’s medium
DMF: dimethylformamide
DMSO: dimethyl sulfoxide
FBS: fetal bovine serum
FITC: fluorescein isothiocyanate
Fmoc: 9-fluorenylmethoxycarbonyl
GRP: gastrin-releasing peptide
GRP: gastrin-releasing peptide receptor
HPLC: high-performance liquid chromatography
HR ESI(+) MS: high-resolution electrospray ionization mass spectrometry
ICP-MS: inductively coupled plasma mass spectrometry
IC_50_: half maximal inhibitory concentration
LC: ligand centered
LLCT: ligand to ligand charge transfer
MFI: median fluorescence intensity
MLCT: metal to ligand charge transfer
MMP: mitochondrial membrane potential
MTT: 3-[4,5,dimethylthiazol-2-yl]-2,5-diphenyl tetrazolium bromide
NAD+: nicotinamide adenine dinucleotide (oxidized form)
NADH: nicotinamide adenine dinucleotide (reduced form)
NMR: nuclear magnetic resonance
O_2_^•^^–^: superoxide anion
OD: optical density
PBS: phosphate-buffered saline
PCC: Pearson correlation coefficient
PDT: photodynamic therapy
p*K*_a_: acid dissociation constant
PI: photocytotoxic index
PrI: propidium iodide
ppy: 2-phenylpyridine
PS: photosensitizer
RBC: red blood cells
ROS: reactive oxygen species
SD: standard deviation
TFA: trifluoroacetic acid
TIS: triisopropylsilane
tBu: *tert*-butyl
τ: excited state lifetimes
φ: photoluminescence quantum yields
UV–vis: ultraviolet–visible
λ_em_: wavelength of emission
λ_ex_: wavelength of excitation
