# Supplementary material for: Bombesin-Targeted Delivery of β-Carboline-Based Ir(III) and Ru(II) Photosensitizers for a Selective Photodynamic Therapy of Prostate Cancer
Source: Inorg Chem. 2024 Oct 3;63(41):19140–55. doi: 10.1021/acs.inorgchem.4c02583 (PMC11483813; doi:10.1021/acs.inorgchem.4c02583)
Supplement: Supplementary file 1 — ic4c02583_si_001.pdf [file ic4c02583_si_001.pdf]

SUPPORTING INFORMATION

Bombesin-targeted delivery of β-carboline-based Ir(III) and Ru(II) photosensitizers for a selective photodynamic therapy of prostate cancer

Juan Sanz-Villafruela,[a,#] Cristina Bermejo-Casadesús,[b,#] Gerard Riesco-Llach,[c] Mònica Iglesias,[d] Marta Martínez-Alonso,[a] Marta Planas,[c] Lidia Feliu,[c] Gustavo Espino,[a,*] Anna Massaguer [b,*].

[a]Universidad de Burgos, Departamento de Química, Facultad de Ciencias, Plaza Misael Bañuelos s/n, 09001, Burgos, Spain.

[b]Universitat de Girona, Departament de Biologia, Facultat de Ciències, Maria Aurelia Capmany 40, 17003 Girona, Spain.

[c] LIPPSO, Universitat de Girona, Departament de Química, Facultat de Ciències, Maria Aurelia Capmany 69, 17003 Girona, Spain.

[d] Universitat de Girona, Departament de Química, Facultat de Ciències, Maria Aurelia Capmany 69, 17003 Girona, Spain.

# Equally contributed.

* Corresponding authors:

Dr. Gustavo Espino: gespino@ubu.es, telephone: +34 947105602

Dra. Anna Massaguer: anna.massaguer@udg.edu, telephone: +34 636674635

Table of Contents

1. General information and methods 3
2. Synthesis and characterization of ligands and complexes 5
3. NMR spectroscopy 8
4. High-resolution mass spectrometry 21
5. Synthesis of metallopeptides Ir-BN and Ru-BN 24
6. Characterization of metallopeptides Ir-BN and Ru-BN 25
7. X-ray diffraction 32
8. Photostability 34
9. Determination of pK_a 35
10. Singlet oxygen generation 36
11. Photocatalytic oxidation of NADH 36
12. Bombesin receptor blocking experiments 37
13. Intracellular fluorescence of the compounds 38
14. Lipophilicity and self-aggregation studies 38
15. References 40

1. General information and methods

Starting materials. The synthesis of the Ir(III) dimeric precursor, $[Ir(ppy)_2(\mu\text{-}Cl)]_2$, the Ru(II) precursor, $[Ru(bpy)_2Cl_2]$, and ligands are well described in the Supporting Information. $IrCl_3{\cdot}xH_2O$ and $RuCl_3{\cdot}xH_2O$ were purchased from Johnson Matthey and used as received. The reagents tryptamine, 2,2′-bipyridyl, 2-phenylpyridine, activated MnO_2, and 5-bromo-2-pyridinecarboxaldehyde were purchased from Sigma-Aldrich; sodium hydride, methyl iodide, methyl acrylate, palladium acetate, 1,4-Diazabicyclo[2.2.2]octane (DABCO), were purchased from TCI Chemicals. All of them were used without further purification. Deuterated solvents (DMSO-d_6 and D_2O) were obtained from Eurisotop. Conventional solvents such as N,N-dimethylformamide (Scharlau), diethyl ether (Fisher Scientific), 2-ethoxyethanol (Across Organics), ethanol (Scharlau), methanol (Scharlau), dichloromethane (Scharlau), anisole (Across Organics), were degassed and in most of the cases distilled prior to use.

X-ray crystallography. A summary of crystal data collection and refinement parameters for **Ir-Me** are given in Table S3. Single crystals of **Ir-Me** were coated in high-vacuum grease and mounted on a glass fiber on a Bruker APEX-II CCD diffractometer equipped with a graphite monochromated Cu-Kα radiation source (λ = 1.54178 Å). The crystal was kept at 230.0 K during data collection. The highly redundant datasets were integrated using SAINT[1] and corrected for Lorentz and polarization effects. The absorption correction was based on the function fitting to the empirical transmission surface as sampled by multiple equivalent measurements with the program SADABS.[2] Using Olex2,[3] the structure was solved with the SHELXT[4] structure solution program using Intrinsic Phasing and refined with the SHELXL[5] refinement package using Least Squares minimization. All non-hydrogen atoms were refined with anisotropic displacement coefficients. Hydrogen atoms were placed using a "riding model" and included in the refinement at calculated positions. A solvent mask was calculated, and 18 electrons were found in a volume of 644 (A^3) in 1 void per unit cell. This is consistent with the presence of 0.5[H_2O] per Asymmetric Unit which account for 20 electrons per unit cell. CCDC reference number for **Ir-Me** is 2361042.

Measurements of UV-Vis absorption and photoluminescence spectra. UV-Vis absorption spectra were recorded on a Jasco V-750 UV-Visible spectrophotometer while emission spectra were recorded on a FLS980 spectrofluorometer (from Edinburgh Instruments) equipped with triple grating turret monochromators and a Red PMT Sphere detector. The F980 spectrometer operating software was used to collect and process fluorescence data. For the fluorescence measurements, samples of 10^{-5} M solutions in H_2O:DMSO (99:1) were prepared and deoxygenated in a Schlenk using Freeze-Pump-Thaw (FPT) technique and transferred under inert atmosphere into 10 mm quartz cells equipped with Teflon septum. The luminescence emission spectra of Ir(III) and Ru(II) complexes were recorded by exciting at 405 and 450 nm respectively, with a Xenon Arc lamp. The photoluminescence quantum yields (PLQY) were calculated by using

an integrating sphere. For the determination of the luminescence lifetime, the fluorescence decay was measured on a FLS980 spectrofluorometer equipped with a TSCPC laser (Ir(III) complexes: λ_{ex} = 405 nm; Ru(II) complexes: λ_{ex} = 450 nm) and a REDPMT detector. The F980 spectrometer operating software was used to collect and process luminescence lifetime data.

Photostability of Ir(III) and Ru(II) complexes. The photostability of Ir(III) or Ru(II) complexes was monitored by ^1H RMN. The 1.5 × 10^{-2} M solutions of the complexes in DMSO-d_6:D_2O (3:2) at 25 °C were irradiated with blue LED light (λ_{irr} = 460 nm, 24 W) for different intervals of time. ^1H NMR spectra were acquired at t = 0, 6 and 24 hours of irradiation with 32 scans into 32 k data points over a spectral width of 16 ppm.

Singlet oxygen generation. The capacity of **Ir-Me** and **Ru-Me** to generate singlet oxygen ($^1O_2$) was determined by an indirect method using 9,10-anthracenediyl-bis(methylene) dimalonic acid (ABDA) as a probe. The reaction between ABDA and $^1O_2$ was monitored by UV-Vis and the gradual decrease of the maximum at 379 nm was used to quantify the ability of our complexes to generate $^1O_2$. Aerated solutions of ABDA (8 × 10^{-5} M) and the corresponding PS in H_2O:DMSO (50:50) were irradiated with a blue LED strip (λ_{ir} = 460 nm; 24 W) at room temperature for intervals of 5 minutes (0 min → 100 min) and UV-Vis spectra were recorded after every irradiation interval. Concentrations of PS were tuned to achieve similar values of absorbance at 460 nm ($A_{460\,nm} \approx 0.06$). The singlet oxygen quantum yield (ϕ) was determined using Rose Bengal (RB) as standard reference (ϕ_Δ = 0.75)[6] and the following equation:[7]

$$\Phi_{PS} = \Phi_{RB} \times \frac{k_{PS}}{k_{RB}} \times \frac{A_{RB}}{A_{PS}}$$

where k_{PS} and k_{RB} represent the decomposition rate constants of the photobleaching of ABDA determined by the slope calculated from the linear fit of ln(A_0/A) vs time. A_0 corresponds to the absorbance of ABDA at 379 nm before irradiation, while A is the ABDA absorbance at 379 nm at different irradiation times. A_{RB} and A_{PS} correspond to absorbance at 460 nm of Rose Bengal and **Ir-Me/Ru-Me** respectively. ϕ_{RB} is the singlet-oxygen quantum yield of RB (ϕ_{RB} = 0.75 in H_2O:DMSO (55:45)).

Photocatalytic oxidation of NADH. The photocatalytic oxidation of an aerated solution of NADH (100 μM) in H_2O:DMSO (99:1) in the presence or absence of **Ir-Me** (5 μM) was evaluated by UV-Vis. The corresponding absorption spectra were recorded at different times (0 min, 5 min, 10 min and 15 min) under blue light irradiation with blue LED light (λ_{irr} = 460 nm; 24 W) or dark conditions. A decrease in the absorption band with maxima at 338 nm was considered to confirm the photocatalytic oxidation of NADH into NAD^+.

Determination of Lipophilicity. Log $P_{oct/PBS}$ was determined by using a method based on literature.[8] In summary, n-octanol and PBS were saturated with each other by continuous stirring at room temperature for 24 hours. Both phases were then separated. Next, approximately 0.1 mg of the corresponding photosensitizer was dissolved in 1 mL of PBS-saturated n-octanol. Subsequently, 1 mL of octanol-saturated PBS was added, and the mixture was stirred vigorously at room temperature for 24 h. The two layers were separated carefully, and the absorbance of each layer was determined at the respective local maximum located around 425 nm. The log $P_{oct/PBS}$ values were determined as follows:

$$Log\ P = Log\ (\frac{Abs_{425}(Octanol)}{Abs_{425}(PBS)})$$

2. Synthesis and characterization of ligands and complexes

Synthesis of A. A mixture of tryptamine (0.400 g, 2.50 mmol), 5-bromo-2-pyridinecarboxaldehyde (0.465 g, 2.50 mmol) and 80 mL of dry anisole was stirred at 160 °C for 3 h. After 3 h, the mixture was allowed to reach room temperature and activated MnO_2 (25 equiv.) was added. The mixture was stirred at 160 °C for 21 h. Thereafter, the mixture was filtered over Celite while hot and solvent was removed under vacuum. The resulting solid was washed with hexane (2 x 10 mL) and dried under vacuum. **Yield:** grey solid (0.729 g, 2.25 mmol), 90%. **^{1}H NMR (300 MHz, DMSO-*d*$_6$, 25 °C)** δ 11.84 (s, 1H, N-H^c), 8.93 (dd, *J* = 2.4, 0.6 Hz, 1H, H^p), 8.56 (dd, *J* = 8.6, 0.6 Hz, 1H, H^s), 8.50 (d, *J* = 5.1 Hz, 1H, H^l), 8.36 – 8.14 (m, 3H, H^h, H^k, H^r), 7.85 (d, *J* = 8.1 Hz, 1H, H^e), 7.59 (t, *J* = 7.7 Hz, 1H, H^f), 7.29 (t, *J* = 7.5 Hz, 1H, H^g) ppm. **^{13}C NMR (75 MHz, DMSO-d$_6$, 25 °C)** δ 155.8 (Cq), 149.2 (CH^p), 141.1 (Cq), 139.9 (CH^r), 137.9 (CH^l), 137.1 (Cq), 133.4 (Cq), 130.1 (Cq), 128.5 (CH^f), 122.7 (CH^s), 121.7 (CH^h), 120.3 (Cq), 119.7 (CH^g), 119.7 (Cq), 116.1 (CH^k), 112.9 (CH^e) ppm. **Analysis calculated for $C_{16}H_{10}BrN_3\cdot(H_2O)_{0.3}$:** C = 58.31%; H = 3.24%; N = 12.75%; Found: C = 58.32%; H = 3.19%; N = 12.28%. **HRMS-ESI(+) (m/z):** $[M+H]^+$ calcd for $[C_{16}H_{11}BrN_3]^+$ 324.0136; found 324.0147.

Synthesis of B. A suspension of **A** (1.000 g, 3.08 mmol) in anhydrous DMF (40 mL) was stirred at 0 °C for five minutes. Then, 60% NaH (185 mg, 4.63 mmol) was added. The mixture was allowed to reach room temperature and stirred for 1 h. The mixture was cooled to -10 °C and MeI (250 µL, 4.0 mmol) was added dropwise. Thereafter, the mixture was allowed to reach room temperature and stirred overnight. Then, the solution was cooled to 0 °C and H_2O (30 mL) was added dropwise to neutralize the excess of NaH. The resulting precipitate was filtered and washed with hexane (2 x 10 mL) and dried under vacuum. **Yield:** grey solid (1.051 g, 2.95 mmol), 95.9%. **^{1}H NMR (300 MHz, DMSO-*d*$_6$, 25 °C)** δ 8.88 (d, *J* = 2.4 Hz, 1H, H^p), 8.47 (d, *J* = 5.1 Hz, 1H, H^l), 8.34 (d, *J* = 7.8 Hz, 1H, H^h), 8.33 – 8.20 (m, 2H, H^k, H^s), 7.97 (d, *J* = 8.4 Hz, 1H, H^r), 7.76 – 7.59 (m, 2H, H^e, H^f), 7.33 (t, *J* = 8.0 Hz, 1H, H^g), 3.57 (s, 3H, $CH_3^{N\text{-}Me}$). **^{13}C NMR (300 MHz, DMSO-*d*$_6$, 25 °C)** δ 156.5 (Cq), 148.8 (CH^p), 142.5 (Cq), 141.2 (Cq), 139.6 (CH^s), 137.9 (CH^l), 134.7 (Cq), 130.1 (Cq), 128.8 (CH^f), 126.7 (CH^r), 121.7 (CH^h), 120.3 (Cq), 119.9 (CH^f and Cq), 115.0 (CH^k), 110.5 (CH^e), 33.6 ($CH_3^{N\text{-}Me}$) ppm. **Analysis calculated for $C_{17}H_{12}BrN_3$:** C = 60.37%; H = 3.58%; N = 12.42%; Found: C = 60.17%; H = 3.64%; N = 12.20%. **HRMS-ESI(+) (m/z):** $[M+H]^+$ calcd for $[C_{17}H_{13}BrN_3]^+$ 338.0293; found 338.0292.

Synthesis of L1. B (0.676 g, 2.00 mmol), $Pd(OAc)_2$ (2 mol %), DABCO (4 mol %) and K_2CO_3 (0.276 g, 2.00 mmol) were suspended in dry DMF (50 mL). The mixture was deoxygenated bubbling N_2 for 15 minutes and methyl acrylate (0.272 mL, 3.00 mmol) was added under N_2. Subsequently, the flask was sealed and heated at 125 °C for 24 hours. Then, the mixture was filtered through Celite. Solvent was evaporated and the remaining solid was washed with hexane (2 x 20 mL), Et_2O(2 x 20 mL), methyl tert-butyl ether (MTBE) (2 x 10 mL) and MeOH (2 x 10 mL). **Yield:** yellow solid (0.320 g, 0.932 mmol), 46.6%. **^{1}H NMR (300 MHz, DMSO-*d*$_6$, 25 °C)** δ 9.06 (d, *J* = 2.1 Hz,

1H, H^p), 8.49 (d, *J* = 5.0 Hz, 1H, H^l), 8.42 (dd, *J* = 8.2, 2.3 Hz, 1H, H^r), 8.35 (d, *J* = 7.8 Hz, 1H, H^h), 8.28 (d, *J* = 5.0 Hz, 1H, H^k), 8.06 (d, *J* = 8.2 Hz, 1H, H^s), 7.84 (d, *J* = 16.2 Hz, 1H, H^t), 7.75 – 7.59 (m, 2H, H^e, H^f), 7.33 (t, *J* = 7.1 Hz, 1H, H^g), 6.92 (d, *J* = 16.2 Hz, 1H, H^u), 3.78 (s, 3H, CH_3^w), 3.59 (s, 3H, -CH_3) ppm. **^{13}C NMR (300 MHz, DMSO-*d*$_6$, 25 °C)** δ 166.4 (Cq), 158.9 (Cq), 148.5 (CH^p), 142.5 (Cq), 141.7 (Cq), 141.0 (CH^t), 137.86 (CH^l), 135.3 (CH^r), 134.8 (Cq), 130.1 (Cq), 129.1 (Cq), 128.8 ($CH^{e\ or\ f}$), 124.9 (CH^s), 121.6 (CH^h), 120.27 (Cq), 120.1 (CH^g), 119.9(CH^u), 115.0 (CH^k), 110.5 ($CH^{e\ or\ f}$), 51.7 (CH_3^w), 33.6 ($CH_3^{N\text{-}Me}$) ppm. **Analysis calculated for $C_{21}H_{17}N_3O_2$:** C = 73.45%; H = 4.99%; N = 12.24%; Found: C = 73.43%; H = 5.05%; N = 12.38%. **HRMS-ESI(+) (m/z):** $[M+H]^+$ calcd for $[C_{21}H_{18}N_3O_2]^+$ 344.1394; found 344.1401.

Synthesis of L2. L1 (0.500 g, 1.456 mmol) was suspended in 20 mL of 6M $NaOH_{(aq)}$:EtOH (1:1). The corresponding mixture was refluxed overnight. After cooling down, the solution was acidified with 2M $HCl_{(aq)}$ until a yellow precipitate is formed. The solid was filtrated, washed with H_2O (3 x 5 mL) and dried under vacuum. **Yield:** yellow solid (0.430 g, 1.306 mmol), 89.7%. **^{1}H NMR (300 MHz, DMSO-*d*$_6$, 25 °C)** δ 12.62 (bs, 1H, H^{COOH}), 9.07 (s, 1H, H^p), 8.53 (d, *J* = 5.2 Hz, 1H, H^l), 8.45 – 8.36 (m, 3H, H^h, H^k, H^r), 8.07 (d, *J* = 8.2 Hz, 1H, H^s), 7.81 – 7.66 (m, 3H, H^e, H^f, H^t), 7.38 (t, *J* = 7.2 Hz, 1H, H^g), 6.83 (d, *J* = 16.1 Hz, 1H, H^u), 3.60 (s, 3H, -CH_3) ppm. **^{13}C NMR (300 MHz, DMSO-*d*$_6$, 25 °C)** δ 167.2 (Cq), 157.0 (Cq), 148.6 (CH^p), 143.1 (Cq), 140.5 (CH^t), 136.3 (CH^r), 135.3 (CH^l), 134.5 (Cq), 131.1 (Cq), 129.8 (Cq), 129.5 ($CH^{e\ or\ f}$), 125.4 (CH^s), 122.0 (CH^h), 122.0 (CH^u), 120.5 (Cq), 120.3 (CH^g), 120.1 (Cq), 115.4 (CH^k), 110.7 ($CH^{e\ or\ f}$), 33.6 ($CH_3^{N\text{-}Me}$) ppm. **Analysis calculated for $C_{20}H_{15}N_3O_2{\cdot}(H_2O)_{0.45}$:** C = 71.18%; H = 4.75%; N = 12.45%; Found: C = 71.18%; H = 4.92%; N = 12.33%. **HRMS-ESI(+) (m/z):** $[M+H]^+$ calcd for $[C_{20}H_{16}N_3O_2]^+$ 330.1237; found 330.1238.

Synthesis of Ir-Me. In a 100 mL Schlenk flask, previously purged with nitrogen, **L1** (45.0 mg, 0.131 mmol) and $[Ir(ppy)_2(\mu\text{-}Cl)]_2$ (67.0 mg, 0.062 mmol) were dissolved in a mixture of solvents DCM:MeOH (2:1, 25 mL). The mixture was stirred overnight at 50 °C under a nitrogen atmosphere. The solvent was removed under vacuum and the crude solid was purified by column chromatography on neutral alumina using DCM:MeOH (100:3) as mobile phase. **Yield**: orange solid (38.1 mg, 0.043 mmol), 35%. **^{1}H NMR (300 MHz, DMSO-*d*$_6$, 25 °C)** δ 8.62 (dd, *J* = 8.6, 2.1 Hz, 1H, H^r), 8.44 (d, *J* = 5.6 Hz, 1H, H^k), 8.41 (d, *J* = 8.3 Hz, 1H, H^s), 8.34 (d, *J* = 7.9 Hz, 1H, H^h), 8.29 (d, *J* = 8.4 Hz, 1H, $H^{3\ or\ 3'}$), 8.21 (d, *J* = 8.3 Hz, 1H, $H^{3\ or\ 3'}$), 8.16 (d, *J* = 5.8 Hz, 1H, $H^{6\ or\ 6'}$), 8.00 (d, *J* = 2.0 Hz, 1H, H^p), 7.99 – 7.76 (m, 7H, H^4, $H^{4'}$, $H^{6\ or\ 6'}$, H^9, $H^{9'}$, H^e, H^f), 7.75 (d, *J* = 5.5 Hz, 1H, H^l), 7.54 – 7.41 (m, 2H, H^g, H^t), 7.16 – 6.98 (m, 4H, H^5, $H^{5'}$, H^{10}, $H^{10'}$), 6.98 – 6.88 (m, 2H, H^{11}, $H^{11'}$), 6.57 (d, *J* = 16.1 Hz, 1H, H^u), 6.27 (dd, *J* = 7.6, 1.2 Hz, 1H, $H^{12\ or\ 12'}$), 6.23 (dd, *J* = 7.6, 1.3 Hz, 1H, $H^{12\ or\ 12'}$), 3.93 (s, 3H, $CH_3^{N\text{-}Me}$), 3.73 (s, 3H, CH_3^w) ppm. **^{13}C NMR (75 MHz, DMSO-*d*$_6$, 25 °C)** δ 166.7 (Cq), 166.5 (Cq), 165.6 (Cq), 156.3 (Cq), 150.0 (CH), 149.8 (CH), 149.14 (CH), 149.10 (CH), 145.9 (Cq), 143.99 (Cq), 143.97 (Cq), 139.6 (CH), 139.5 (Cq), 138.8 (CH), 138.7 (CH), 138.6 (CH), 138.4 (Cq), 136.4 (CH), 134.0 (Cq), 132.4(Cq), 131.4 (CH), 131.1 (2CH), 130.3 (CH), 130.2 (CH), 128.5 (CH), 125.0 (CH), 124.9 (CH), 123.8 (2CH), 122.5 (CH), 122.3 (CH), 122.3 (Cq), 122.4 (Cq), 122.2 (CH), 122.00 (CH), 120.3 (Cq), 120.1 (CH), 119.7 (CH), 119.1 (CH), 112.0 (CH), 51.9 (CH_3), 36.6 (CH_3) ppm. **Analysis calculated for $C_{43}H_{33}ClIrN_5O_2{\cdot}(H_2O)_{0.2}$:** C = 58.49%; H = 3.81%; N = 7.93%; Found: C = 58,44%; H = 3.78%; N = 7.68%. **HRMS-ESI(+) (m/z):** $[M]^+$ calcd for $[C_{43}H_{33}IrN_5O_2]^+$ 844.2264; found 844.2273.

Synthesis of Ir-H. In a 100 mL Schlenk flask, previously purged with nitrogen, **L2** (62.0 mg, 0.187 mmol) was added to a solution of $[Ir(ppy)_2(\mu\text{-}Cl)]_2$ (100 mg, 0.093 mmol) in a mixture of solvents DCM:MeOH (2:1, 25 mL).The mixture was stirred overnight at 50 °C under a nitrogen atmosphere. Then, solvent was removed and the corresponding solid was redissolved in MeOH (2 mL) and precipitated with Et_2O (15 mL). Then, the orange solid was washed with Et_2O (3 x 15 mL) and dried under vacuum for 5 hours. **Yield**: orange solid (93.8 mg, 0.108 mmol), 58.3%. **^{1}H NMR (300 MHz, DMSO-*d*$_6$, 25 °C)** δ 12.93 (bs, 1H, H^{COOH}), 8.58 (dd, *J* = 8.6, 2.1 Hz, 1H, H^r), 8.43 (d, *J* = 5.6 Hz, 1H, H^k), 8.39 (d, *J* = 8.7 Hz, 1H, H^s), 8.33 (d, *J* = 7.9 Hz, 1H, H^h), 8.28 (d, *J* = 8.4 Hz, 1H, $H^{3 \text{ or } 3'}$), 8.20 (d, *J* = 8.4 Hz, 1H, $H^{3 \text{ or } 3'}$), 8.15 (d, *J* = 5.7 Hz, 1H, $H^{6 \text{ or } 6'}$), 8.01 – 7.80 (m, 7H, H^9, $H^{9'}$, H^4, $H^{4'}$, H^e, H^f, H^p), 7.78 (d, *J* = 5.7 Hz, 1H, $H^{6 \text{ or } 6'}$), 7.74 (d, *J* = 5.5 Hz, 1H, H^l), 7.47 (t, *J* = 7.4 Hz, 1H, H^g), 7.37 (d, *J* = 16.0 Hz, 1H, H^t), 7.16 – 6.97 (m, 4H, H^{10}, $H^{10'}$, H^5, $H^{5'}$), 7.01 – 6.87 (m, 2H, H^{11}, $H^{11'}$), 6.44 (d, *J* = 16.1 Hz, 1H, H^u), 6.26 (dd, *J* = 7.6, 1.2 Hz, 1H, $H^{12'}$), 6.23 (dd, *J* = 7.5, 1.3 Hz, 1H, H^{12}), 3.92 (s, 3H, $CH_3^{N\text{-}Me}$) ppm. **^{13}C NMR (75 MHz, DMSO-*d*$_6$, 25 °C)** δ 166.7 (Cq), 166.5 (Cq), 156.1 (Cq), 150.0 (Cq), 149.8 (Cq), 149.7 (CH), 149.1 (CH), 148.9 (CH), 145.9 (Cq), 144.00 (Cq), 143.97 (Cq), 139.6 (Cq), 139.6 (CH), 138.8 (CH), 138.6 (CH), 138.4 (CH), 138.0 (CH), 137.9 (Cq), 136.4 (CH), 134.0 (Cq), 132.6 (Cq), 131.4 (CH), 131.2 (CH), 131.1 (CH), 130.2 (CH), 130.1 (CH),128.5 (CH), 125.0 (CH), 124.9 (CH), 123.8 (CH), 123.8 (CH), 122.5 (CH), 122.34 (CH), 122.30 (Cq), 122.24 (CH), 121.99 (CH), 120.3 (Cq), 120.1 (CH), 119.7 (CH), 119.0 (CH), 112.0 (CH), 36.6 ($CH_3^{N\text{-}Me}$). **Analysis calculated for $C_{42}H_{31}ClIrN_5O_2\cdot(H_2O)_{0.2}$:** C = 58.05%; H = 3.64%; N = 8.06%; Found: C = 58.01%; H = 3.64%; N = 7.86%. **HRMS-ESI(+) (m/z):** $[M]^+$ calcd for $[C_{42}H_{31}IrN_5O_2]^+$ 830.2107; found 830.2100.

Synthesis of Ru-Me. In a 100 mL round-bottom pressure flask, **L1** (75.2 mg, 0.219 mmol) and $Ru(bpy)_2Cl_2$ (100 mg, 0.206 mmol) were suspended in 35 mL of EtOH:H_2O (1:1). The mixture was deoxygenated by bubbling N_2 for 20 minutes. Then, the mixture was heated at 120 °C and stirred during 24 h. After 24 h, solvent was removed and the solid was purified on neutral alumina using DCM:MeOH (100:2 → 100:5) as mobile phase. The corresponding fractions were collected and solvent was removed under vacuum. The solid was dried under vacuum for 6 hours. **Yield**: garnet solid (80.1 mg, 0.097 mmol), 47.0%. **^{1}H NMR (300 MHz, DMSO-*d*$_6$)** δ 8.96 – 8.76 (m, 4H, 4H^{bpy}), 8.61 (dd, *J* = 8.7, 1.9 Hz, 1H, H^r), 8.43 – 8.32 (m, 2H, H^h, H^s), 8.30 (d, *J* = 5.7 Hz, 1H, H^k), 8.23 – 8.03 (m, 6H, H^p, 5H^{bpy}), 7.88 (d, *J* = 8.3 Hz, 1H, H^e), 7.84 – 7.73 (m, 3H, H^f, 2H^{bpy}), 7.70 (d, *J* = 5.2 Hz, 1H, H^{bpy}), 7.65 (d, *J* = 16.2 Hz, 1H, H^t), 7.59 – 7.42 (m, 6H, H^g, H^l, 4H^{bpy}), 6.80 (d, *J* = 16.1 Hz, 1H, H^u), 3.94 (s, 3H, $CH_3^{N\text{-}Me}$), 3.72 (s, 3H, CH_3^w). **^{13}C NMR (75 MHz, DMSO-*d*$_6$, 25 °C)** δ 165.9 (Cq), 157.4 (Cq), 156.9 (Cq), 156.7 (Cq), 156.7 (Cq), 156.4 (Cq), 152.1 (CH), 151.8 (CH), 151.5 (2CH), 151.0 (CH), 145.5 (Cq), 140.6 (CH), 140.5 (Cq), 139.3 (CH), 138.3 (Cq), 137.9 (CH), 137.8 (CH), 137.8 (CH), 137.7 (CH), 134.0 (CH), 133.2 (Cq), 131.9 (Cq), 130.8 (CH), 127.9 (CH), 127.72 (3CH), 127.65 (CH), 124.54 (CH), 124.47 (CH), 124.39 (CH), 124.3 (CH), 122.3 (CH), 122.2 (CH), 122.1 (CH), 120.6 (Cq), 118.5 (CH), 112.1 (CH), 51.8(CH_3), 36.9 (CH_3) ppm. **Analysis calculated for $C_{41}H_{33}Cl_2N_7O_2Ru\cdot(H_2O)_{0.25}$:** C = 59.17%; H = 4.06%; N = 11.78%; Found: C = 59.20%; H = 4.32%; N = 11.25%. **HRMS-ESI(+) (m/z):** $[M]^+$ calcd for $[C_{41}H_{33}N_7O_2Ru]^{2+}$ 378.5869; found 378.5881.

Synthesis of Ru-H. In a 100 mL round-bottom high-pressure flask, **L2** (71.7 mg, 0.217 mmol) and $Ru(bpy)_2Cl_2$ (100 mg, 0.206 mmol) were suspended in 35 mL of EtOH:H_2O (1:1). The mixture was deoxygenated by bubbling N_2 for 20 minutes. Then, the mixture was heated at 120 °C and stirred during 24 h. After 24 h, the solvent was removed under vacuum and the corresponding solid

was washed with Et_2O (2 x 20 mL). The garnet solid was dried under vacuum for 6 hours. **Yield**: garnet solid (135 mg), 80.4% **^{1}H NMR (300 MHz, DMSO-*d*$_6$, 25 °C)** 12.75 (bs, 1H, H^{COOH}), 8.97 – 8.76 (m, 4H, $4H^{bpy}$), 8.56 (dd, *J* = 8.6, 1.9 Hz, 1H, H^r), 8.39 (d, *J* = 8.4 Hz, 1H, H^s), 8.35 (d, *J* = 7.7, 1H, H^h), 8.29 (d, *J* = 5.7 Hz, 1H, H^k), 8.22 – 8.03 (m, 5H, $4H^{bpy}$, H^p), 7.92 – 7.68 (m, 6H, $4H^{bpy}$, H^e, H^f), 7.61 – 7.39 (m, 7H, $4H^{bpy}$, H^g, H^l, H^t), 6.67 (d, *J* = 16.1 Hz, 1H, H^u), 3.94 (s, 3H, $CH_3^{N\text{-}Me}$) ppm. **^{13}C NMR (75 MHz, DMSO-*d*$_6$, 25 °C)** δ 166.7 (Cq), 157.1 (Cq), 156.8 (Cq), 156.74 (Cq), 156.66 (Cq), 156.4 (Cq), 151.8 (2CH), 151.5 (2CH), 151.0 (CH), 145.5 (Cq), 140.51 (Cq), 140.50 (CH), 138.5 (CH), 138.2 (Cq), 137.9 (CH), 137.79 (CH), 137.76 (CH), 137.70 (CH), 134.0 (CH), 133.2 (Cq), 132.2 (Cq), 130.8 (CH), 127.9 (CH), 127.74 (2CH), 127.65 (2CH), 124.5 (CH), 124.5 (CH), 124.4 (CH), 124.3 (CH), 123.8 (CH), 122.3 (CH), 122.0 (CH), 120.6 (Cq), 118.5 (CH), 112.1 (CH), 36.9 (CH_3) ppm. **Analysis calculated for $C_{40}H_{31}Cl_2N_7O_2Ru\cdot(H_2O)_{0.5}$:** C = 58.4%; H = 3.92%; N = 11.92%; Found: C = 58.34%; H = 4.13%; N = 11.82%. **HRMS-ESI(+) (m/z):** $[M]^+$ calcd for $[C_{40}H_{31}N_7O_2Ru]^{2+}$ 371.5791; found 371.5796.

3. NMR spectroscopy

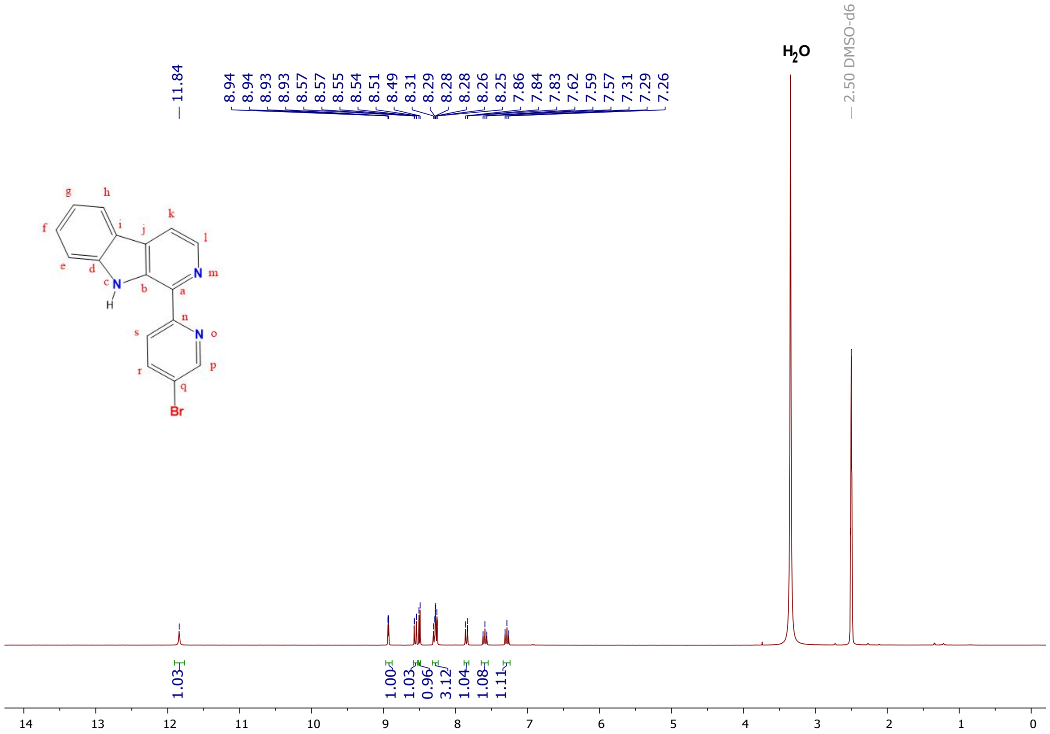

Figure S1. ^{1}H NMR (300 MHz, DMSO-d_6, 25 °C) spectrum of **A**.

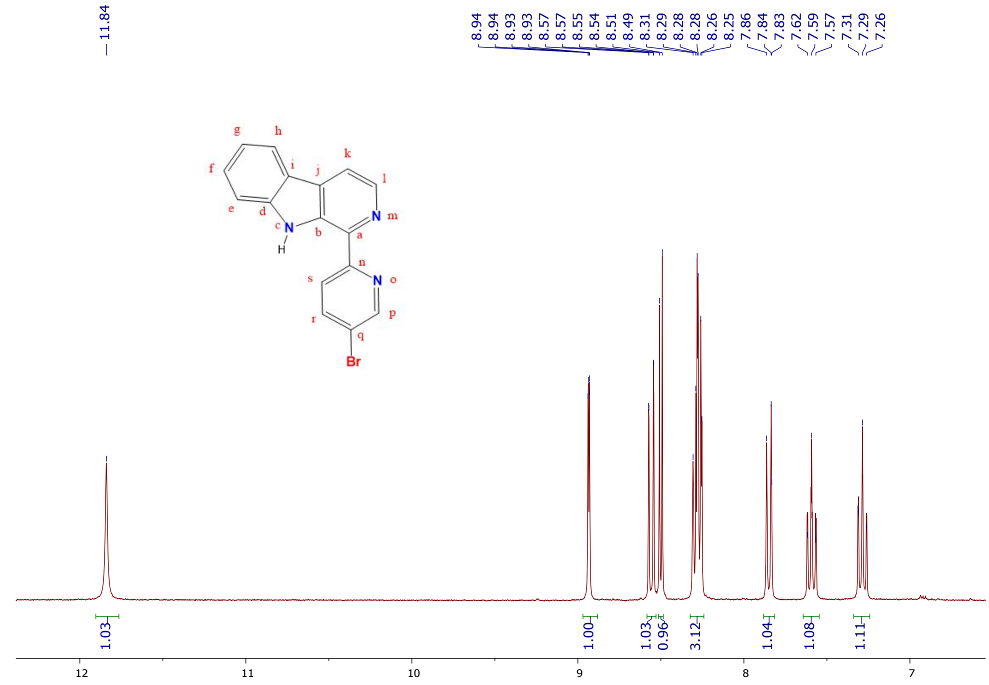

Figure S2. ^{1}H NMR (300 MHz, DMSO-d$_6$, 25 °C) spectrum of **A** in the aromatic region.

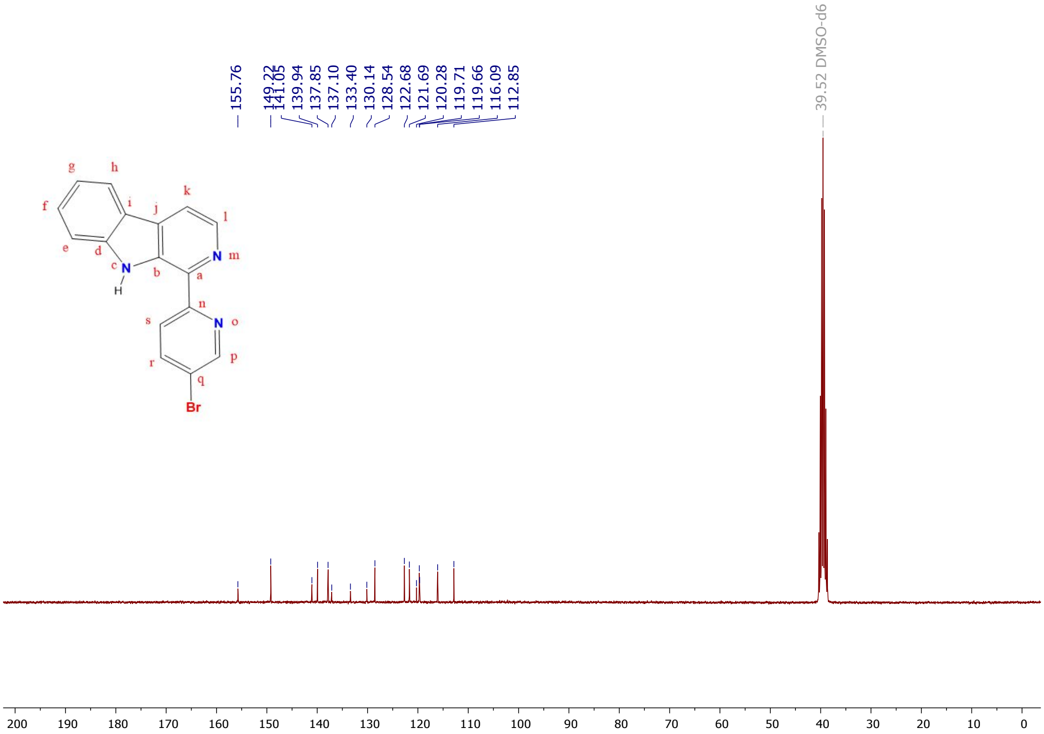

Figure S3. $^{13}C\{^{1}H\}$ NMR (75 MHz, DMSO-d$_6$, 25 °C) spectrum of **A**.

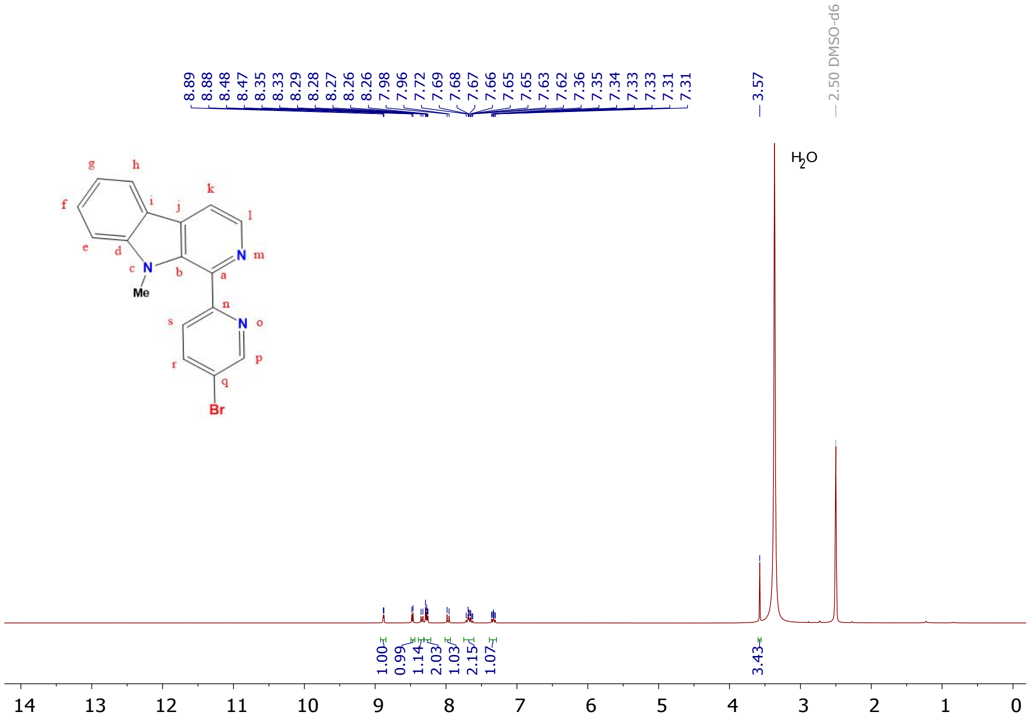

Figure S4. ^{1}H NMR (300 MHz, DMSO-d_6, 25 °C) spectrum of **B**.

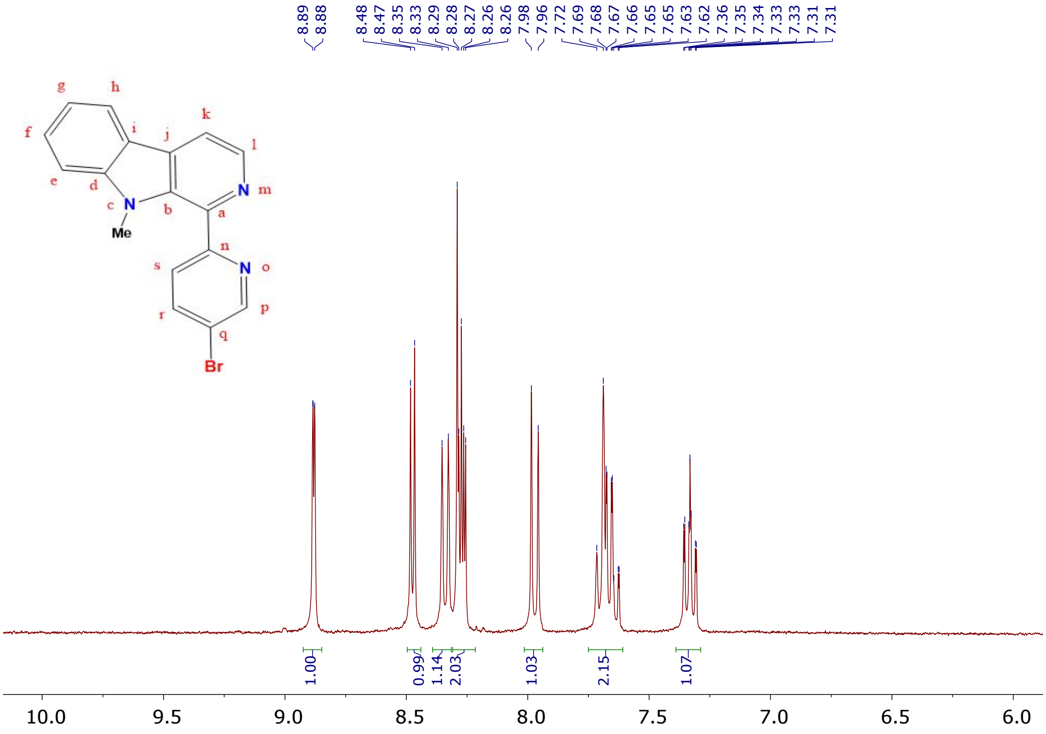

Figure S5. ^{1}H NMR (300 MHz, DMSO-d_6, 25 °C) spectrum of **B** in the aromatic region.

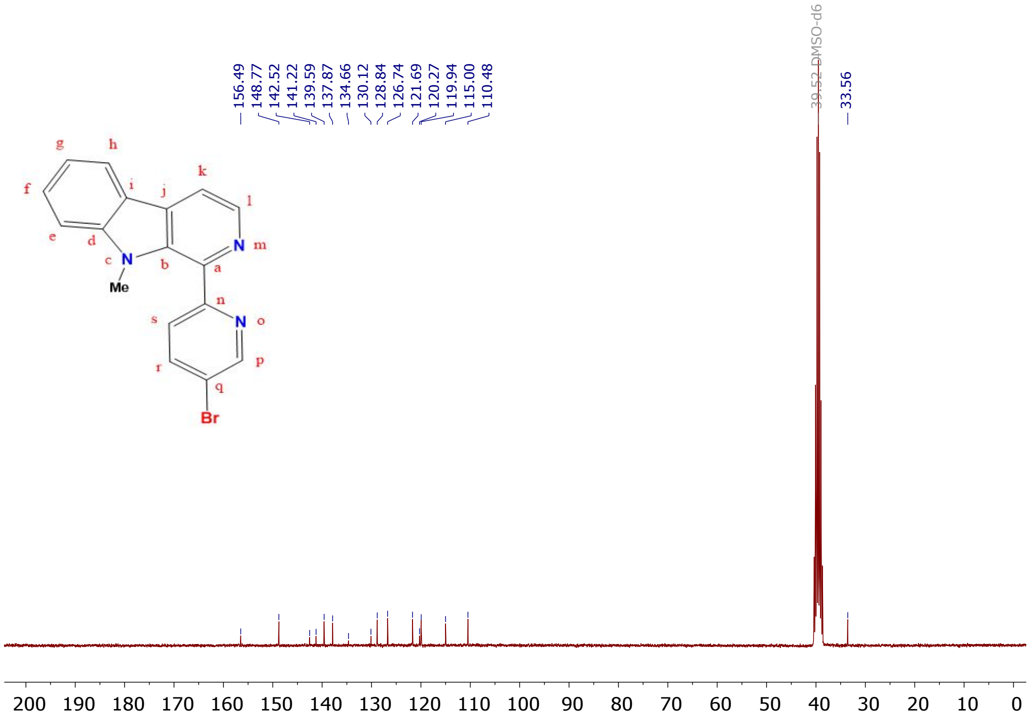

Figure S6. $^{13}C\{^1H\}$ NMR (75 MHz, DMSO-d_6, 25 °C) spectrum of **B**.

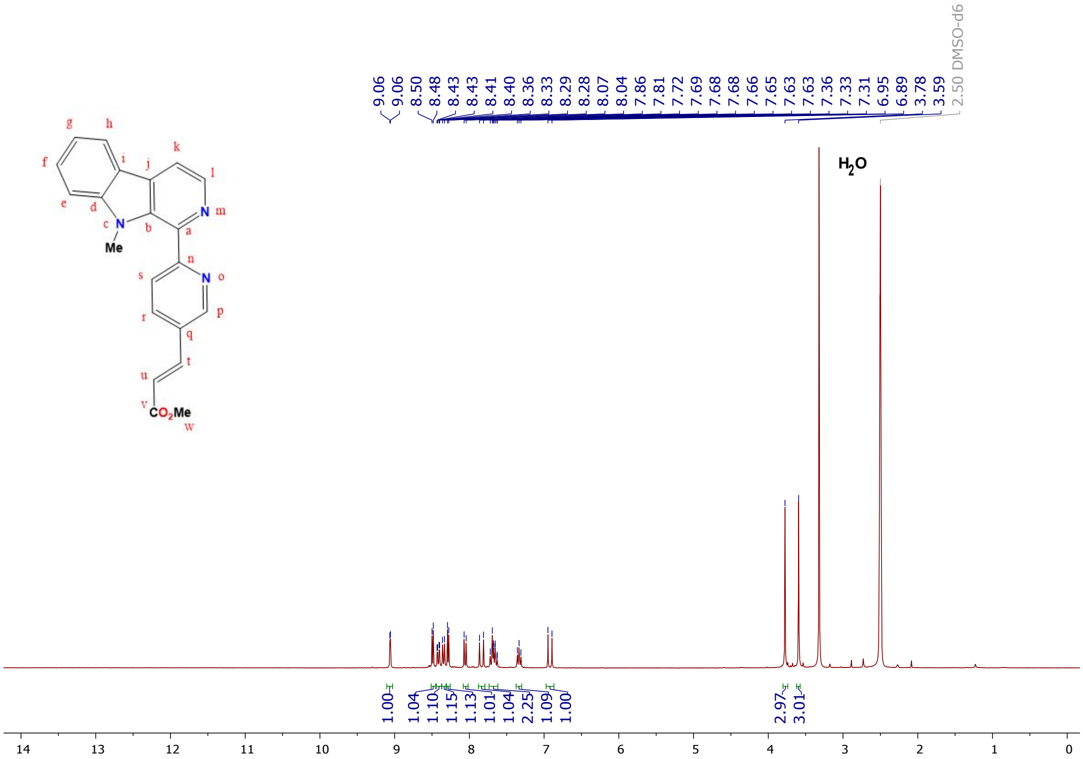

Figure S7. ^1H NMR (300 MHz, DMSO-d_6, 25 °C) spectrum of **L1**.

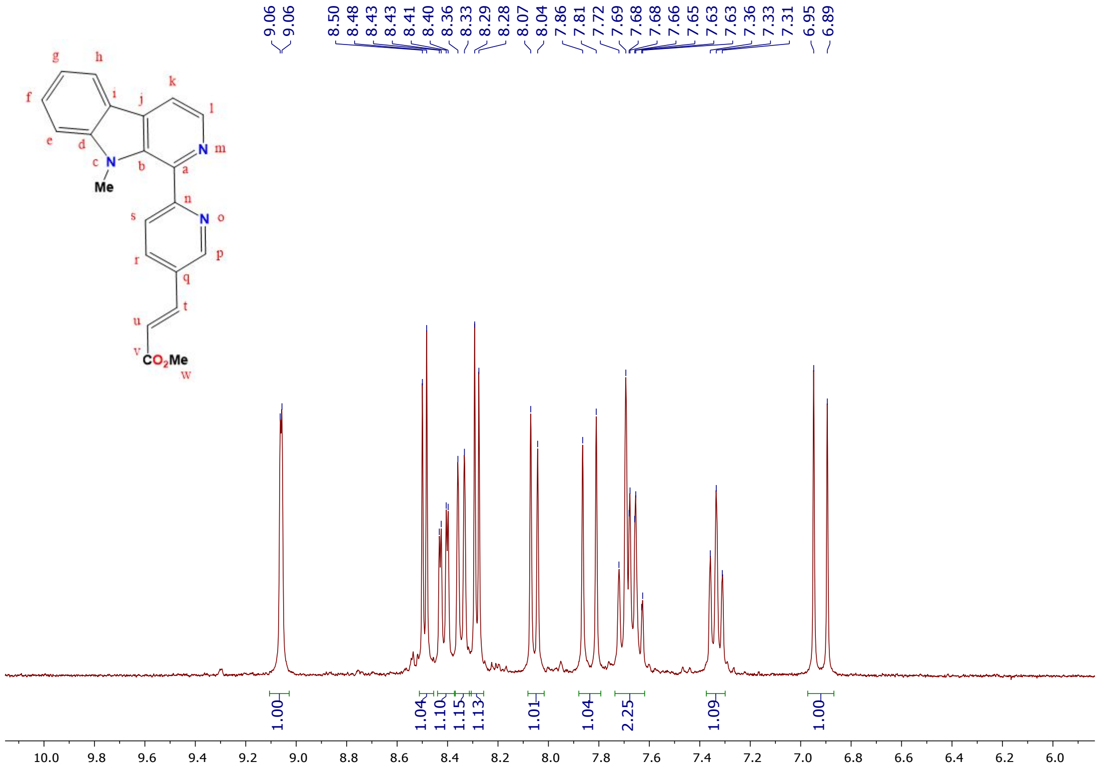

Figure S8. ^1H NMR (300 MHz, DMSO-d_6, 25 °C) spectrum of **L1 i**n the aromatic region.

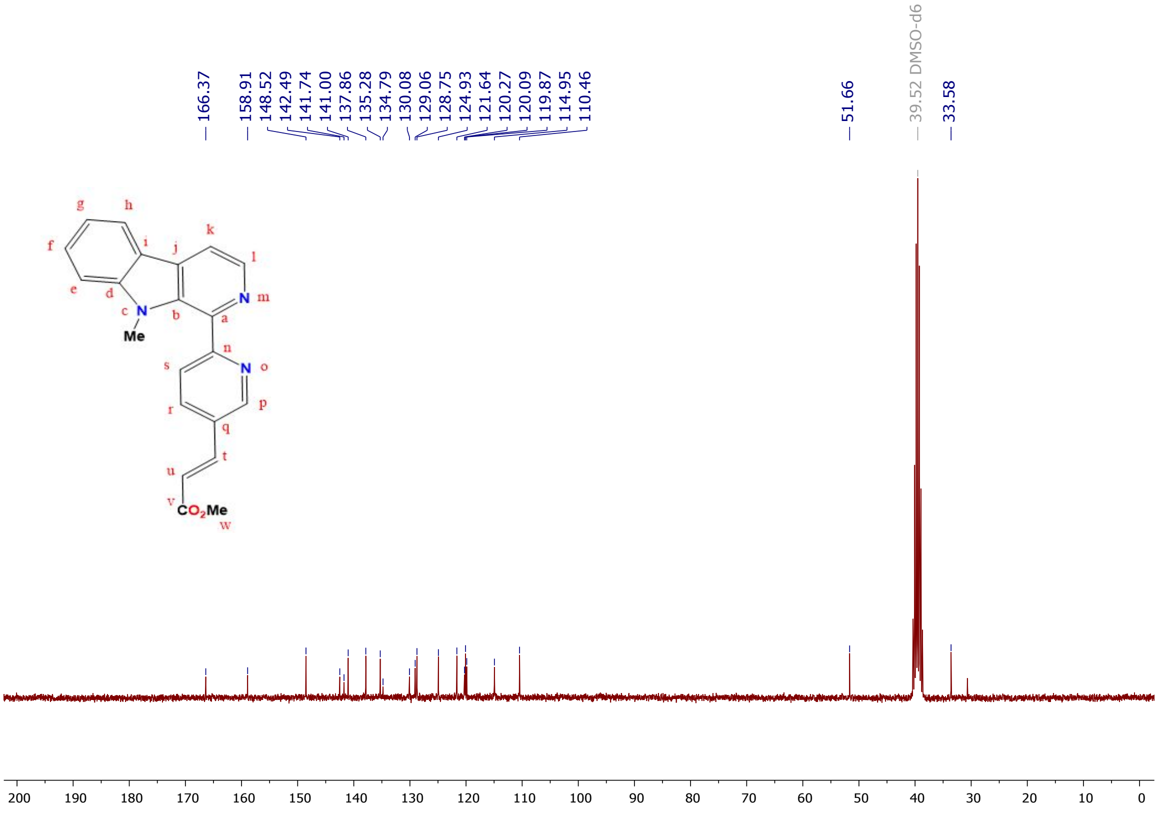

Figure S9. $^{13}C\{^1H\}$ NMR (75 MHz, DMSO-d_6, 25 °C) spectrum of **L1**.

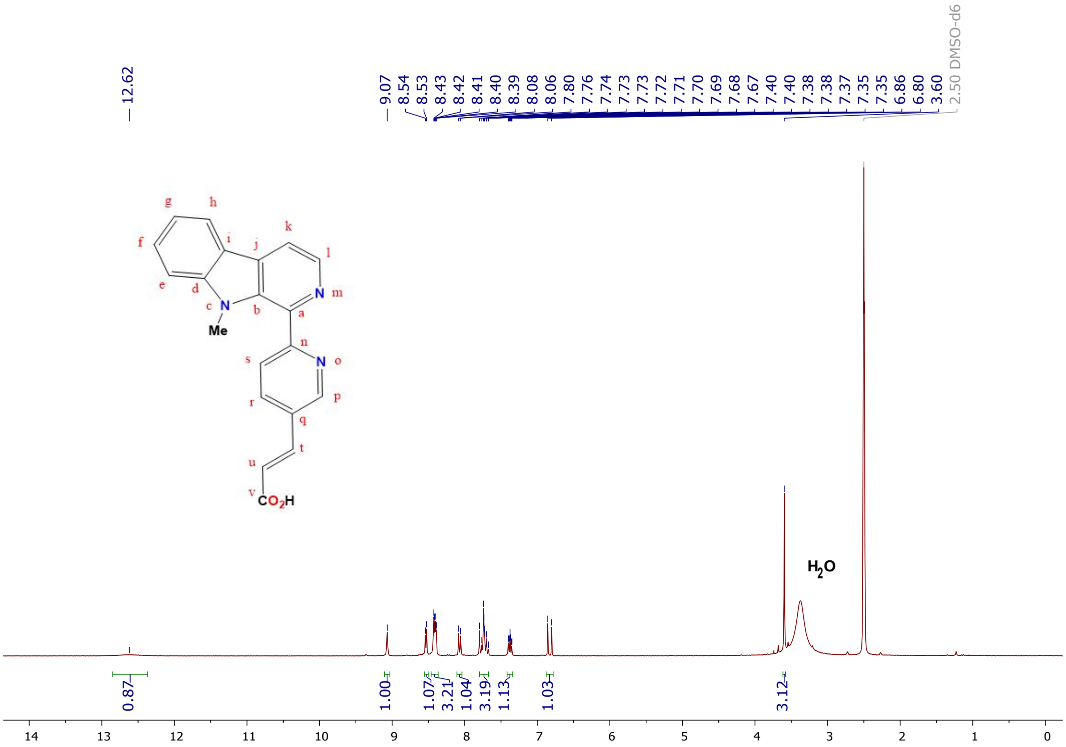

Figure S10. ^{1}H NMR (300 MHz, DMSO-d_6, 25 °C) spectrum of **L2**.

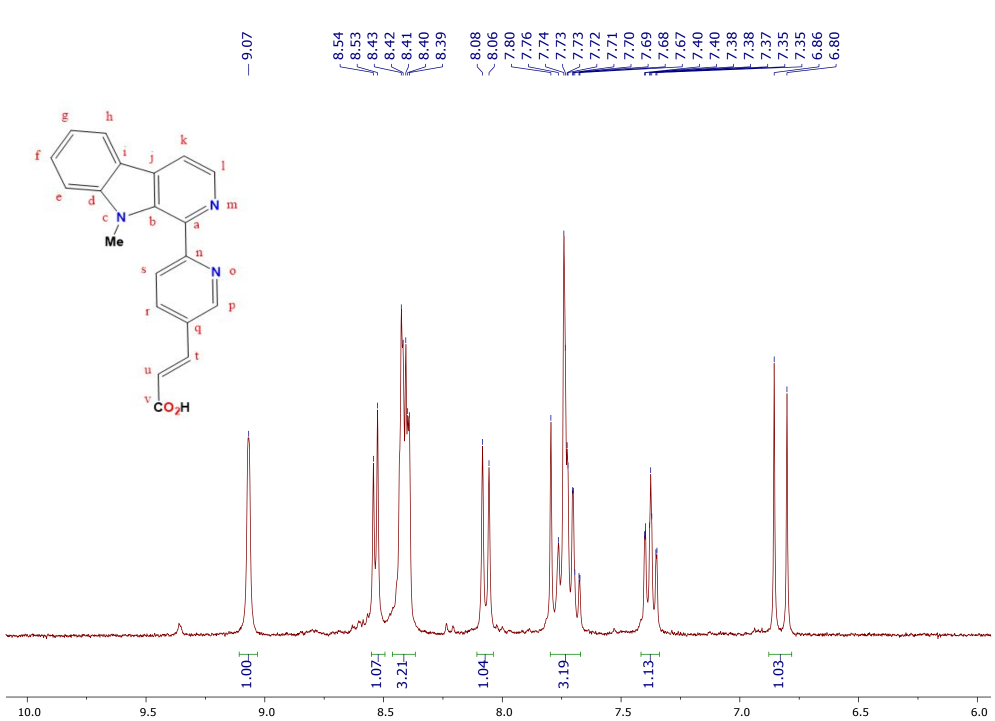

Figure S11. ^{1}H NMR (300 MHz, DMSO-d_6, 25 °C) spectrum of **L2** in the aromatic region.

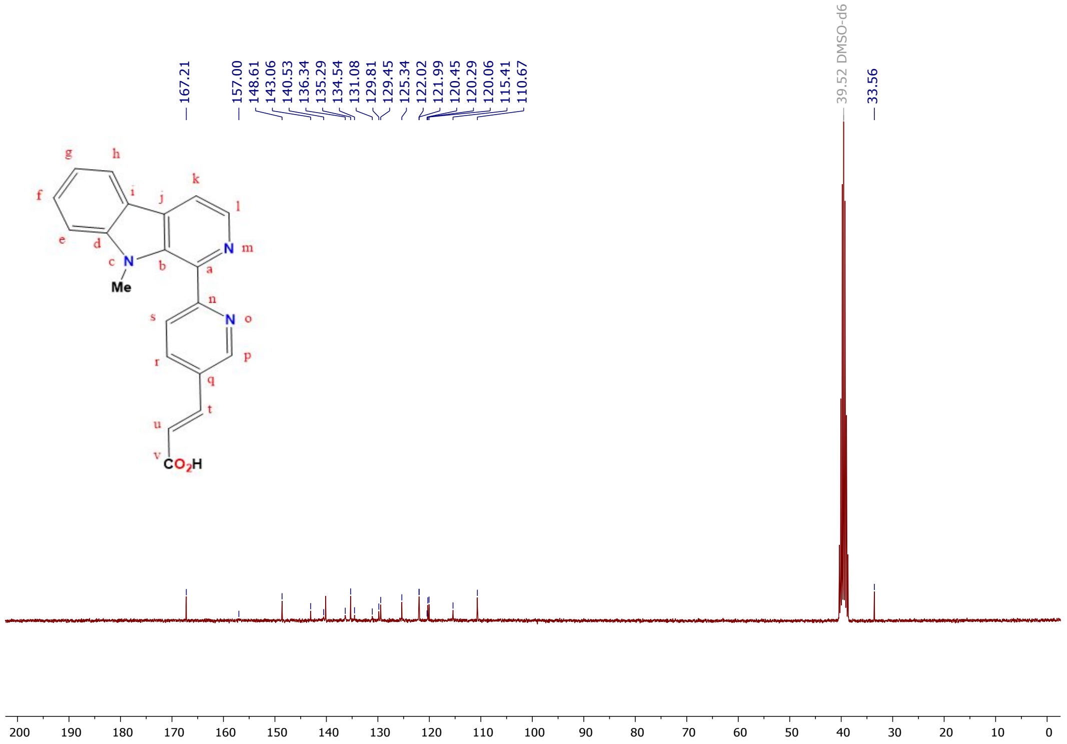

Figure S12. $^{13}C\{^{1}H\}$ NMR (75 MHz, DMSO-d_6, 25 °C) spectrum of **L2**.

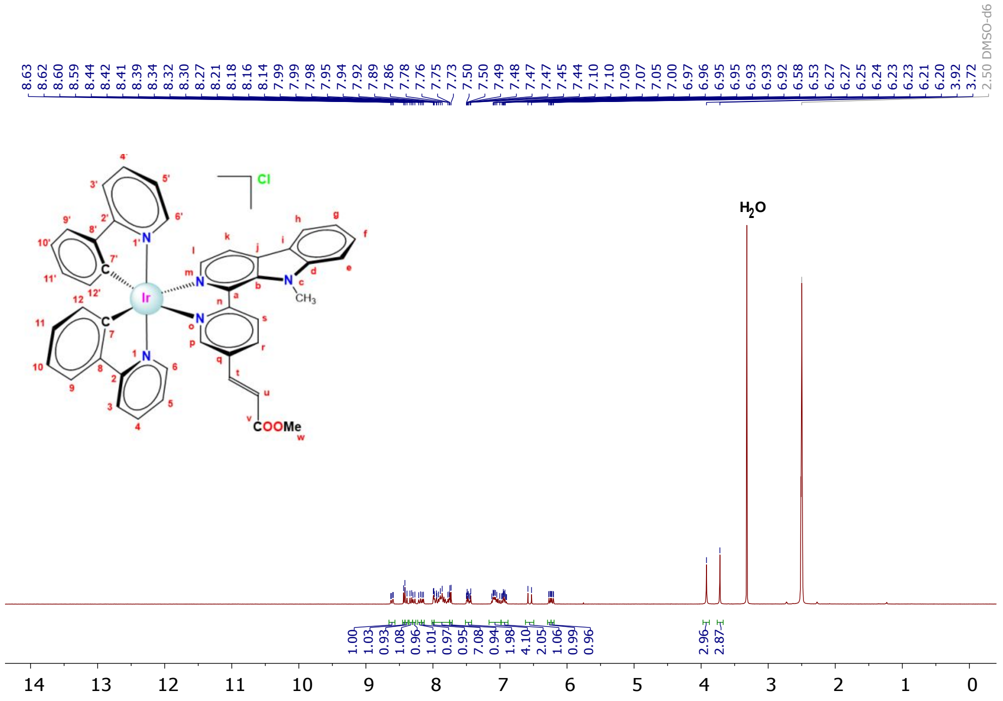

Figure S13. ^{1}H NMR (300 MHz, DMSO-d_6, 25 °C) spectrum of **Ir-Me**.

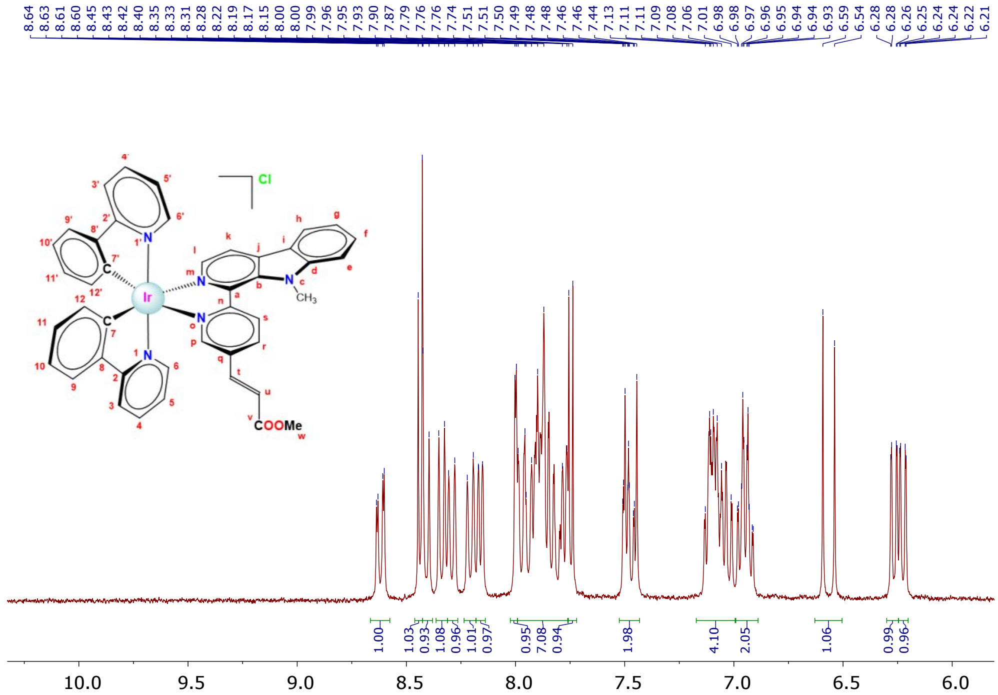

Figure S14. ^{1}H NMR (300 MHz, DMSO-d_6, 25 °C) spectrum of **Ir-Me** in the aromatic region.

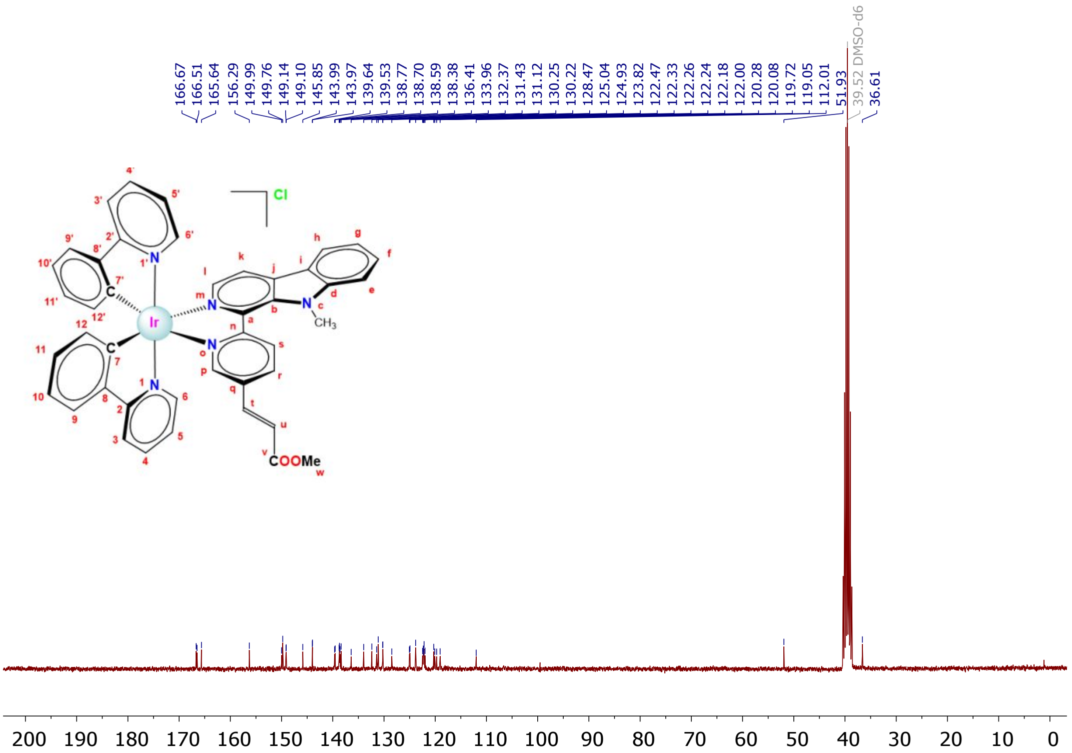

Figure S15. $^{13}C\{^1H\}$ NMR (75 MHz, DMSO-d_6, 25 °C) spectrum of **Ir-Me**.

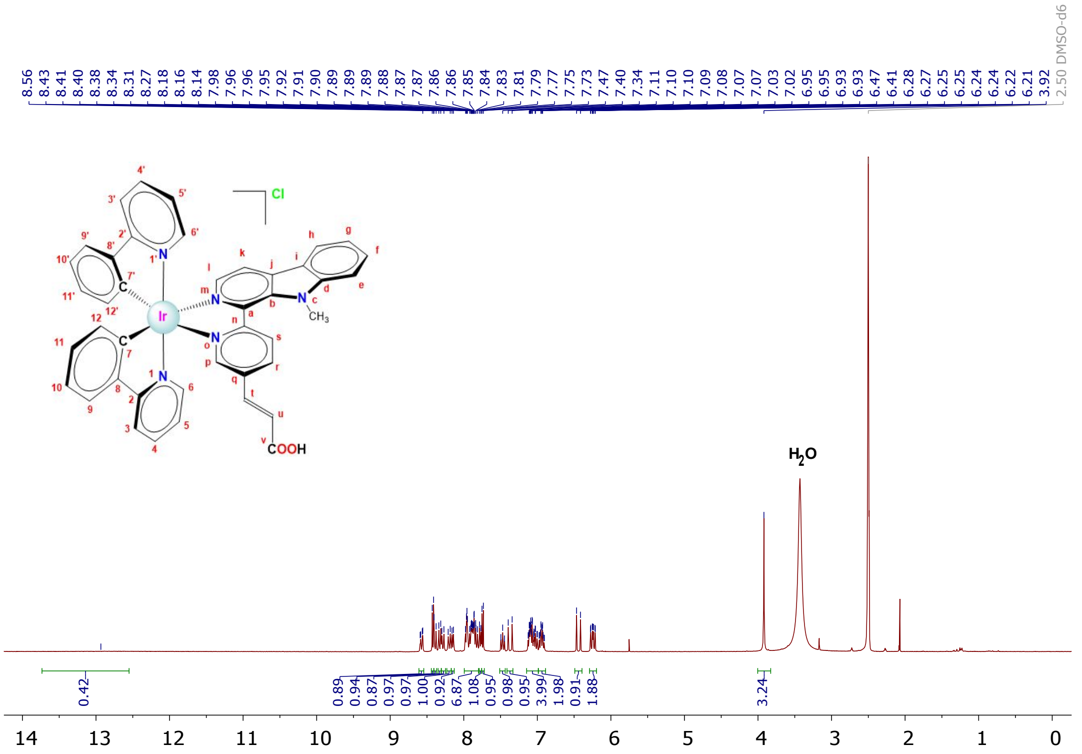

Figure S16. ^{1}H NMR (300 MHz, DMSO-d_6, 25 °C) spectrum of **Ir-H**.

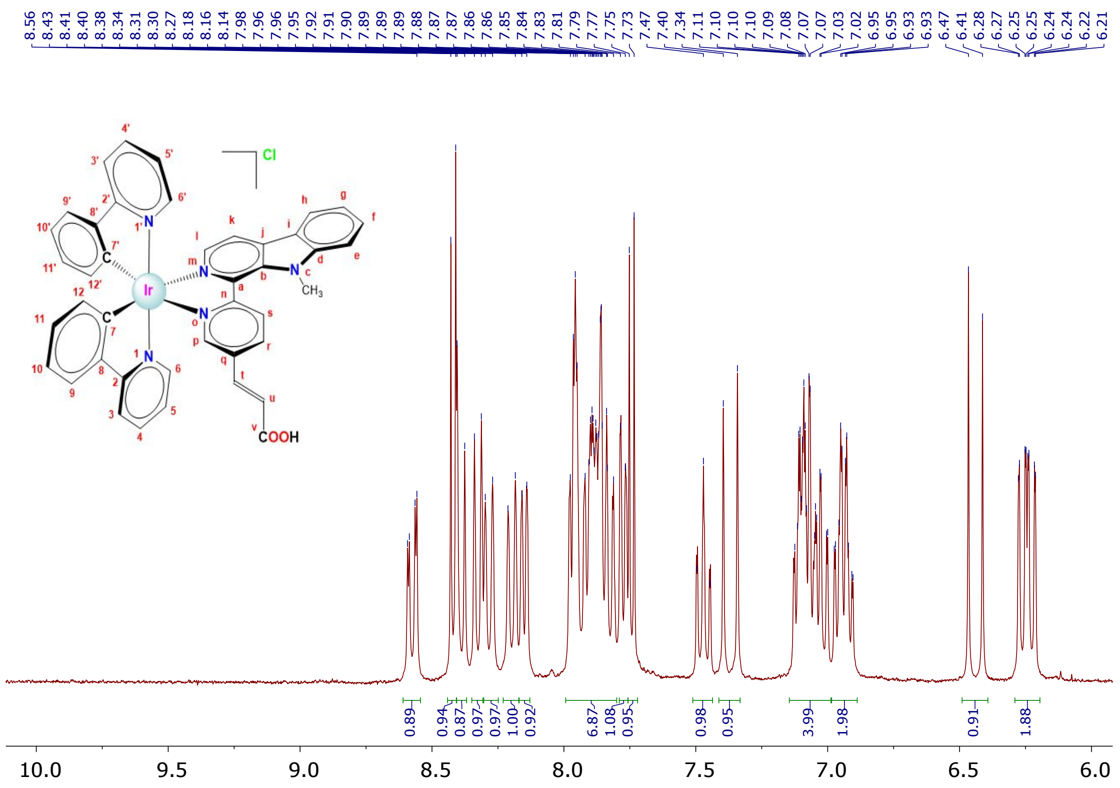

Figure S17. ^{1}H NMR (300 MHz, DMSO-d_6, 25 °C) spectrum of **Ir-H** in the aromatic region.

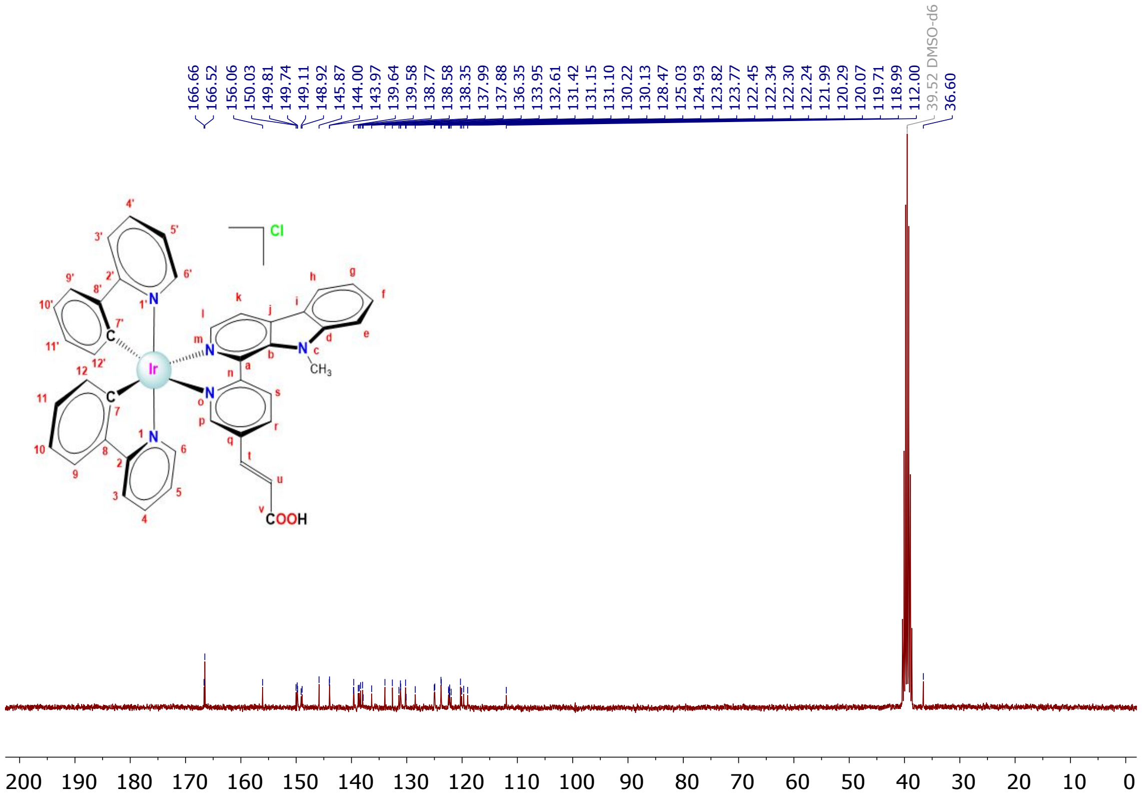

Figure S18. $^{13}C\{^{1}H\}$ NMR (75 MHz, DMSO-d_6, 25 °C) spectrum of **Ir-H**.

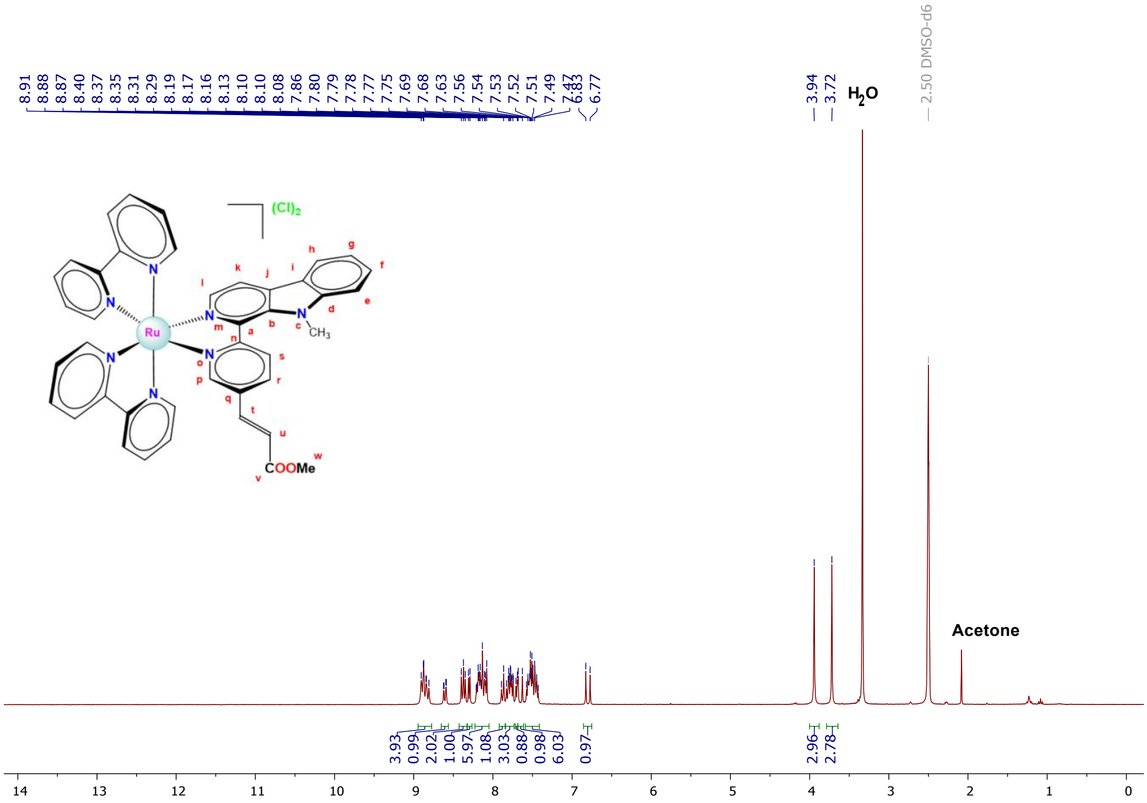

Figure S19. ^1H NMR (300 MHz, DMSO-d_6, 25 °C) spectrum of **Ru-Me**.

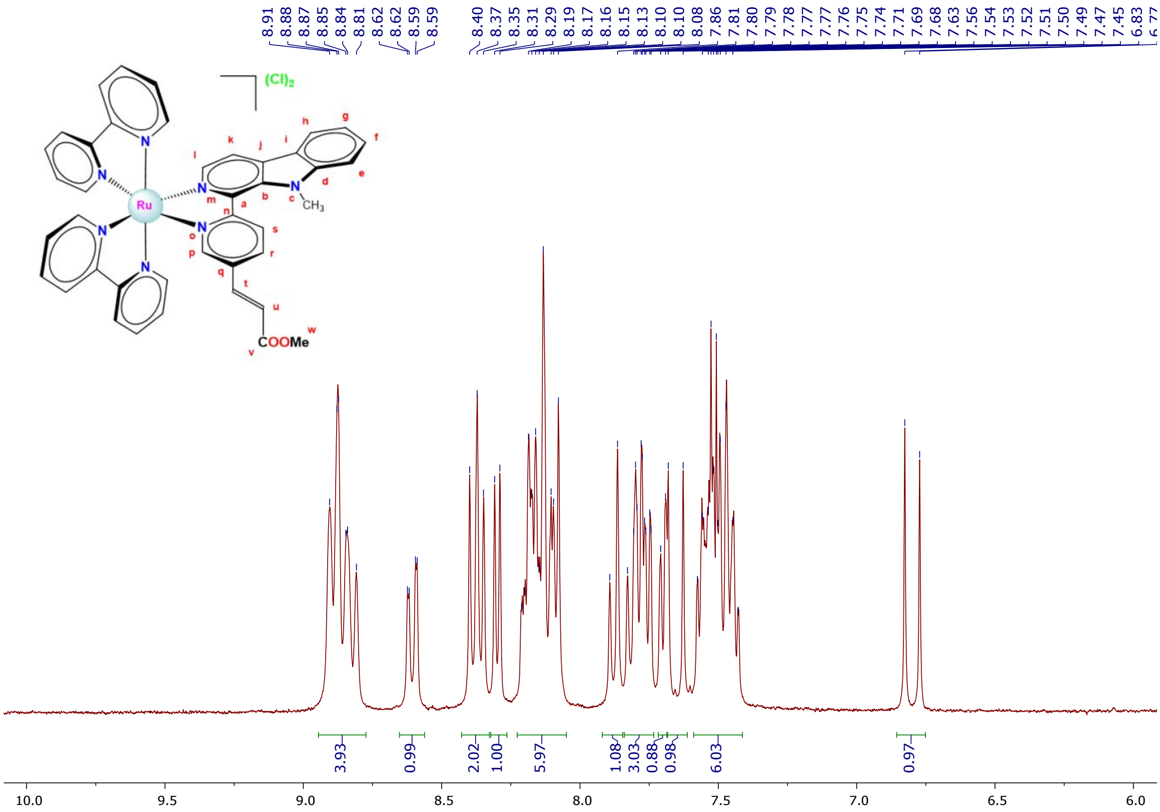

Figure S20. ^1H NMR (300 MHz, DMSO-d_6, 25 °C) spectrum of **Ru-Me** in the aromatic region.

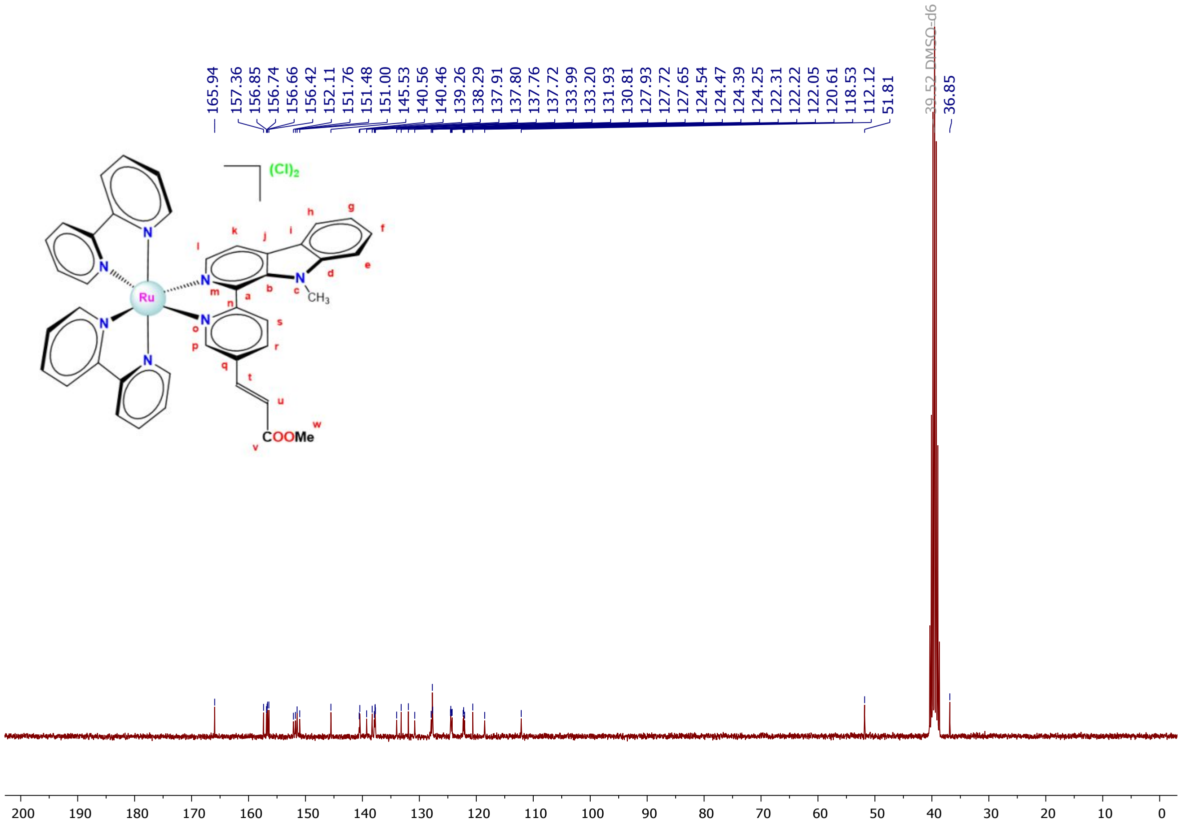

Figure S21. $^{13}C\{^{1}H\}$ NMR (75 MHz, DMSO-d_6, 25 °C) spectrum of **Ru-Me**.

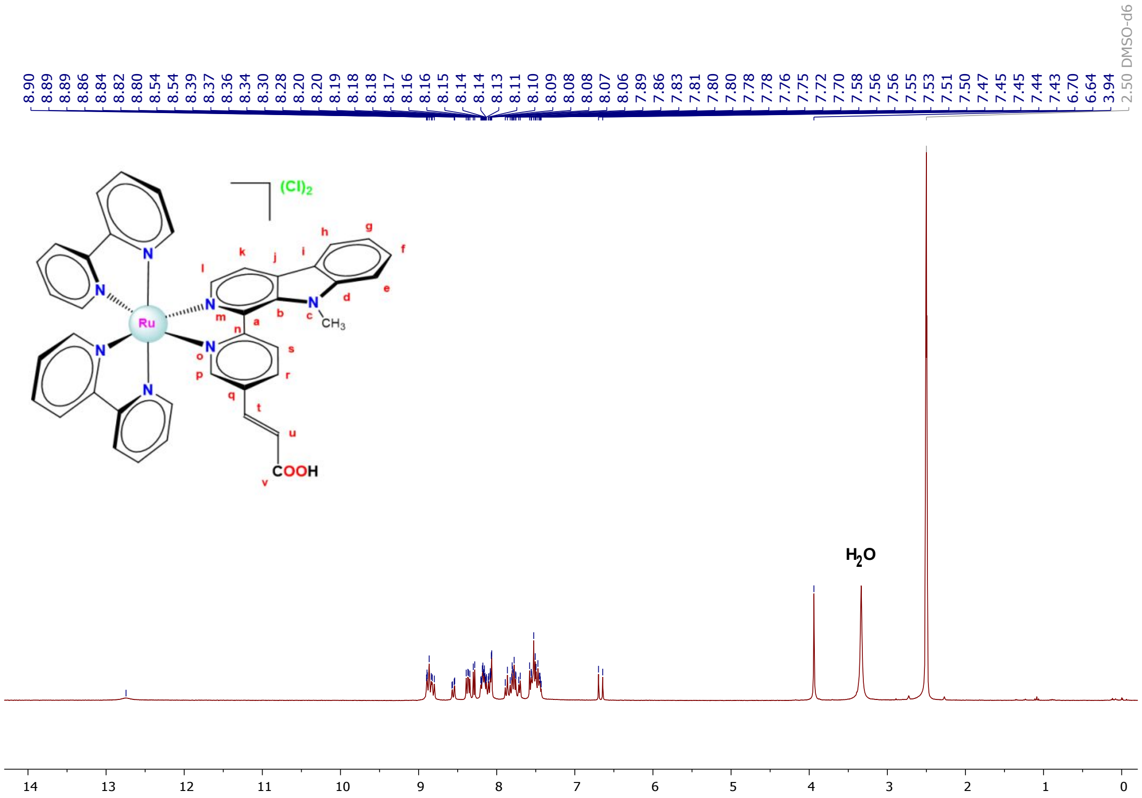

Figure S22. ^{1}H NMR (300 MHz, DMSO-d_6, 25 °C) spectrum of **Ru-H**.

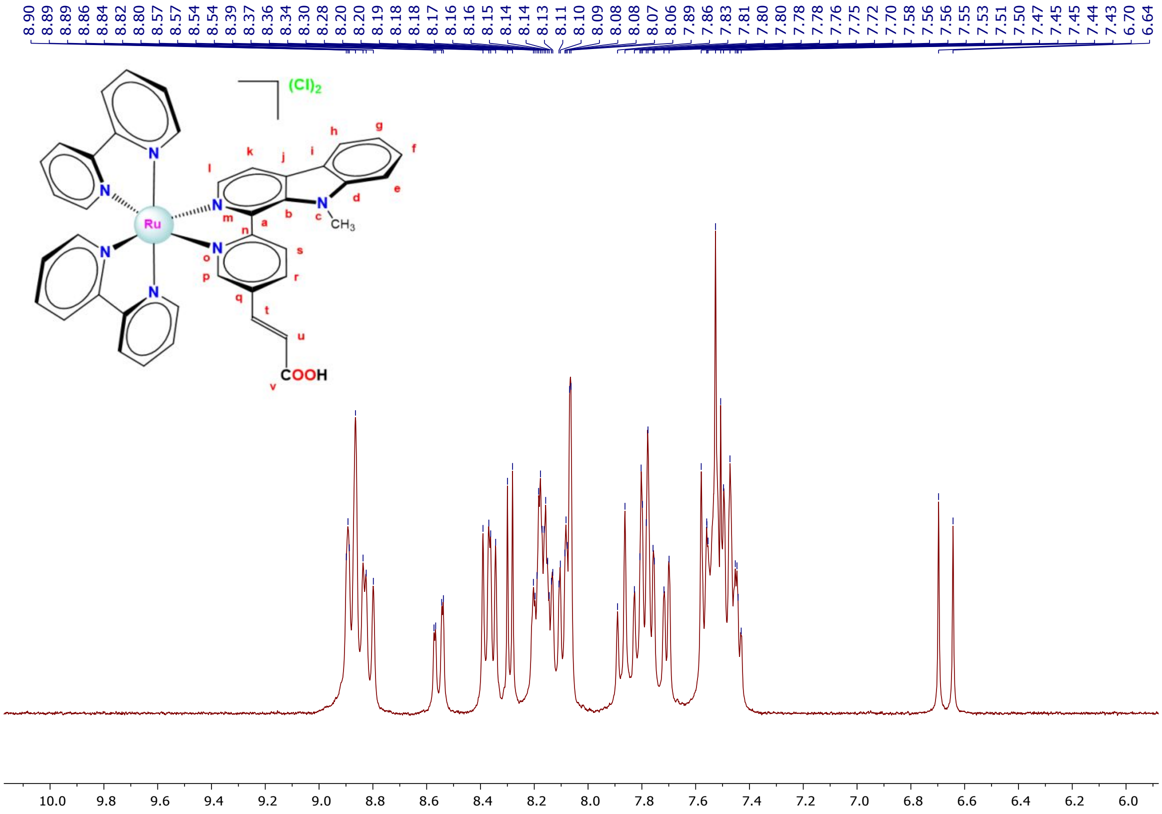

Figure S23. ^{1}H NMR (300 MHz, DMSO-d_6, 25 °C) spectrum of **Ru-H** in the aromatic region.

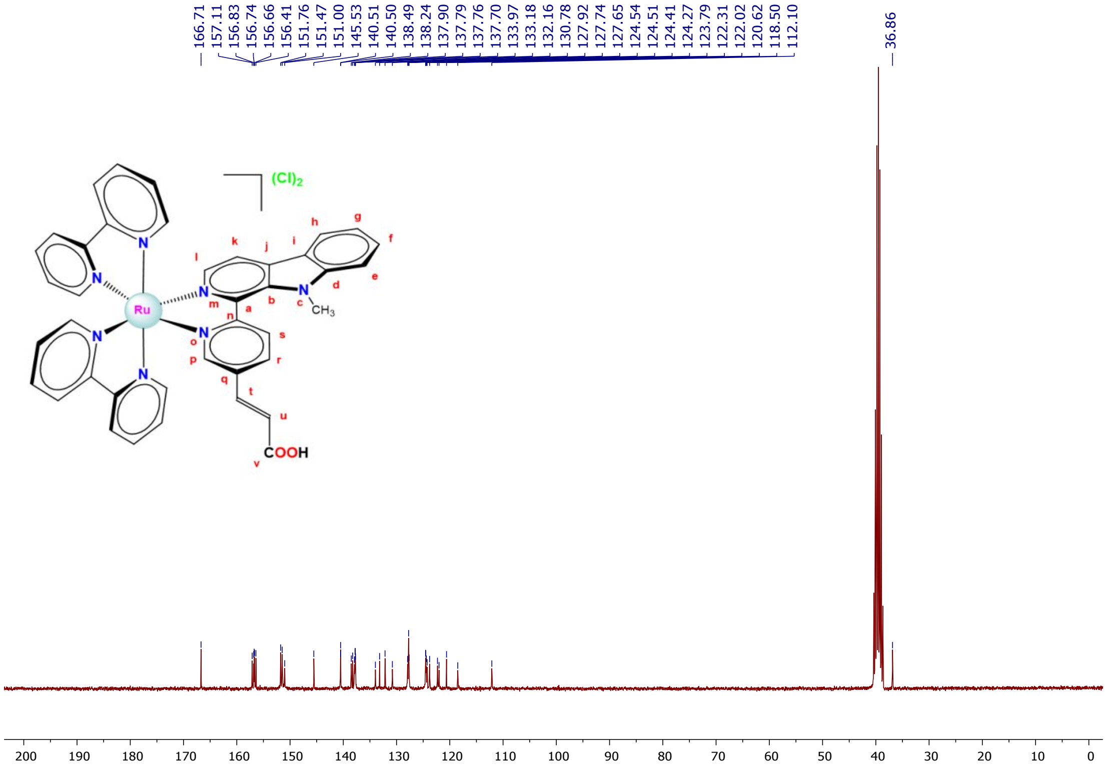

Figure S24. $^{13}C\{^{1}H\}$ NMR (75 MHz, DMSO-d_6, 25 °C) spectrum of **Ru-H**.

4. High-resolution mass spectrometry

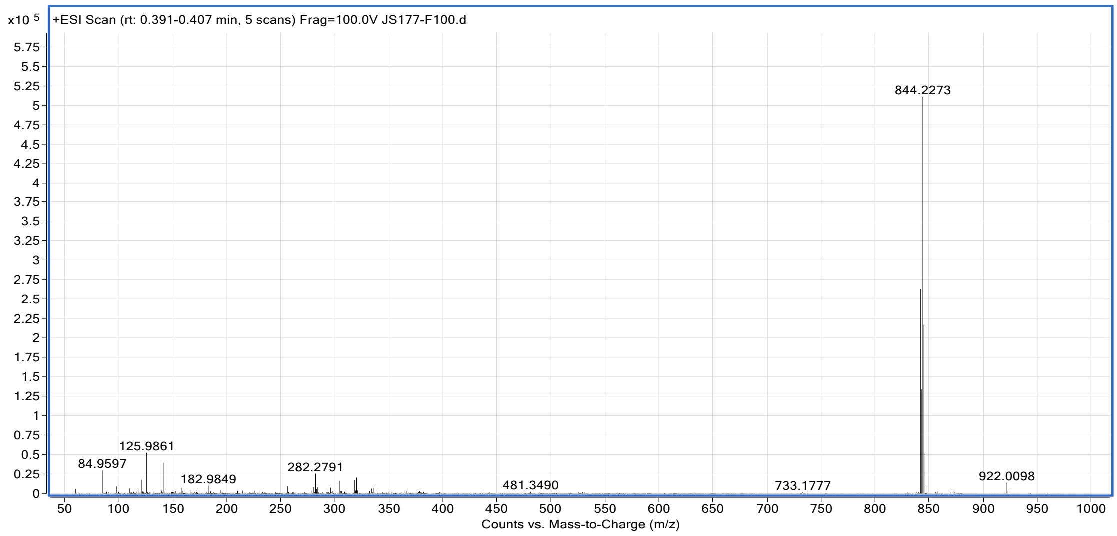

Figure S25. HR-ESI(+) mass spectrum of complex **Ir-Me**.

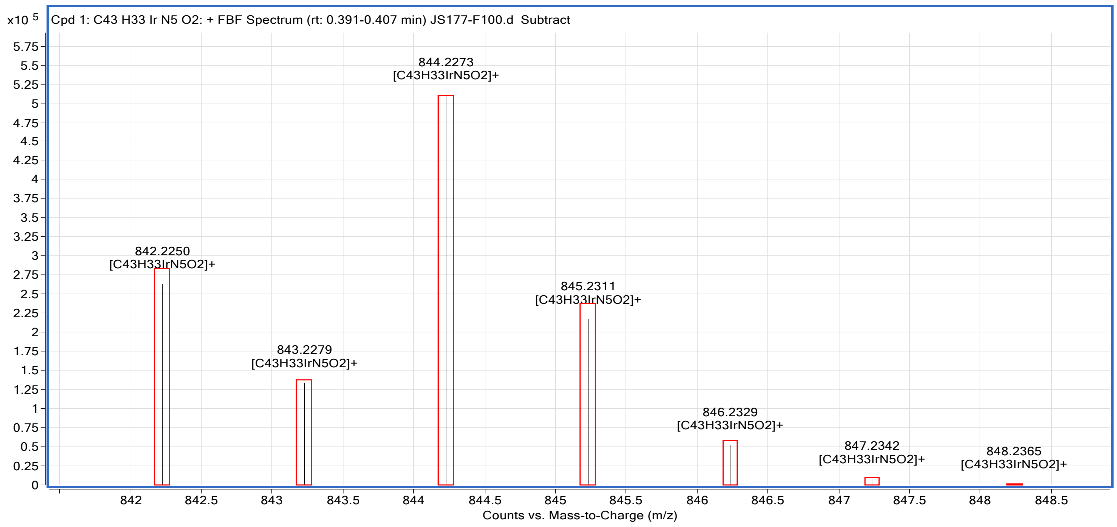

Figure S26. Experimental (black lines) and calculated (red squares) isotopic distribution of the main set of peaks recorded in the HR ESI(+) mass spectrum of complex **Ir-Me**.

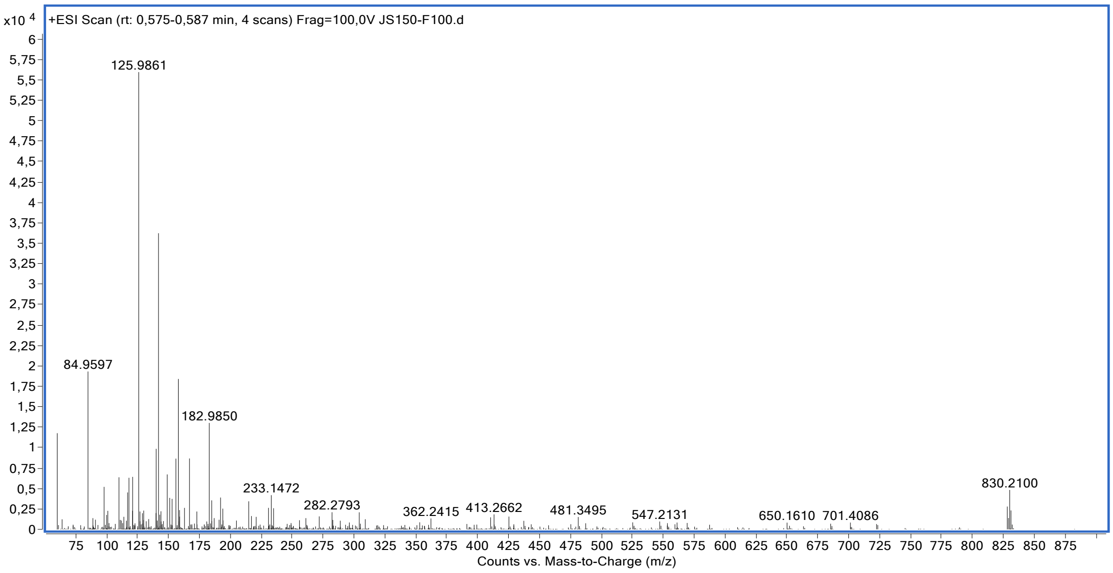

Figure S27. HR-ESI(+) mass spectrum of complex **Ir-H**.

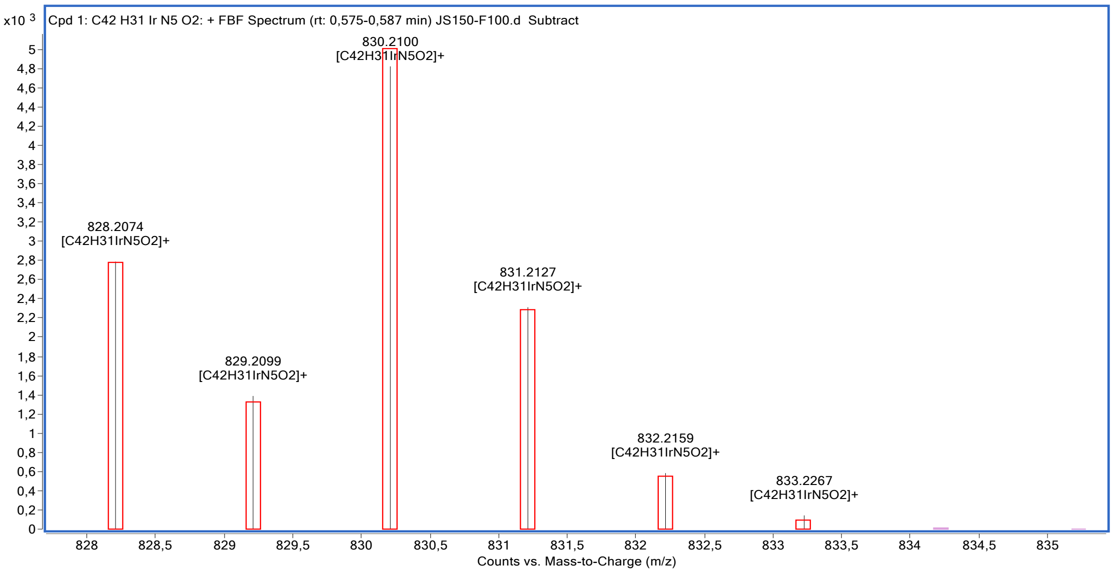

Figure S28. Experimental (black lines) and calculated (red squares) isotopic distribution of the main set of peaks recorded in the HR ESI(+) mass spectrum of complex **Ir-H**.

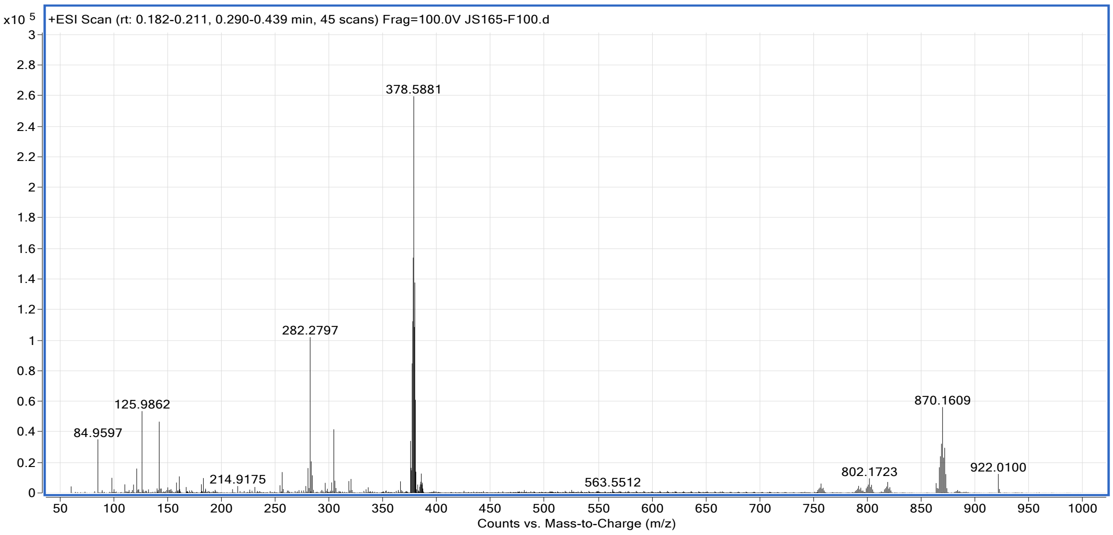

Figure S29. HR-ESI(+) mass spectrum of complex **Ru-Me**.

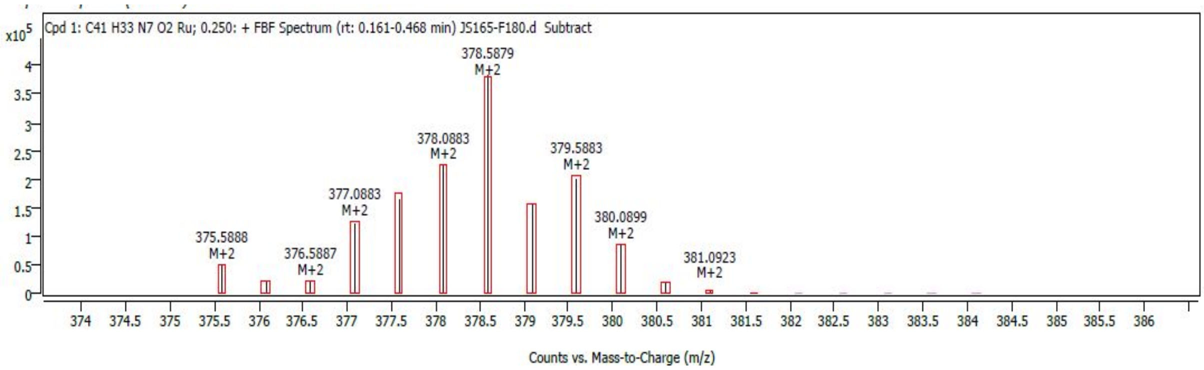

Figure S30. Experimental (black lines) and calculated (red squares) isotopic distribution of the main set of peaks recorded in the HR ESI(+) mass spectrum of complex **Ru-Me**.

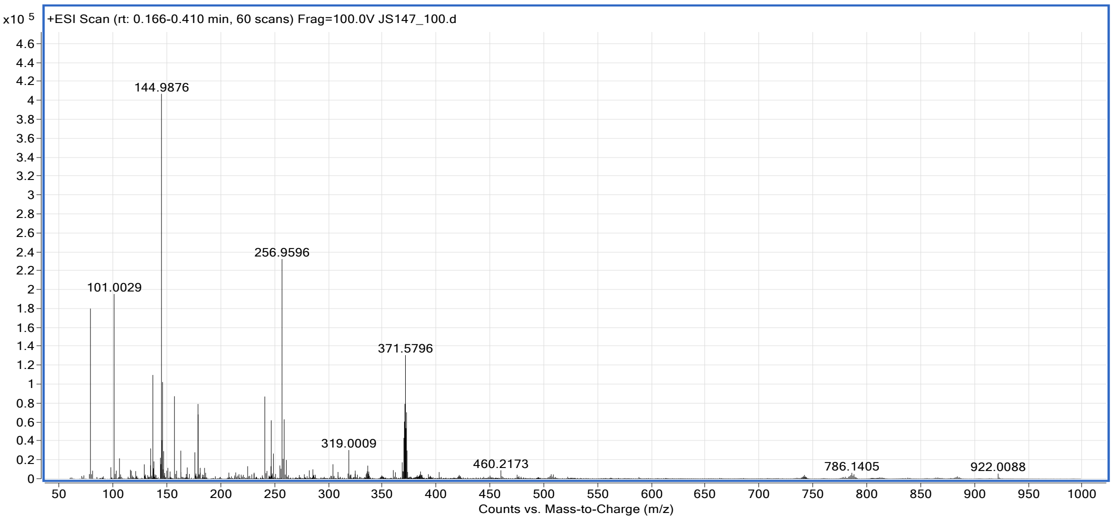

Figure S31. HR-ESI(+) mass spectrum of complex **Ru-H**.

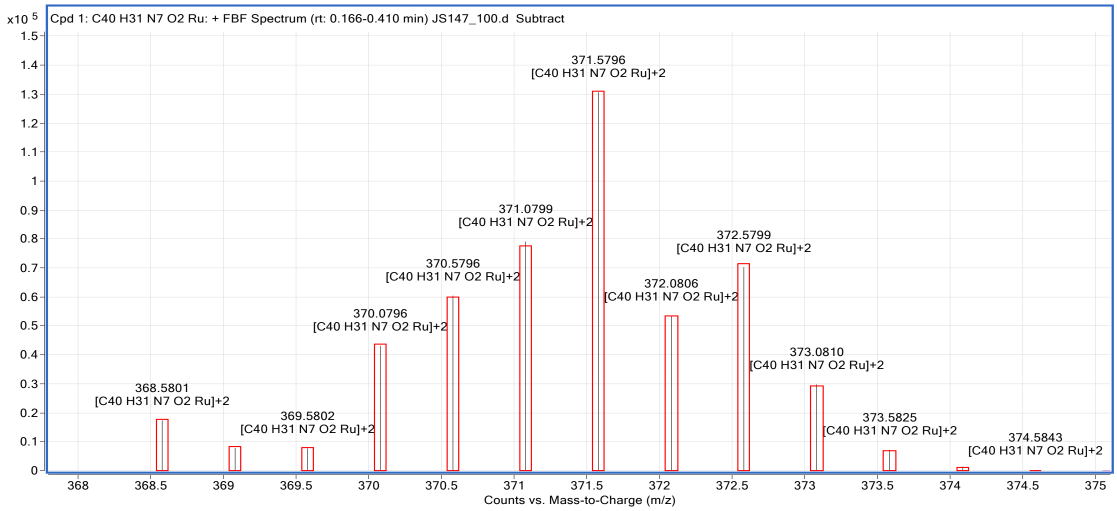

Figure S32. Experimental (black lines) and calculated (red squares) isotopic distribution of the main set of peaks recorded in the HR ESI(+) mass spectrum of complex **Ru-H**.

5. Synthesis of metallopeptides Ir-BN and Ru-BN

Synthesis of metallopeptides Ir-BN and Ru-BN. Metallopeptides **Ir-BN** and **Ru-BN** were manually synthesised on solid phase following an orthogonal Fmoc/*t*Bu strategy. Reactions were carried out in polypropylene syringes containing a polyethylene filter. An aminomethyl ChemMatrix® (0.69 mmol/g) resin was used as solid support, Fmoc-Rink-amide as linker, *tert*-butyloxycarbonyl (Boc) as side-chain protecting group for Trp, trityl (Tr) for Gln, Asn and His, and 2,2,4,6,7-pentamethyldihydrobenzofuran-5-sulfonyl (Pbf) for Arg. The ChemMatrix resin was swollen with CH_3OH (2 × 1 min), DMF (2 × 1 min), CH_2Cl_2 (2 × 1 min), CH_2Cl_2/TFA (99:1) (3 × 1 min), CH_2Cl_2/DIPEA (19:1) (3 × 1 min), CH_2Cl_2 (3 × 1 min) and DMF (6 × 1 min). Then, the resin was treated with the Fmoc-Rink-amide linker (3 equiv), DIC (3 equiv) and Oxyma (3 equiv) in DMF overnight, followed by washes with DMF (6 × 1 min) and CH_2Cl_2 (3 × 1 min). The completion of the reaction was checked with the Kaiser test.[39] Peptide elongation was carried out through sequential steps of Fmoc group removal, coupling of the amino acids and washes. The Fmoc group was removed with piperidine/DMF (3:7, 2 + 10 min). Couplings of the amino acids were carried out by treating the resin with the corresponding amino acid (3 equiv), DIC (3 equiv) and Oxyma (3 equiv) in DMF overnight. These reactions were monitored with the Kaiser test. After each coupling and Fmoc group removal, the resin was washed with DMF (6 × 1 min) and CH_2Cl_2 (3 × 1 min). After the 6th coupling, *N*-methyl-2-pyrrolidinone (NMP) was used instead of DMF. Once the peptidyl sequence was completed, the Fmoc group was removed and the peptidyl resin was treated with **Ir-H** or **Ru-H** (1.2 equiv), DIC (1.2 equiv) and Oxyma (1.2 equiv) in DMSO overnight. These reactions were monitored with the Kaiser test. After washes with DMF (6 × 1 min) and CH_2Cl_2 (3 × 1 min), metallopeptides were cleaved from the resin by treatment with TFA/H_2O/TIS (95:2.5:2.5) for 2 h at room temperature. Then, TFA was evaporated by bubbling N_2 into the solution. The crude metallopeptides were precipitated with cold diethyl ether (−20 °C) and collected by centrifugation. This procedure was performed three times. Finally, metallopeptides were dissolved in H_2O/CH_3CN (50:50 v/v containing 0.1% TFA), lyophilized, analyzed by HPLC, purified by reversed-phase column chromatography and characterized by mass spectrometry.

Ir-BN. HPLC (λ = 220 nm) t_R = 7.41 min (>99% purity); **ESI-MS** (*m/z*): 1151.6 $[M+H]^{2+}$, 768.1 $[M+2H]^{3+}$; **ESI-HRMS** (*m/z*) calcd. for $C_{109}H_{137}N_{28}O_{17}Ir$ $[M+H]^{2+}$ 1151.5169, found 1151.5171; calcd. for $C_{109}H_{138}N_{28}O_{17}Ir$ $[M+2H]^{3+}$ 768.0137, found 768.0139.

Ru-BN. HPLC (λ = 220 nm) t_R = 5.91 min (>99% purity); **ESI-MS** (*m/z*): 1107.6 $[M]^{2+}$. **ESI-HRMS** (*m/z*) calcd. for $C_{107}H_{136}N_{30}O_{17}Ru$ $[M]^{2+}$ 1107.4869, found 1107.4875; calcd. for $C_{107}H_{137}N_{30}O_{17}Ru$ $[M+H]^{3+}$ 738.6603, found 738.6624.

6. Characterization of metallopeptides Ir-BN and Ru-BN

Metallopeptide Ir-BN

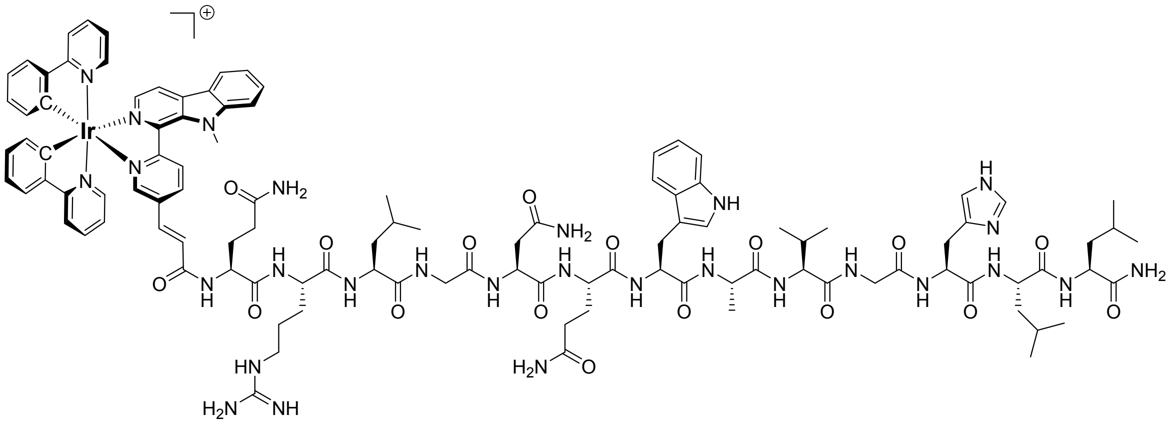

Ir-Gln-Arg-Leu-Gly-Asn-Gln-Trp-Ala-Val-Gly-His-Leu-Leu-NH_2

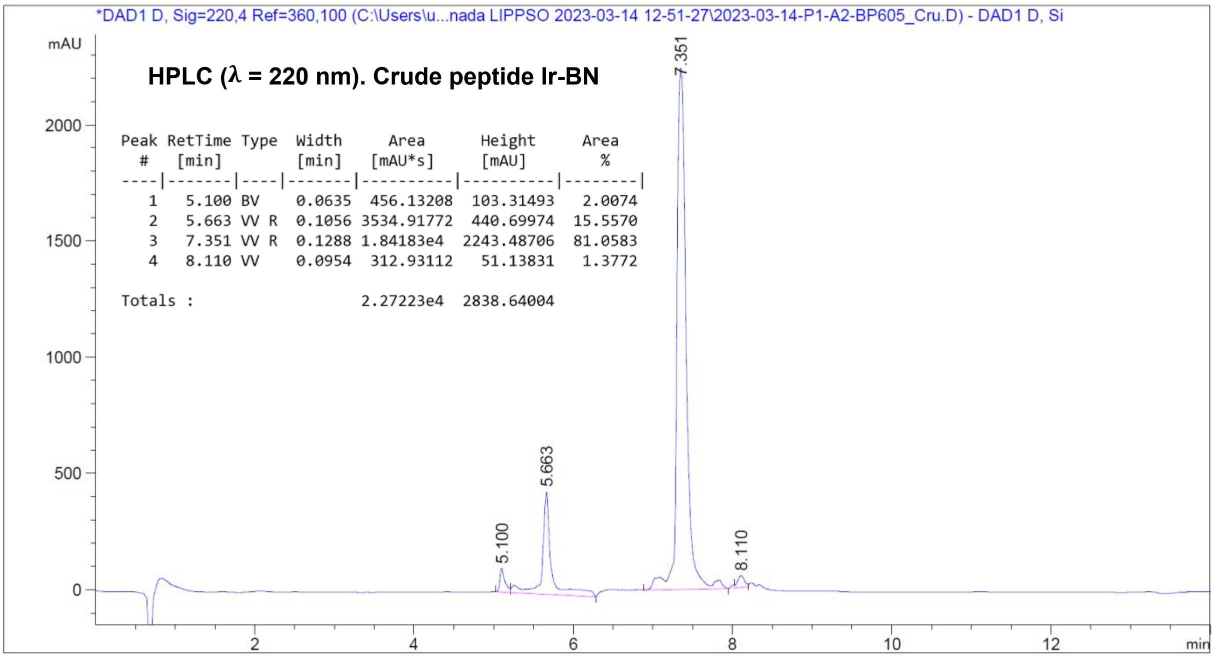

Figure S33. Reversed-phase HPLC traces at 220 nm of crude metallopeptide **Ir-BN**

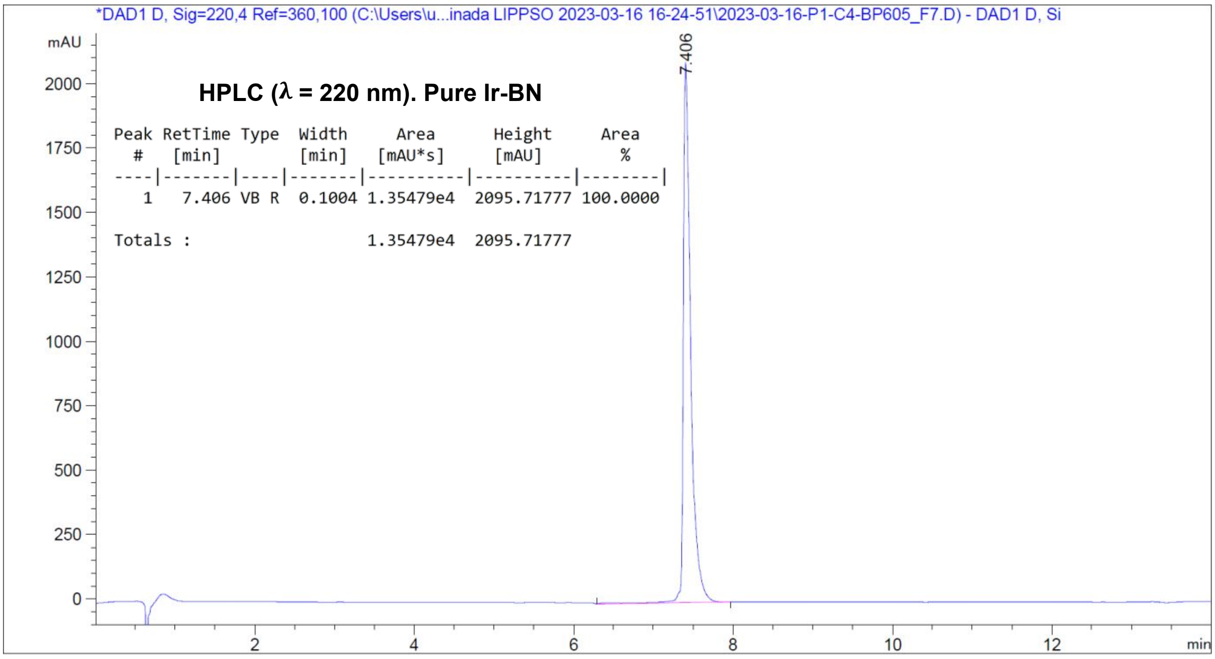

Figure S34. Reversed-phase HPLC traces at 220 nm of pure metallopeptide **Ir-BN**

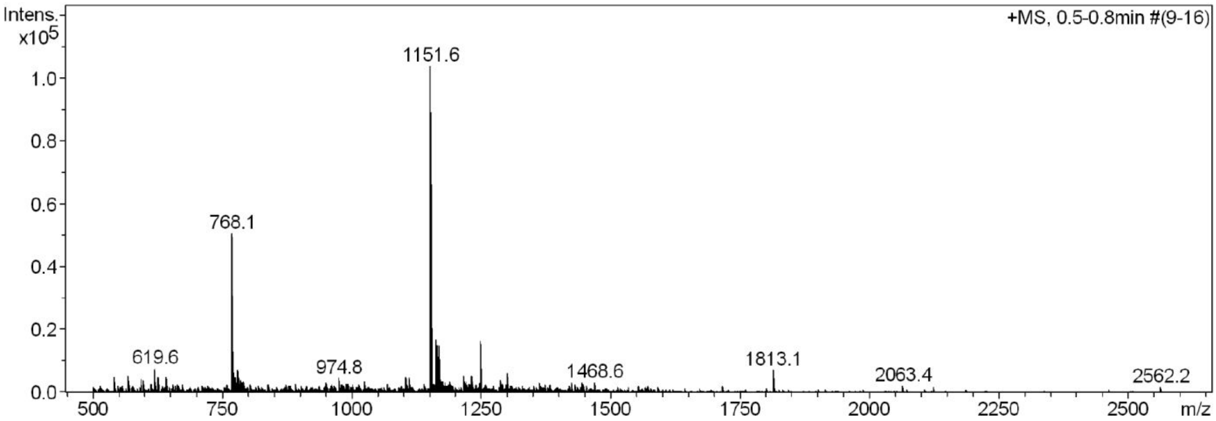

Figure S35. ESI(+) mass spectrum of metallopeptide **Ir-BN**.

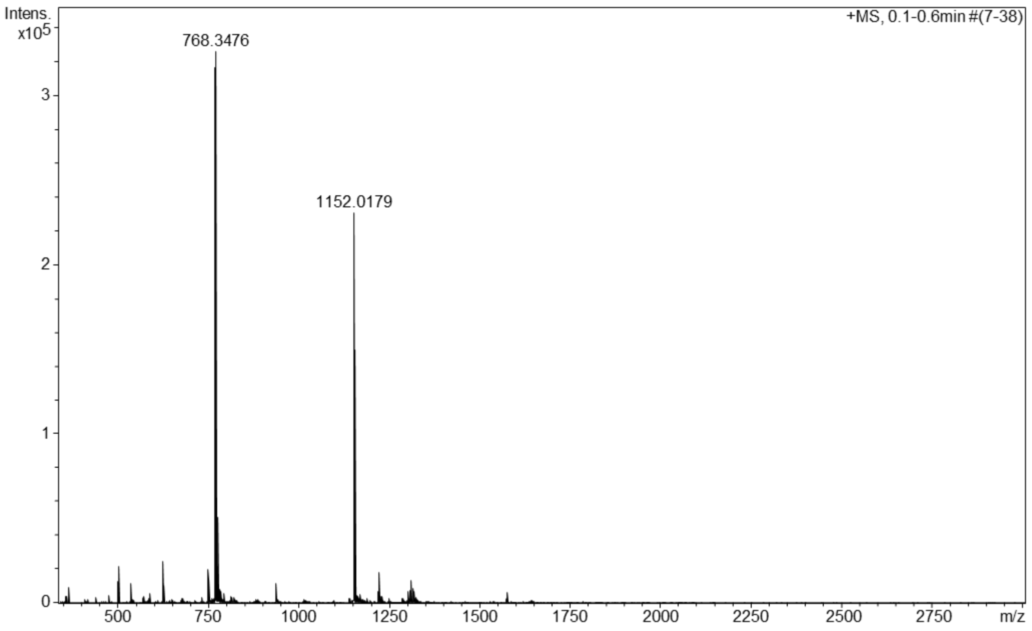

Figure S36. HR-ESI(+) mass spectrum of metallopeptide **Ir-BN**.

Table S1. Calculated and found m/z values for representative species in the HR-ESI(+) mass spectrum of **Ir-BN**.

	**Molecular formula**	**Calculated**	**Found**
**[M+H]$^{2+}$**	$C_{109}H_{137}N_{28}O_{17}Ir$	1151.5169	1151.5171
**[M+2H]$^{3+}$**	$C_{109}H_{138}N_{28}O_{17}Ir$	768.0137	768.0139

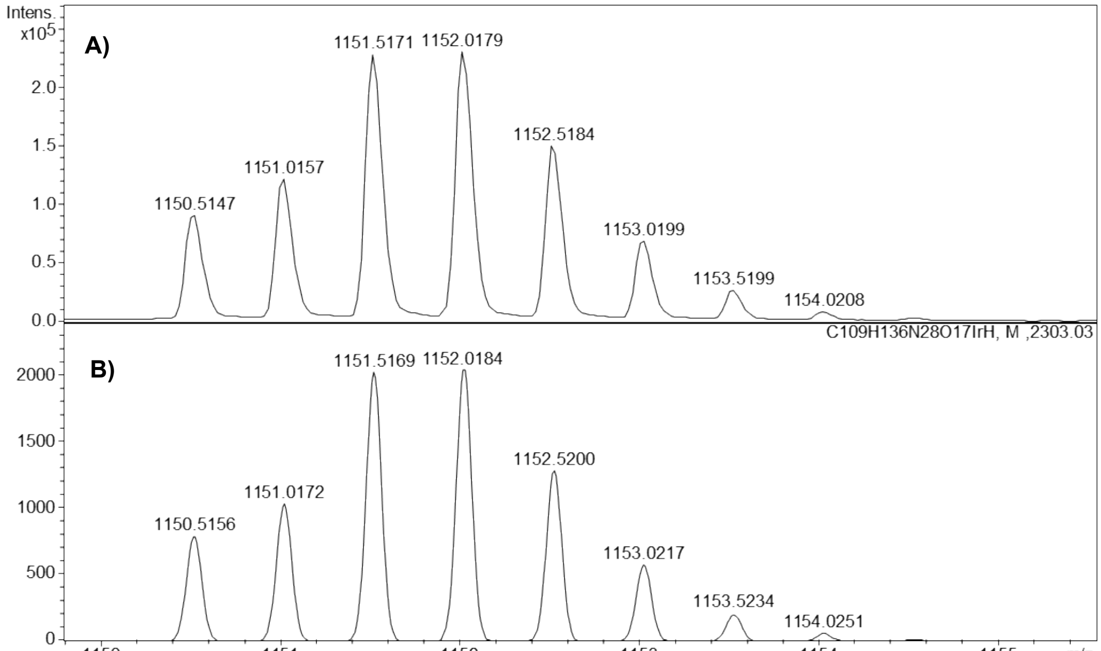

Figure S37. A) Experimental isotopic distribution of the main set of peaks recorded in the HR-ESI(+) mass spectrum of **Ir-BN**. **B)** Calculated isotopic distribution for $C_{109}H_{137}N_{28}O_{17}Ir$ $[M+H]^{2+}$.

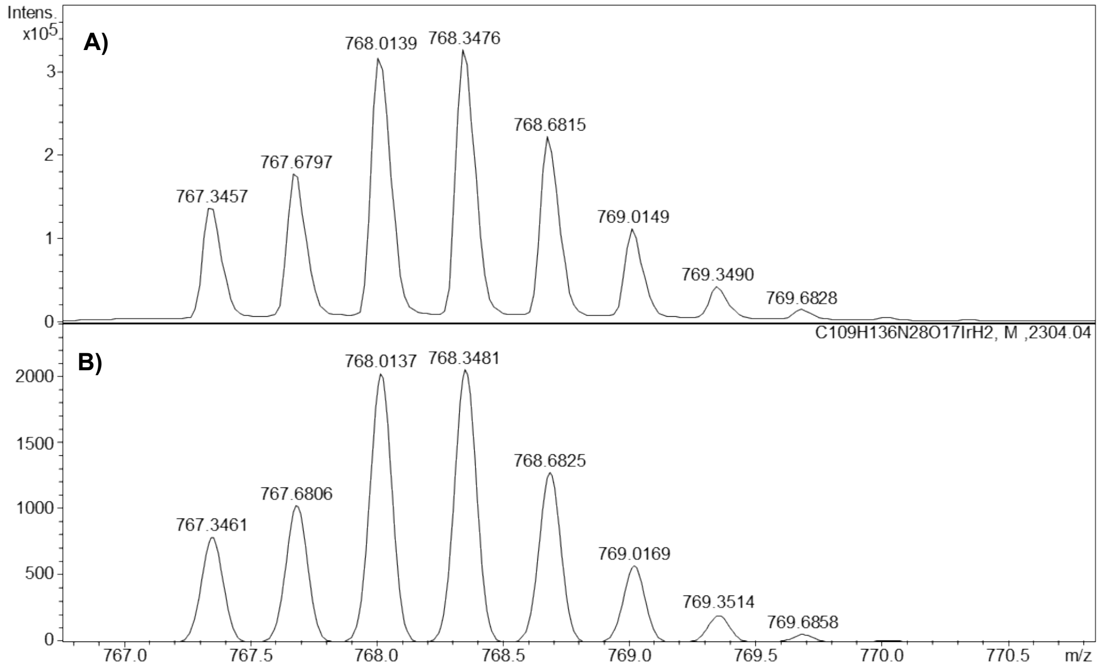

Figure S38. A) Experimental isotopic distribution of the main set of peaks recorded in the HR-ESI(+) mass spectrum of **Ir-BN**. **B)** Calculated isotopic distribution for $C_{109}H_{137}N_{28}O_{17}Ir$ $[M+2H]^{3+}$.

Ru-BN

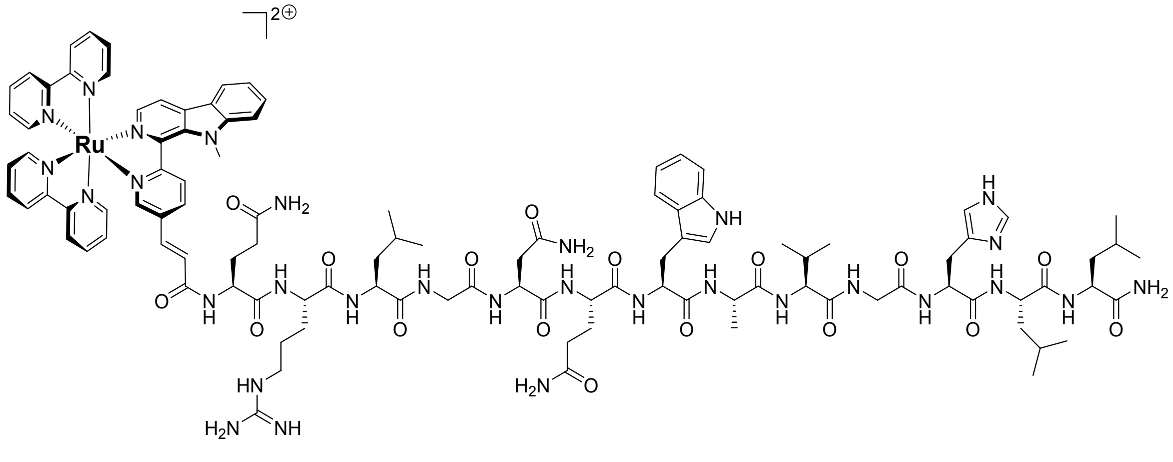

Ru-Gln-Arg-Leu-Gly-Asn-Gln-Trp-Ala-Val-Gly-His-Leu-Leu-NH_2

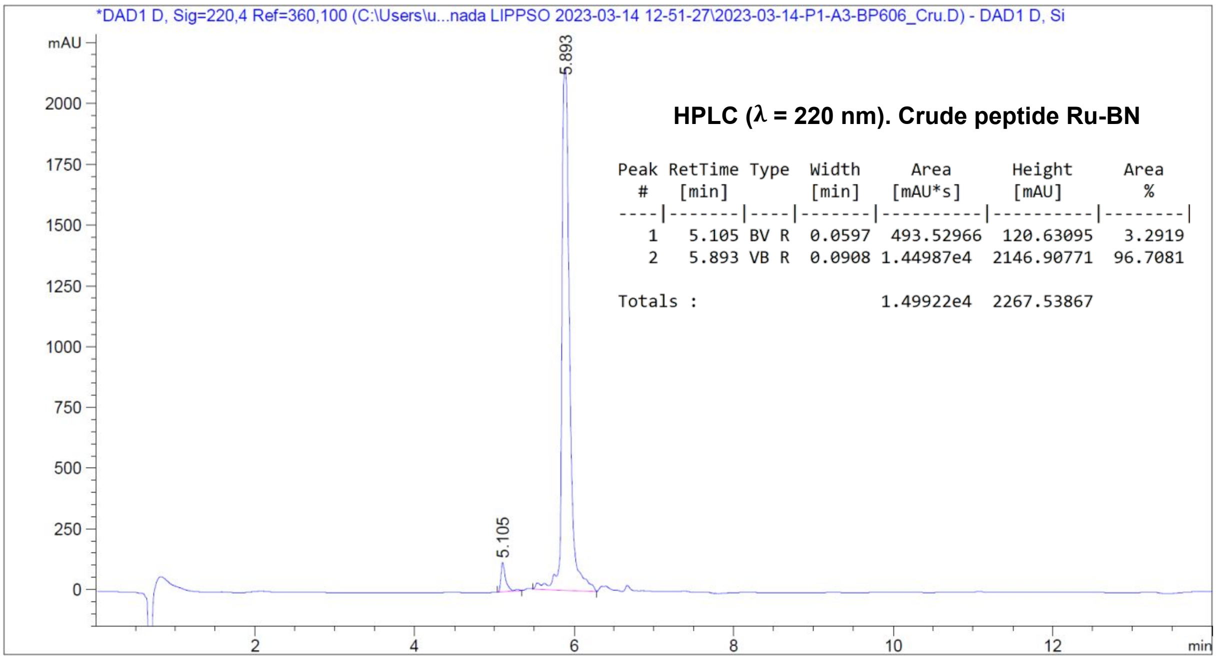

HPLC (λ = 220 nm). Crude peptide Ru-BN

Peak #	RetTime [min]	Type	Width [min]	Area [mAU*s]	Height [mAU]	Area %
1	5.105	BV R	0.0597	493.52966	120.63095	3.2919
2	5.893	VB R	0.0908	1.44987e4	2146.90771	96.7081
Totals :				1.49922e4	2267.53867	

Figure S39. Reversed-phase HPLC traces at 220 nm of crude metallopeptide **Ru-BN**.

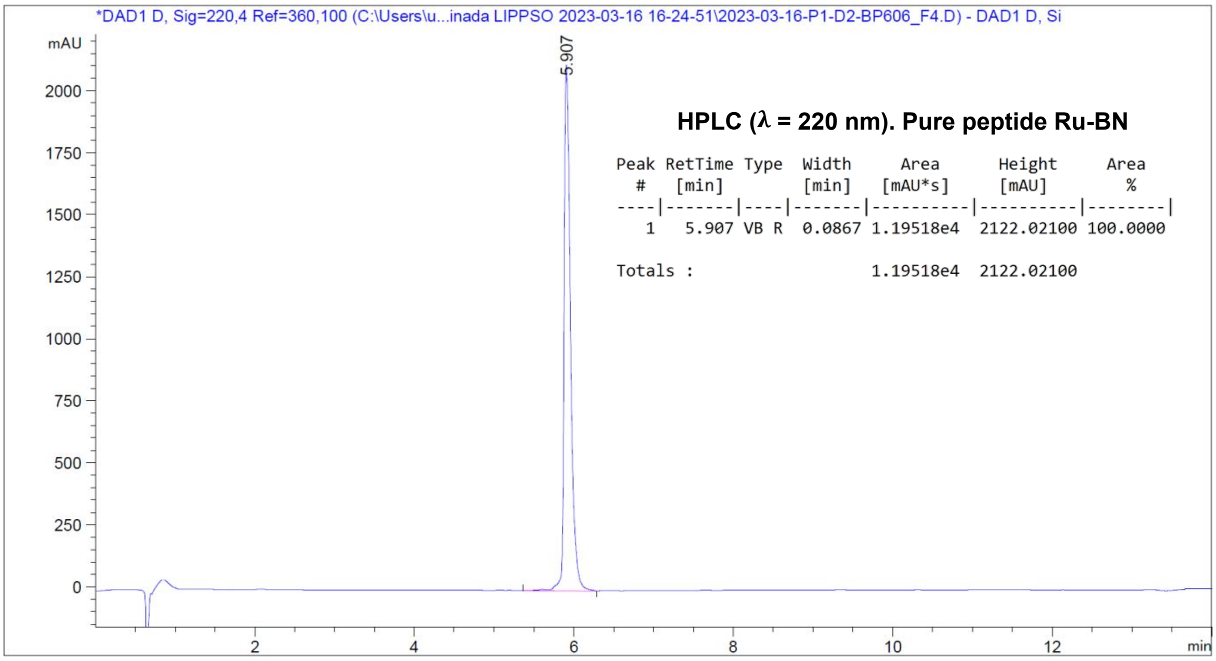

HPLC (λ = 220 nm). Pure peptide Ru-BN

Peak #	RetTime [min]	Type	Width [min]	Area [mAU*s]	Height [mAU]	Area %
1	5.907	VB R	0.0867	1.19518e4	2122.02100	100.0000
Totals :				1.19518e4	2122.02100	

Figure S40. Reversed-phase HPLC traces at 220 nm of crude metallopeptide **Ru-BN**.

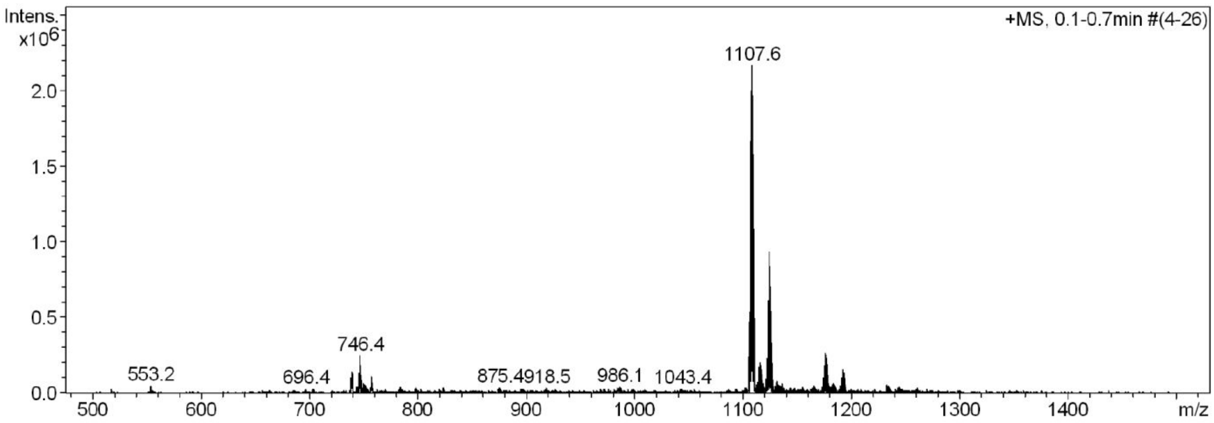

Figure S41. ESI(+) mass spectrum of metallopeptide **Ru-BN**.

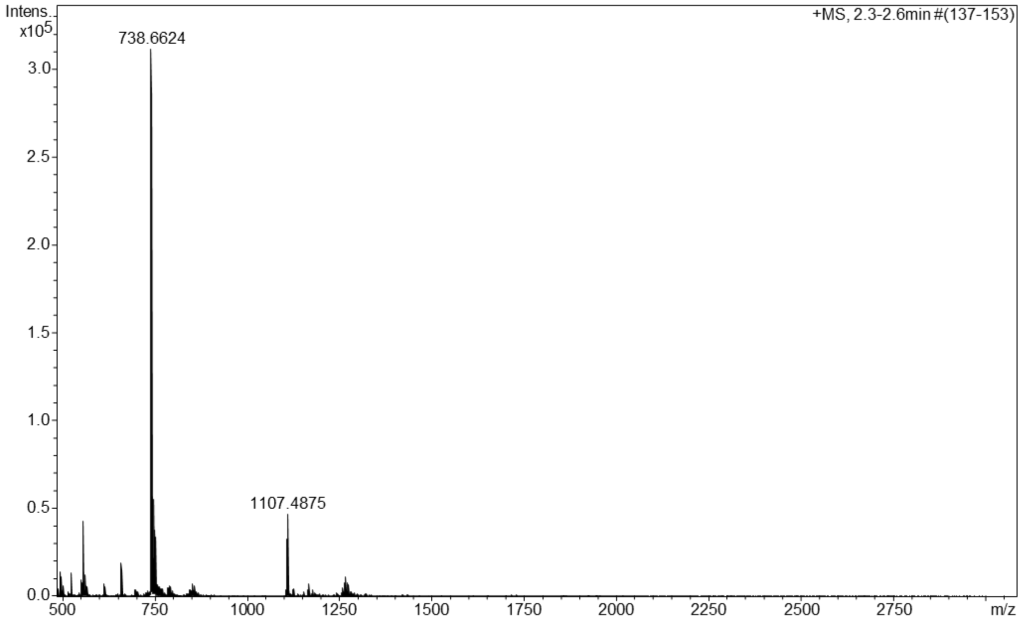

Figure S42. HR-ESI(+) mass spectrum of metallopeptide **Ru-BN**.

Table S2. Calculated and found m/z values for representative species in the HR-ESI(+) mass spectrum of **Ru-BN**.

	Molecular formula	Calculated	Found
**$[M]^{2+}$**	$C_{107}H_{136}N_{30}O_{17}Ru$	1107.4869	1107.4875
**$[M+H]^{3+}$**	$C_{107}H_{137}N_{30}O_{17}Ru$	738.6603	738.6624

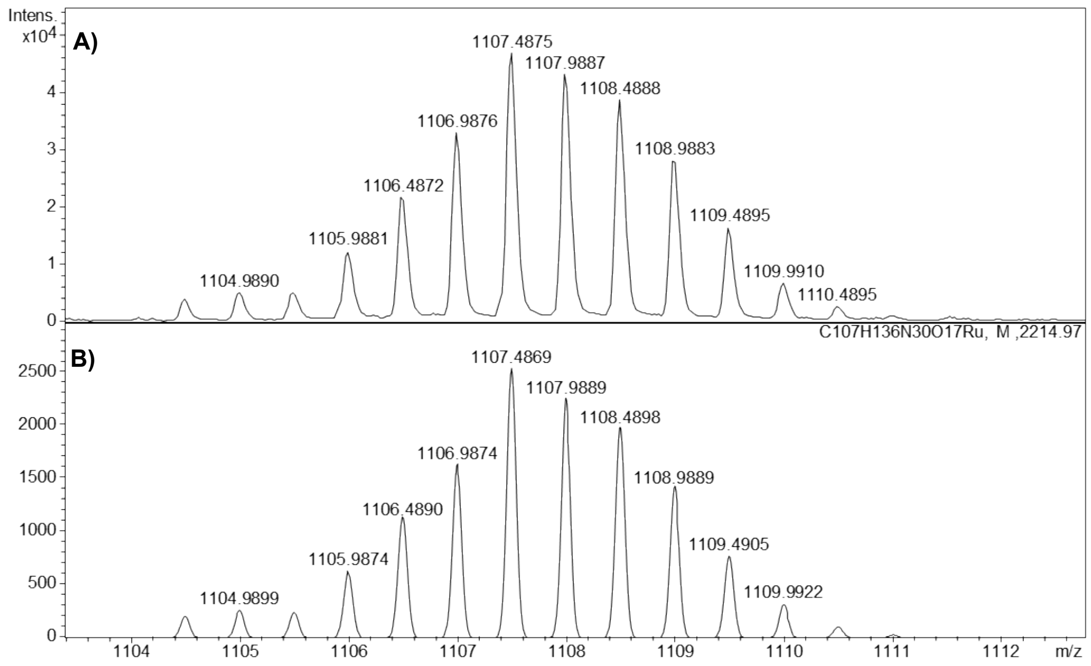

Figure S43. A) Experimental isotopic distribution of the main set of peaks recorded in the HR-ESI(+) mass spectrum of **Ru-BN**. **B)** Calculated isotopic distribution for $C_{107}H_{136}N_{30}O_{17}Ru$ $[M]^{2+}$.

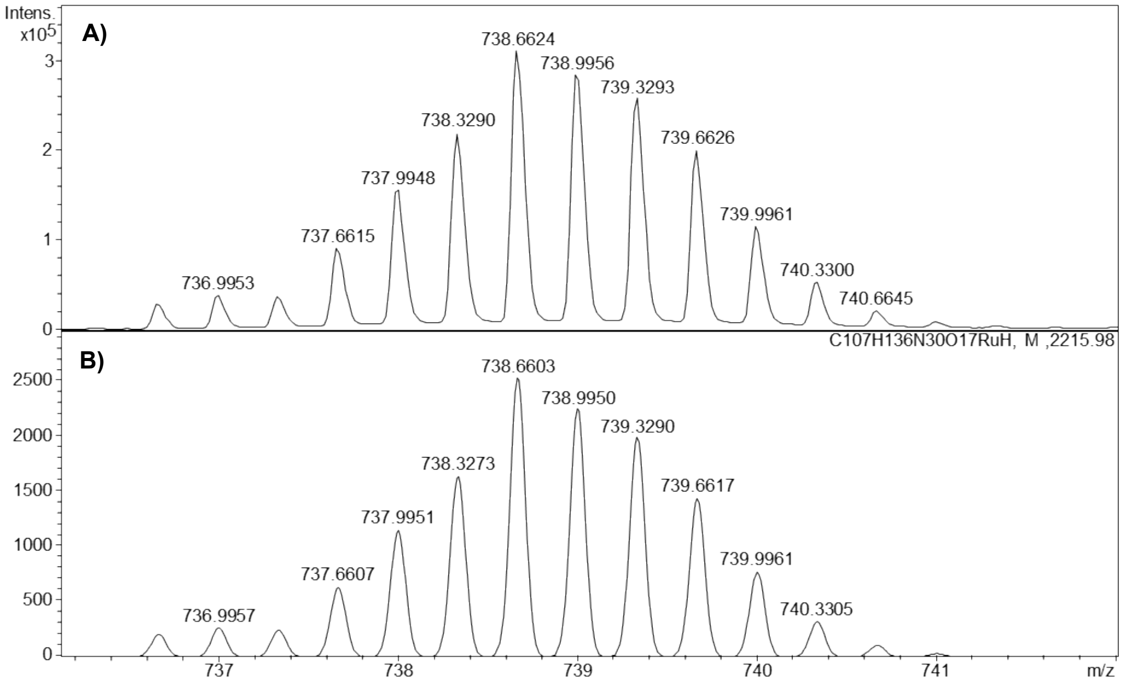

Figure S44. A) Experimental isotopic distribution of the main set of peaks recorded in the HR-ESI(+) mass spectrum of **Ru-BN**. **B)** Calculated isotopic distribution for $C_{107}H_{137}N_{30}O_{17}Ru$ $[M+H]^{3+}$.

7. X-ray diffraction

Table S3. Crystal data and structure refinement for **[Ir-Me]PF$_6$**.

Identification code	1_a
Empirical formula	$C_{43}H_{33}F_6IrN_5O_2P$
Formula weight	988.91
Temperature/K	230.0
Crystal system	monoclinic
Space group	$P2_1/c$
a/Å	12.1714(7)
b/Å	19.1141(11)
c/Å	19.0153(11)
α/°	90
β/°	107.682(2)
γ/°	90
Volume/Å^3	4214.8(4)
Z	4
ρ_{calc}g/cm^3	1.558
μ/mm^1	7.082
F(000)	1952.0
Crystal size/mm^3	0.06 × 0.04 × 0.03
Radiation	CuKα (λ = 1.54178)
2Θ range for data collection/°	6.722 to 145.658
Index ranges	-14 ≤ h ≤ 15, -23 ≤ k ≤ 23, -23 ≤ l ≤ 23
Reflections collected	122890
Independent reflections	8332 [R_{int} = 0.0588, R_{sigma} = 0.0213]
Data/restraints/parameters	8332/0/525
Goodness-of-fit on F^2	1.099
Final R indexes [I>=2σ (I)]	R_1 = 0.0400, wR_2 = 0.0878
Final R indexes [all data]	R_1 = 0.0517, wR_2 = 0.0995
Largest diff. peak/hole / e Å^{-3}	2.04/-1.14

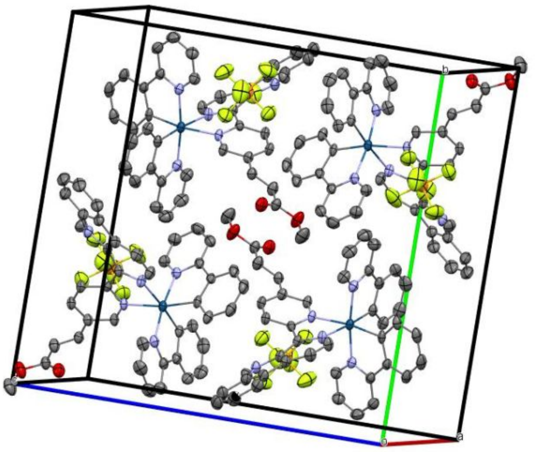

Figure S45. Cell unit of **[Ir-Me]PF$_6$** containing two pairs of enantiomers (Δ,Λ).

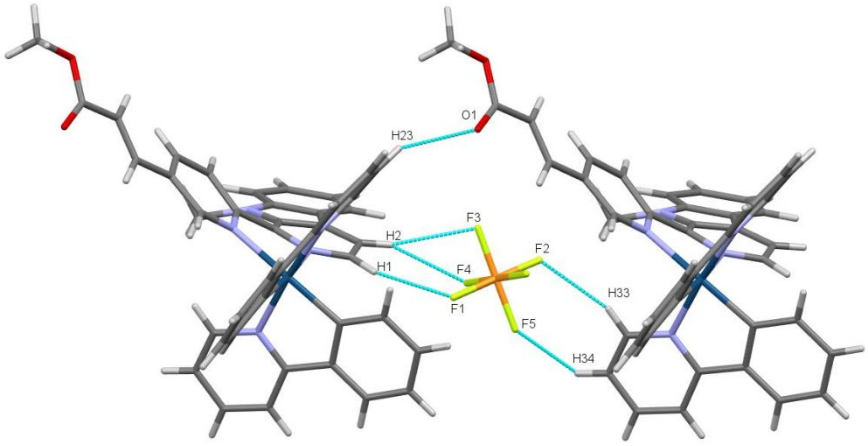

Figure S46. Hydrogen bonding interactions involving the counteranion (PF_6^-) and [**Ir-Me**]$^+$.

Table S4. Calculated parameters for some of the hydrogen bonding interactions involving the counterion PF_6^- in the crystal structure of **[Ir-Me]$^+$**.

Interaction	d(D---A) (Å)	d(H---A) (Å)	D-H---A (°)	strength
C(1)H(1)—F(1)	3.210	2.543	128.25	weak
C(2)H(2)---F(3)	3.448	2.670	140.62	weak
C(2)H(2)---F(4)	3.480	2.601	158.19	weak
C(33)H(33)---F(2)	3.072	2.417	126.74	weak
C(34)H(34)---F(5)	3.072	2.417	148.86	weak
C(23)H(23)---O(1)	3.366	2.506	152.21	weak

8. Photostability

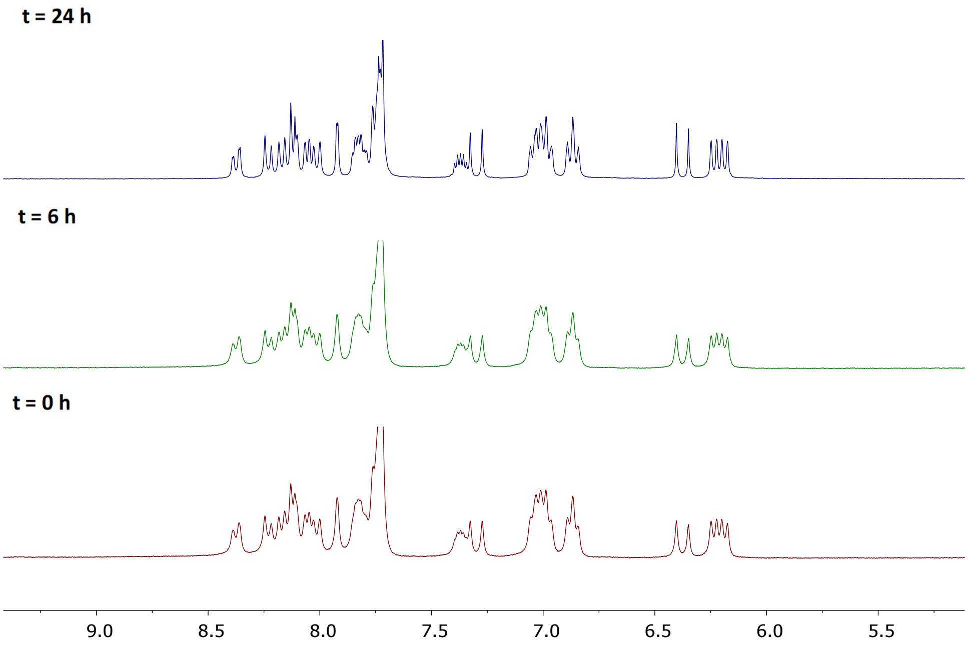

Figure S47. Evolution of the aromatic region of the ^{1}H NMR spectra of **Ir-Me** (1.5×10^{-2} M) in DMSO-d_6:D_2O (3:2, v:v) under blue light irradiation (LED, λ = 460 nm, 24 W): 1) t = 0 h, 2) t = 6 h, 3) t = 24 h.

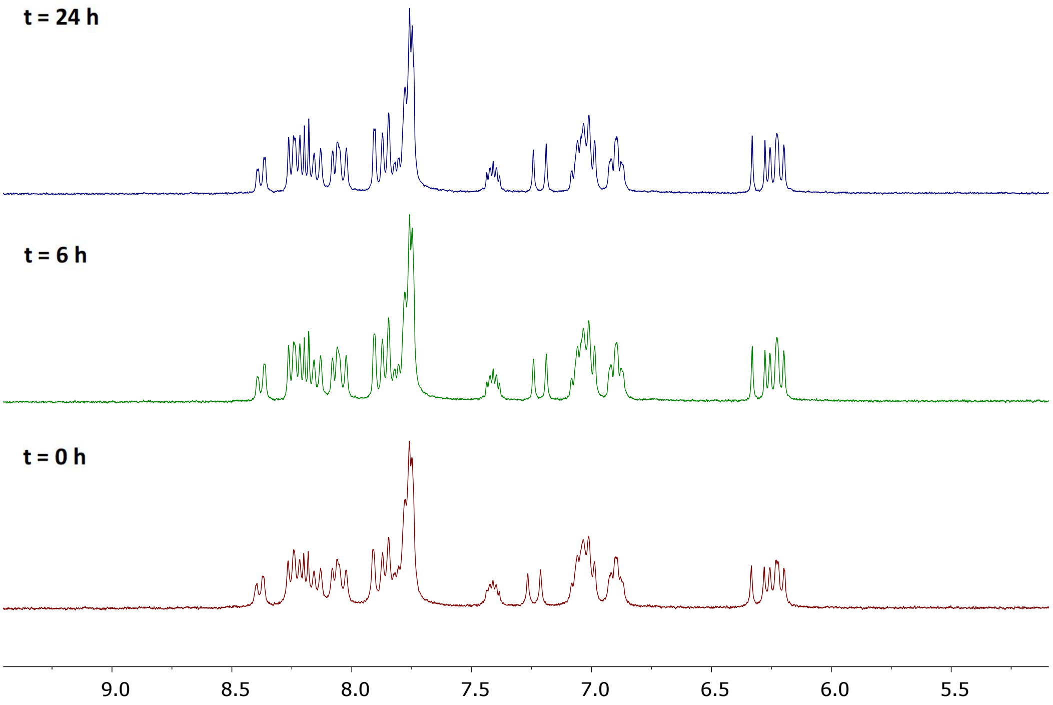

Figure S48. Evolution of the aromatic region of the ^{1}H NMR spectra of **Ir-H** (1.5×10^{-2} M) in DMSO-d_6:D_2O (3:2, v:v) under blue light irradiation (LED, λ = 460 nm, 24 W): 1) t = 0 h, 2) t = 6 h, 3) t = 24 h.

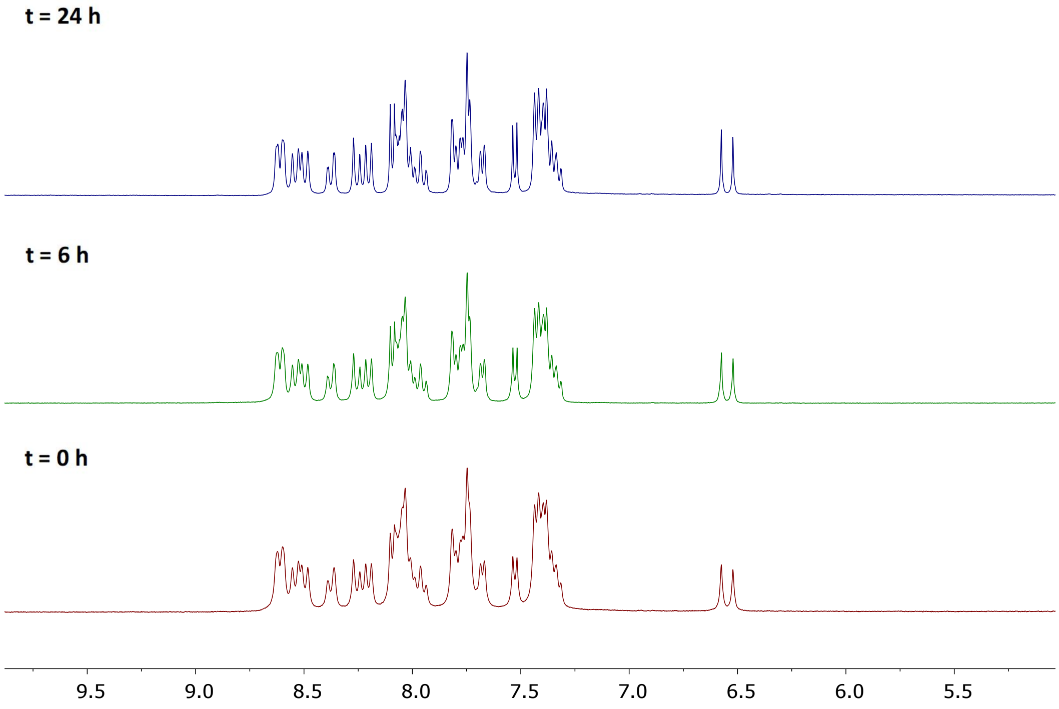

Figure S49. Evolution of the aromatic region of the ^{1}H NMR spectra of **Ru-Me** (1.5×10^{-2} M) in DMSO-d_6:D_2O (3:2, v:v) under blue light irradiation (LED, λ = 460 nm, 24 W): 1) t = 0 h, 2) t = 6 h, 3) t = 24 h.

9. Determination of pK_a

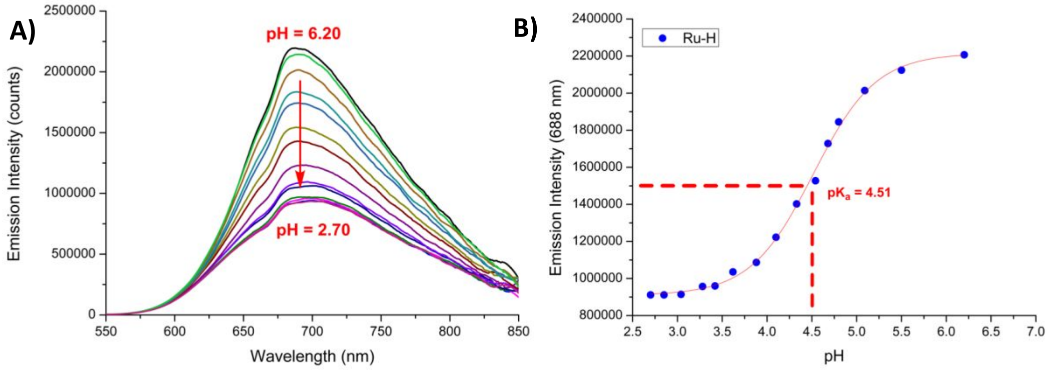

Figure S50. **A)** Overlaid emission spectra of **Ru-H** in H_2O:DMSO (99:1, v:v) (10^{-5} M) at different pH values ranging from pH 2.70 to 6.20). **B)** Plot of the emission intensity of **Ru-H** at λ = 681 nm in H_2O:DMSO (99:1, v:v) (10^{-5} M) as a function of pH (2.70 – 6.20)

10. Singlet oxygen generation

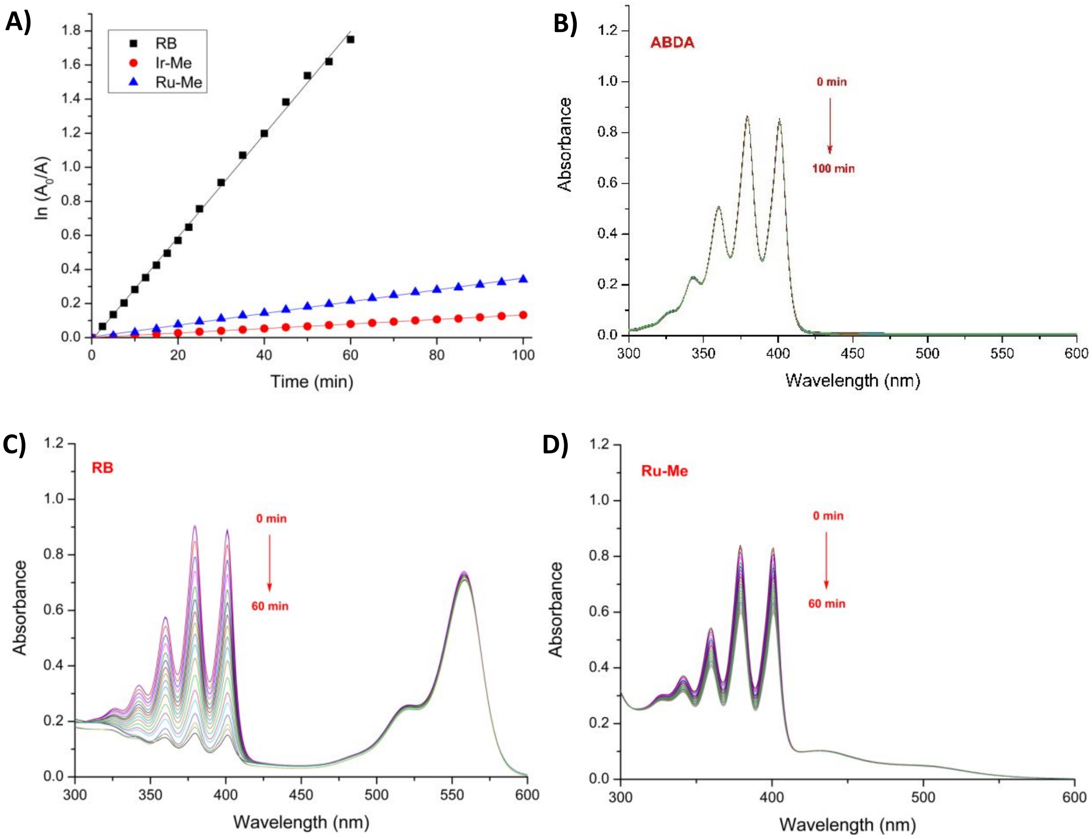

Figure S51. **A)** Comparison of the photobleaching rate of ABDA (8×10^{-5} M) in presence of **Ir-Me**, **Ru-Me** or **RB** in H_2O:DMSO (50:50) under blue light irradiation (460 nm, 24 W) at room temperature. **B)** Photobleaching of ABDA in the absence of PS. **C)** Photobleaching of ABDA in the presence of **RB** D) Photobleaching of ABDA in the presence of **Ru-Me**.

11. Photocatalytic oxidation of NADH

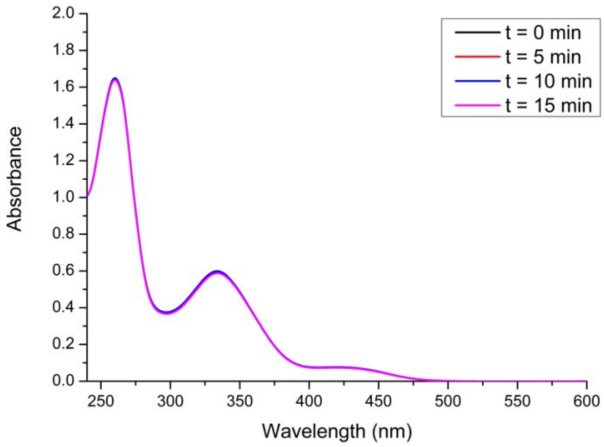

Figure S52. UV-Vis spectra for the photocatalytic oxidation of NADH (100 µM) in the presence of **Ir-Me** (5 µM) under dark conditions at room temperature

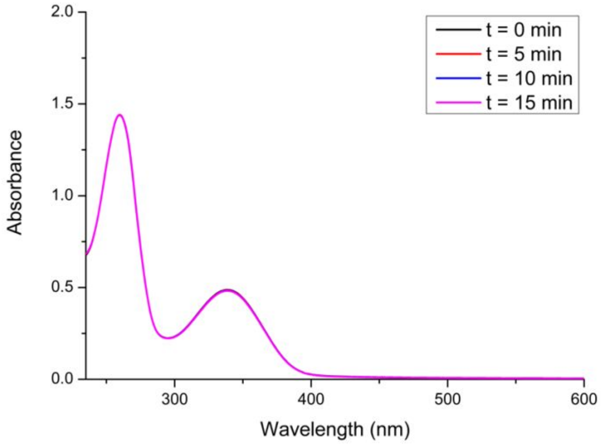

Figure S53. UV-vis spectra for the photocatalytic oxidation of NADH (100 µM) without photosensitizer under blue light irradiation (460 nm, 24 W) at room temperature.

12. <u>Bombesin receptor blocking experiments</u>

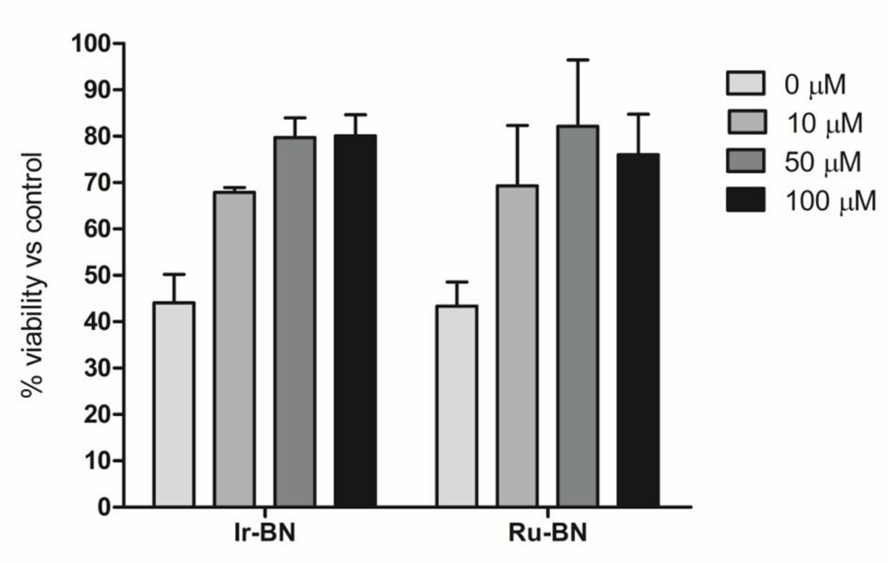

Figure S54. Bombesin receptor blockade inhibits **Ir-BN** and **Ru-BN** cytotoxicity. PC-3 cells were pretreated with the indicated concentrations of bombesin (0-100 µM) for 30 minutes to saturate bombesin receptors, followed by the addition of **Ir-BN** (10 µM) or **Ru-BN** (5 µM). After 4 hours of incubation, treatments were removed and cells were then exposed to blue light for 1 hour. Cell viability was assessed 43 hours later by MTT assays. The % of viable cells relative to controls treated with bombesin alone were calculated. Data represent mean ± SD of duplicate measurements from two independent experiments.

13. Intracellular fluorescence of the compounds

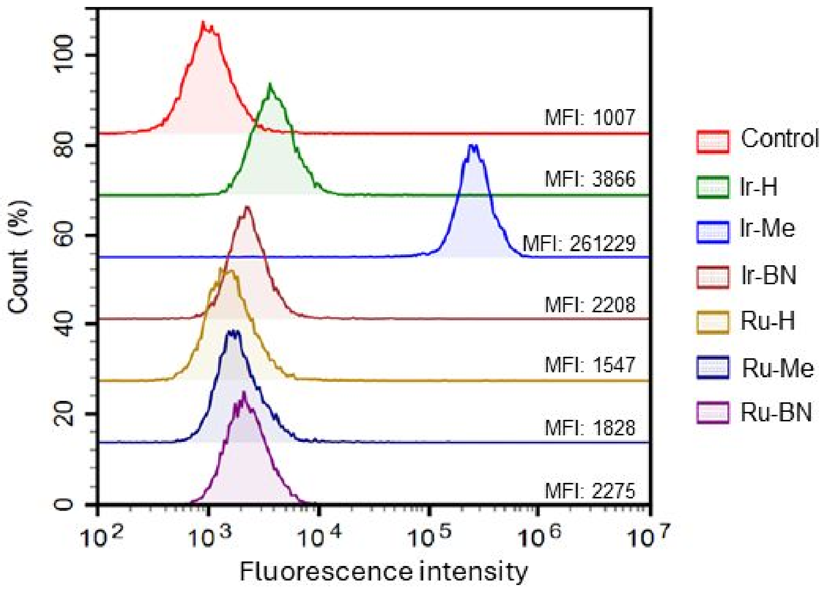

Figure S55. Intracellular fluorescence of the compounds. PC-3 cells were incubated with the metal complexes and metallopeptides at 5 µM for 4 h and the median intracellular fluorescence (MFI) of 10,000 cells at 675 nm was analyzed by flow cytometry. The histograms obtained for each compound are represented.

14. Lipophilicity and self-aggregation studies

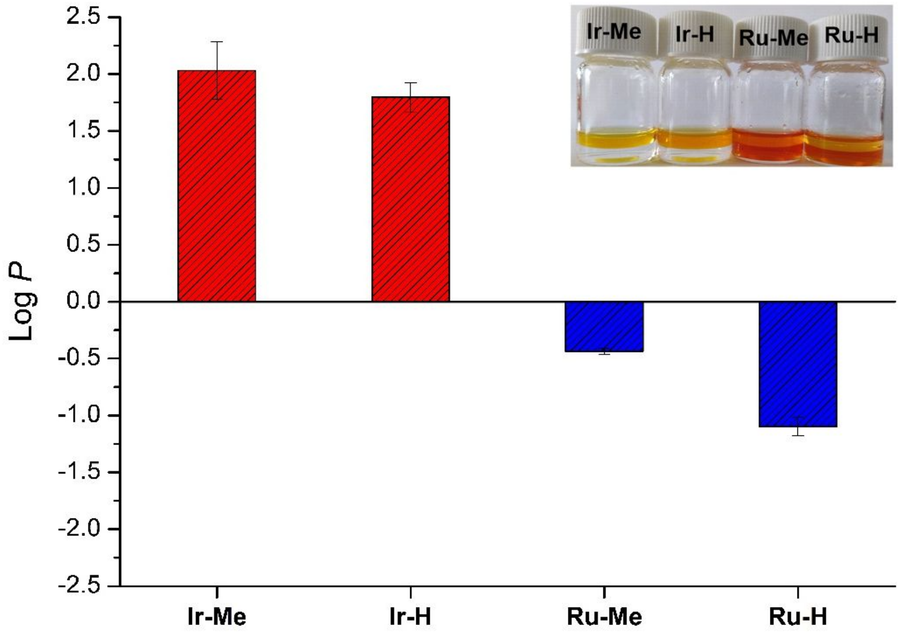

Figure S56. Log $P_{oct/PBS}$ values determined for the new Ir(III) and Ru(II) complexes. Insert: distributions of Ir(III) and Ru(II) complexes in octanol/PBS solutions.

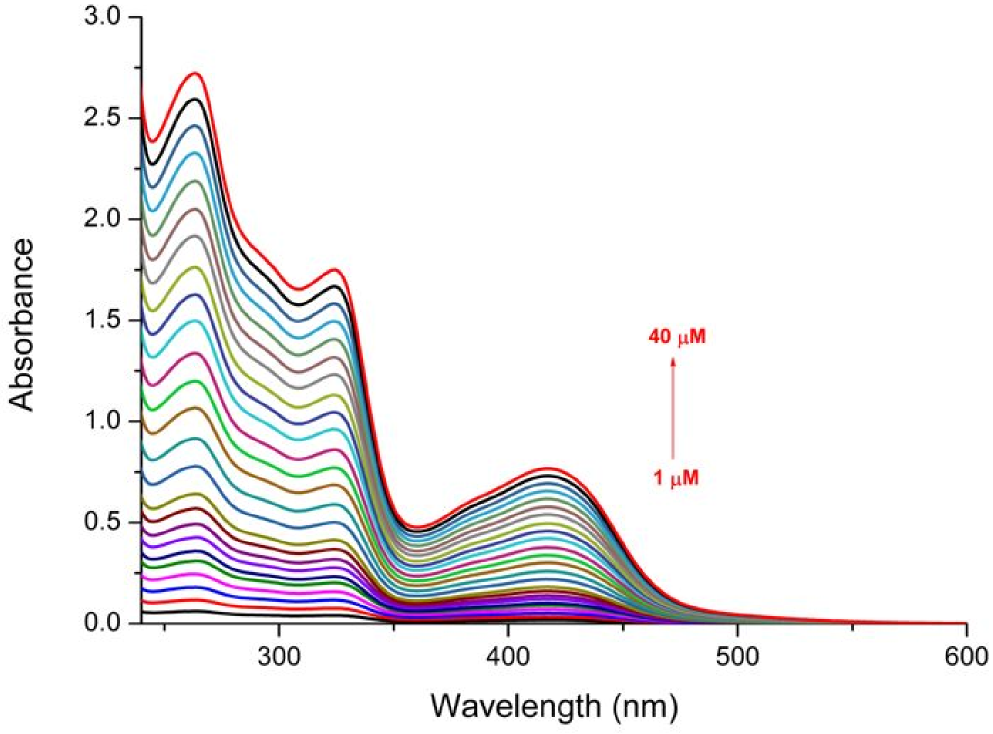

Figure S57. Overlaid absorbance spectra of **Ir-Me** (from 1 to 40 μM) in H_2O:DMSO (99:1, v:v) at room temperature.

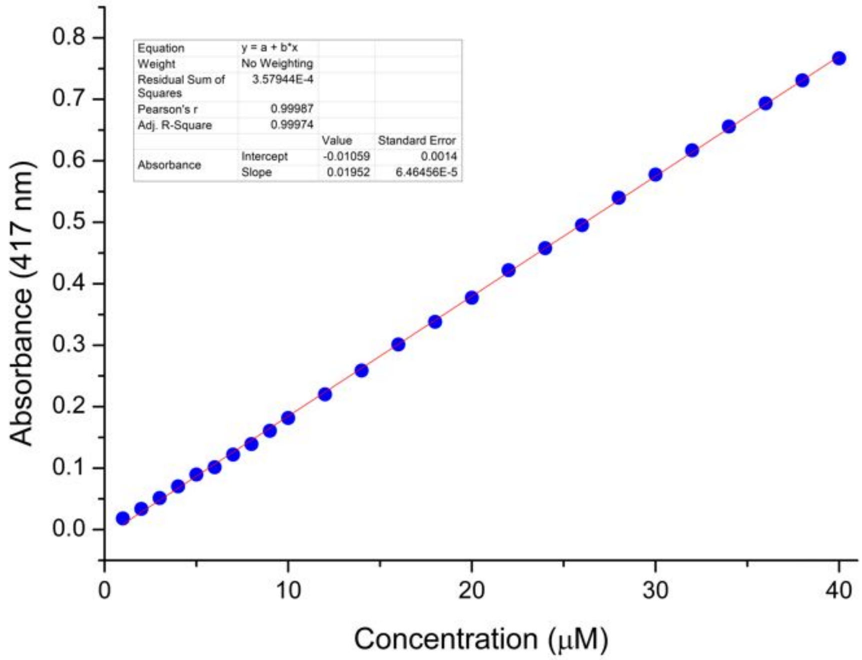

Figure S58. Absorbance at 417 nm for **Ir-Me** (from 1 to 40 μM) in H_2O:DMSO (99:1, v:v) at room temperature.

15. References

(1) *SAINT v8.37, Bruker-AXS (2016), APEX3 V2016.1.0. Madison, Wisconsin, USA.*

(2) Krause, L.; Herbst-Irmer, R.; Sheldrick, G. M.; Stalke, D. Comparison of Silver and Molybdenum Microfocus X-Ray Sources for Single-Crystal Structure Determination. *J. Appl. Crystallogr.* **2015**, *48* (1), 3–10. https://doi.org/10.1107/S1600576714022985.

(3) Dolomanov, O. V.; Bourhis, L. J. .; Gildea, R. J. .; Howard, J. A. K.; Puschmann, H. OLEX2: A Complete Structure Solution, Refinement and Analysis Program. *J. Appl. Crystallogr.* **2009**, *42*, 339–341.

(4) Sheldrick, G. M. SHELXT – Integrated Space-Group and Crystal- Structure Determination. *Acta Cryst. A* **2015**, *A71*, 3–8. https://doi.org/10.1107/S2053273314026370.

(5) Sheldrick, G. M. Crystal Structure Refinement with SHELXL. *Acta Crystallogr. Sect. C Struct. Chem.* **2015**, *C71*, 3–8. https://doi.org/10.1107/S2053229614024218.

(6) Redmond, R. W.; Gamlin, J. N. A Compilation of Singlet Oxygen Yields from Biologically Relevant Molecules. *Photochem. Photobiol.* **1999**, *70* (4), 391–475. https://doi.org/10.1111/j.1751-1097.1999.tb08240.x.

(7) Zhang, S.; Yang, W.; Lu, X.; Zhang, X.; Pan, Z.; Qu, D.-H.; Mei, D.; Mei, J.; Tian, H. Near-Infrared AIEgens with High Singlet-Oxygen Yields for Mitochondria-Specific Imaging and Antitumor Photodynamic Therapy. *Chem. Sci.* **2023**, *14* (25), 7076–7085. https://doi.org/10.1039/D3SC00588G.

(8) Gandioso, A.; Vidal, A.; Burckel, P.; Gasser, G.; Alessio, E. Ruthenium(II) Polypyridyl Complexes Containing Simple Dioxo Ligands: A Structure-Activity Relationship Study Shows the Importance of the Charge. *ChemBioChem* **2022**, *23* (19). https://doi.org/10.1002/cbic.202200398.
